# De novo assembly of genomes from long sequence reads reveals uncharted territories of *Propionibacterium freudenreichii*

**DOI:** 10.1186/s12864-017-4165-9

**Published:** 2017-10-16

**Authors:** Paulina Deptula, Pia K. Laine, Richard J. Roberts, Olli-Pekka Smolander, Helena Vihinen, Vieno Piironen, Lars Paulin, Eija Jokitalo, Kirsi Savijoki, Petri Auvinen, Pekka Varmanen

**Affiliations:** 1 Department of Food and Environmental Sciences, University of Helsinki, 00014 Helsinki, Finland; 2 Institute of Biotechnology, University of Helsinki, 00014 Helsinki, Finland; 3 New England Biolabs, Ipswich, MA 01938-2723 USA

**Keywords:** *Propionibacterium freudenreichii*, Comparative genomics, Bacteriophage, Pilus, Vitamin B12, PacBio, Complete genome, Mobile elements, Restriction and modification, CRISPR-Cas, Gras

## Abstract

**Background:**

*Propionibacterium freudenreichii* is an industrially important bacterium granted the Generally Recognized as Safe (the GRAS) status, due to its long safe use in food bioprocesses. Despite the recognized role in the food industry and in the production of vitamin B12, as well as its documented health-promoting potential, *P. freudenreichii* remained poorly characterised at the genomic level. At present, only three complete genome sequences are available for the species.

**Results:**

We used the PacBio RS II sequencing platform to generate complete genomes of 20 *P. freudenreichii* strains and compared them in detail. Comparative analyses revealed both sequence conservation and genome organisational diversity among the strains. Assembly from long reads resulted in the discovery of additional circular elements: two putative conjugative plasmids and three active, lysogenic bacteriophages. It also permitted characterisation of the CRISPR-Cas systems. The use of the PacBio sequencing platform allowed identification of DNA modifications, which in turn allowed characterisation of the restriction-modification systems together with their recognition motifs. The observed genomic differences suggested strain variation in surface piliation and specific mucus binding, which were validated by experimental studies. The phenotypic characterisation displayed large diversity between the strains in ability to utilise a range of carbohydrates, to grow at unfavourable conditions and to form a biofilm.

**Conclusion:**

The complete genome sequencing allowed detailed characterisation of the industrially important species, *P. freudenreichii* by facilitating the discovery of previously unknown features. The results presented here lay a solid foundation for future genetic and functional genomic investigations of this actinobacterial species.

**Electronic supplementary material:**

The online version of this article (10.1186/s12864-017-4165-9) contains supplementary material, which is available to authorized users.

## Background

Propionibacteria belong to the phylum Actinobacteria with high GC content (64–70%) genomes. They have a peculiar metabolism [1], characterised by the utilization of lactate and the production of propionate, acetate and carbon dioxide through the Wood-Werkmann cycle [2]. *Propionibacterium freudenreichii* is an industrially important species with Generally Recognized as Safe (GRAS) status, granted due to its long, safe use in dairy fermentations. *P. freudenreichii* is used as a secondary starter culture in the production of Swiss-type cheeses where it plays a crucial role in the formation of “eyes” by CO_2_ production and the development of the typical flavour attributed to lipolysis, release of amino acids, especially proline, and to the production of short-chain fatty acids (SCFAs): propionate and acetate [3]. Due to their antimicrobial activity, propionate or strains of *Propionibacterium* species are commonly used as food and grain preservatives to prolong the shelf-life of many products by suppressing the growth of mold and spoilage microorganisms [4]. SCFAs are among the most abundant dietary metabolites produced by the gut microbes during dietary fermentation [5] with implications in *e.g.* controlling inflammatory responses and appetite [6–8]. Notably, the SCFAs produced by *P. freudenreichii* as well as milk fermented with this species were recently shown to specifically induce apoptosis of colon cancer cells, thereby opening new avenues for microbial-based therapies [9]. In addition to SCFAs, *P. freudenreichii* produce a wide variety of compounds with implications for human health and well-being, like conjugated linoleic acid [10], vitamins [11–14], exopolysaccharides [15] and trehalose [16], and have thus potential application as cell factories for natural enrichment of food with nutraceuticals. There is an increasing amount of evidence that strains of *P. freudenreichii* and other dairy propionibacteria have probiotic properties (reviewed recently [17]). In clinical studies concerning probiotic activity, *P. freudenreichii* strains have mainly been used as components of complex bacterial mixtures and rarely as monocultures [17]. The first step required for a probiotic to interact with a host and produce any particular response is adhesion to mucus bound to gastrointestinal epithelia [18]. While *P. freudenreichii* strains have revealed only weak and nonspecific adhesion to the mucus, the adhesion was increased by the presence of other probiotic bacteria [19, 20].

Despite the recognized role of *P. freudenreichii* in the food industry, its capability to produce appreciable amounts of active vitamin B12 and short-chain fatty acids as well as its well-documented probiotic potential, the bacterium remained poorly characterised on the genetic and genomic level. The first genome sequence was announced only in 2010 [21] shedding light on the crucial characteristics of *P. freudenreichii* such as its unique metabolism, its hardiness and probiotic potential. In addition, some misconceptions about the species have been brought to light, for example the presence of all the genes necessary for aerobic respiration led to questioning of the anaerobic status of the species. Also, it was discovered that the features used for subdivision of the species into subspecies *shermanii* and *freudenreichii*, namely lactose utilisation and nitroreductase activity, result from acquisition through horizontal gene transfer and loss due to a frameshift, respectively. This led to questioning the validity of the subdivision [21], which was proven not warranted [22]. Sequencing projects resulted in 22 draft genomes [23, 24] and two additional complete genomes [25, 26] available for the species. Although the draft genomes proved valuable and were used in a number of comparative and functional studies [24, 27–29], they do not permit studies of genome organization or mobile elements absent from the reference genome [30]. In addition, due to the nature of the short-read sequencing itself, draft genomes do not give an insight into additional regions of sequences rich in repeats such as CRISPR-Cas systems, transposed mobile elements or gene duplications [31].

Here, we report complete genome sequences of 17 additional *P. freudenreichii* strains and a re-sequenced whole genome of the strain DSM 4902. Additionally, we performed a comparative genomics study of the 20 whole genomes available to date and, owing to the long sequence reads produced by the PacBio platform, we identified several thus far unknown features of these bacteria. We report the highly variable genome organization of the strains sharing high level of sequence identity, in addition to two putative conjugative plasmids and three active temperate phages discovered as circular molecules. Genome data mining revealed complete CRISPR-Cas systems, novel restriction-modification systems, complete pili operons, the presence of putative Integrative and Conjugative Elements (ICEs) and the active transposable elements, potentially playing an important role in species adaptation.

## Results

Among the studied strains were the 14 strains from the collection of dairy company Valio Ltd., four isolated from barley grains by the malting company Polttimo Ltd., and two type strains originating from Swiss cheese (Table 1). Eighteen of the strains were sequenced with a PacBio RSII instrument, followed by assembly using Hierarchical Genome Assembly Process (HGAP3) in SMRT Analysis software (Table 2). The two remaining strains: the type strain JS16 (DSM 20271, CP010341) and JS (LN997841) were published before [25, 26]. The other type strain, JS15 (DSM 4902), has been sequenced previously [21], but it was re-sequenced with PacBio for this study.

**Table 1:** *P. freudenreichii* strains included in this study. Summary of the genome sequences

Strain	Sequence name	Accession number	Genome size (bp)	Genome coverage	GC%	No. of predicted genes	Note
JS	PFREUDJS001	LN997841	2,675,045	691	67%	2382	Sequenced previously [26]; 261 ^a^
JS2	PFRJS2	LT576032	2,655,351	317	67%	2310	257 ^a^
JS4	PFRJS4	LT576033	2,654,663	158	67%	2366	259 ^a^
JS7	PFRJS7–1	LT618776	2,738,418	300	67%	2412	Genome with prophage; 263 ^a^
	PFRJS7–2	LT618777	2,700,482	50	67%	2355	Genome without phage
	PFRJS7-ph	LT618778	37,936	60	65%	59	Circular phage
JS8	PFRJS8	LT576042	2,655,373	392	67%	2324	264 ^a^
JS9	PFRJS9–1	LT618785	2,720,049	219	67%	2405	Additional transposase gene; 265 ^a^
	PFRJS9–2	LT618786	2,718,592	219	67%	2401	
JS10	PFRJS10	LT576035	2,626,110	208	67%	2329	266 ^a^
JS11	PFRJS11	LT576038	2,537,402	501	67%	2200	274 ^a^
JS12	PFRJS12–1	LT604998	2,615,181	250	67%	2275	Additional transposase gene; 275 ^a^
	PFRJS12–2	LT576787	2,613,734	250	67%	2274	
	PFRJS12–3	LT604882	24,909	400	64%	32	Putative conjugative plasmid
JS13	PFRJS13–1	LT618779	2,537,370	210	67%	2201	11-gene insertion; 276 ^a^
	PFRJS13–2	LT618780	2,520,651	90	67%	2189	
JS14	PFRJS14	LT593929	2,507,188	330	67%	2180	277 ^a^
JS15	PFRJS15–1	LT618787	2,621,081	94	67%	2322	3 additional transposase genes; 281 ^a^
	PFRJS15–2	LT618788	2,616,005	94	67%	2320	Transposase gene disrupting a Type III RM methylase
JS16	RM25	CP010341	2,649,163	190	67%	2321	Sequenced previously [25]; 282 ^a^
JS17	PFRJS17–1	LT618789	2,755,516	192	67%	2455	Duplicated transposase gene; 283 ^a^
	PFRJS17–2	LT618790	2,754,069	192	67%	2454	
JS18	PFRJS18	LT576034	2,661,974	190	67%	2358	284 ^a^
JS20	PFRJS20–1	LT618791	2,678,207	106	67%	2384	286 ^a^
	PFRJS20–2	LT618792	2,682,327	106	67%	2376	3 additional transposase genes; one disrupting another transposase gene
JS21	PFRJS21–1	LT618781	2,659,993	222	67%	2330	Additional transposase genes; 287 ^a^
	PFRJS21–2	LT618782	2,658,550	222	67%	2329	
JS22	PFRJS22–1	LT599498	2,633,661	190	67%	2326	Genome with prophage; 288 ^a^
	PFRJS22-ph	LT615138	39,309	102	66%	61	Circular phage
JS23	PFRJS-23	LT618793	2,630,698	210	67%	2335	Genome with prophage; 289 ^a^
	PFRJS-23-ph	LT618794	42,723	25	65%	66	Circular phage
JS25	PFRJS25–1	LT618783	2,666,517	400	67%	2336	291 ^a^
	PFRJS25–2 pl	LT618784	35,640	800	64%	46	Putative conjugative plasmid

^a^ Strain number in the previous study [48]

**Table 2:** Sequencing summary

Sample Name	Sequencing Chemistry	SMRTcells	Movie Time (min) ^a^	Total Number of Subreads	Total Number of Bases	Mean Subread Length (bp)	N50 Subread Length (bp) ^b^
JS4	P4/C2	2	120	142,306	465,129,578	3268	4131
JS10	P4/C2	2	120	205,113	606,802,054	2958	3733
JS15	P4/C2	2	120	179,642	540,206,310	3007	3653
JS18	P4/C2	2	120	194,246	550,199,736	2832	3679
JS20	P4/C2	2	120	230,371	632,635,668	2746	3456
JS22	P4/C2	2	120	181,361	563,541,807	4090	3107
JS23	P4/C2	2	120	192,373	617,310,336	3208	4222
JS2	P5/C3	2	240	100,864	719,516,424	7133	8940
JS7	P5/C3	2	240	107,194	1,002,978,417	9356	13,043
JS8	P5/C3	2	240	139,418	1,149,552,255	8245	11,148
JS9	P5/C3	2	240	160,808	1,316,084,462	8184	11,023
JS11	P5/C3	2	240	184,744	1,422,323,823	7698	10,498
JS12	P5/C3	2	240	207,391	1,490,421,212	7186	9765
JS13	P5/C3	2	240	103,466	793,823,369	7672	10,156
JS14	P5/C3	2	240	125,130	966,521,587	7724	10,326
JS17	P5/C3	2	240	151,002	1,149,295,071	7611	10,232
JS21	P5/C3	2	240	173,843	1,298,367,718	7468	9906
JS25	P5/C3	2	240	192,104	1,293,994,542	6735	8805

^a^ run time during which nucleotides added by the polymerase are recorded in real-time

^b^ minimum length of subreads in which half of the sequencing data is found

We assembled 31 complete and circular sequences from the eighteen strains. For 11 of the strains, assembly resulted in more than one genome. In five of the strains circular elements were found: in JS12 and JS25 putative conjugative plasmids and in JS7, JS22 and JS23 bacteriophage genomes. In eight of the strains, the additional genomes resulted from duplication and relocation (copy and paste) of transposable elements (Table 3).

**Table 3:** *P. freudenreichii* strains included in this study. The details of differences between the genome sequences within strains

Strain	Accession number	GC%	Note	Details
JS7	LT618776	67%	Genome with prophage	PFR_JS7–1_1810 HTH-type transcriptional regulator KmtR:PFR_JS7–1_1869 Transcriptional regulator MtrR (preceeded by tRNA-Lys)
	LT618777	67%	Genome without phage	
JS9	LT618785	67%	Genome with an additional transposase gene	PFR_JS9–1_62 Uma4 protein
	LT618786	67%		
JS12	LT604998	67%	Genome with an additional transposase gene	PFR_JS12–1_615 ^a^ Transposase of ISAar20, ISL3 family and PFR_JS12–1_616 Hypothetical protein (ahead of Carbon starvation protein)
	LT576787	67%		
JS13	LT618779	67%	Genome with 11-gene insertion or deletion ^b^	JS13_289 Hypothetical protein: JS13_299 Transposase for insertion sequence element IS1001
	LT618780	67%		
JS15	LT618787	67%	3 transposase genes absent from the other genome	PFR_JS15–1_878 Uma4 protein and PFR_JS15–1_879 Hypothetical protein; PFR_JS15–1_1737 Transposase of ISAar20, ISL3 family (Transposase gene disrupting gene coding for CitT); PFR_JS15–1_2045 ^a^ Transposase of ISAar20, ISL3 family
	LT618788	67%	Transposase gene disrupting a Type III restriction enzyme	PFR_JS15–2_359 Transposase of ISAar43,IS3 family, IS407 group, orfA and PFR_JS15–2_360 ^c^ Insertion sequence IS407 orfB
JS17	LT618789	67%	A duplicated transposase gene	PFR_JS17–1_657 and PFR_JS17–1_658 ^a^ Transposase of ISAar20, ISL3 family
	LT618790	67%		
JS20	LT618792	67%	3 transposase genes absent from the other genome	PFR_JS20–2_544 Insertion sequence IS407 OrfB and PFR_JS20–2_545 ^c^ Transposase of ISAar43,IS3 family, IS407 group, orfA (disrupting Transposase IS30 family gene); PFR_JS20–2_568 Transposase of ISAar20, ISL3 family (ahead of Carbon starvation protein); PFR_JS20–2_1234 Transposase of ISAar20, ISL3 family (ahead of LuxS)
	LT618791	67%		
JS21	LT618781	67%	Transposase gene ahead of putative aminotransferase biotin synthesis related protein	PFR_JS21–2_248 Transposase of ISAar20, ISL3 family
	LT618782	67%		

^a^ Transposase genes PFR_JS12–1_615, PFR_JS15–1_2045, PFR_JS17–1_657 and PFR_JS17–1_658 found in strains JS12, JS15 and JS17, respectively, are 100% identical

^b^ The same element is present in the strain JS11 in the same location, but in all of the sequences

^c^ Transposase genes PFR_JS15–2_359 and PFR_JS15–2_360 are identical to PFR_JS20–2_544 and PFR_JS20–2_545. The transposase genes do not appear to be acquired through horisontal transfer, as each of them is present in their respective genomes in at least one more location. All of the transposase genes have counterparts in multiple strains, suggesting their intrinsic character for the species

### Genome organization

**Fig. 1:**
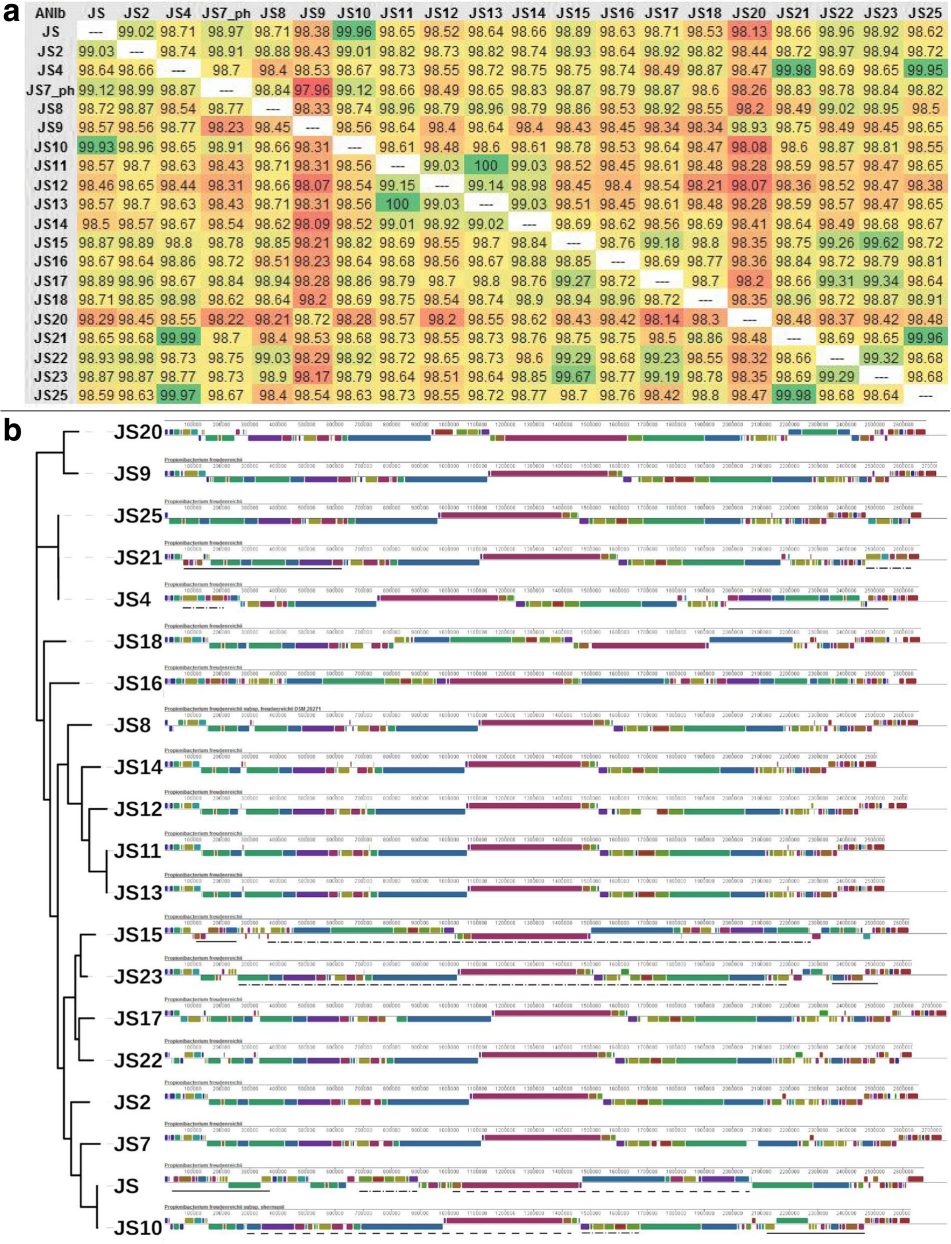
Genome composition and organisation. Panel **a**) Average Nucleotide Identity (%) calculated based on pairwise BLAST alignment (ANIb). The levels of similarity are highlighted by coloring from green for the most similar to red for the most dissimilar. The strains JS4, JS15 and JS17 are on average the most similar to all other strains, while the strains JS9 and JS20 are the most dissimilar to all other strains and only slightly more similar to one another. The strains of cereal origin (JS11-JS14) are more similar to each other than to other strains. Panel **b**) Whole genome alignments generated with ProgressiveMauve. The genomes are arranged according to the phylogenetic tree generated from core genome alignments (see below). The distinct organisation of the genomes of closely related strains can be observed, most clearly between strains JS and JS10, JS15 and JS23 as well as JS4 and JS21. The regions of genome rearrangements in these strains are indicated with matching lines (solid, dot-dash or dash)

Average Nucleotide Identity (ANI) calculated through pairwise BLAST alignments revealed that genomes of the *P. freudenreichii* strains are highly collinear, with an ANI value of nearly 99% on average (Fig. 1a). The whole genome alignments show that despite genome-wide collinearity, large regions of inversions and other types of re-organisations are present even among the most closely related strains (Fig. 1b).

In eight of the sequenced strains we observed translocation of mobile elements, either with transposase genes alone or, in strain JS13, as a part of larger gene cluster. The gene cluster consists of 12 coding sequences: four transposase genes and eight hypothetical proteins, one with similarity to “Helicase conserved C-terminal domain” (PF00271.25). Additionally, we observed a transposase-mediated duplication in strain JS17, which was confirmed by PCR to eliminate the possibility of an assembly error. The duplication spans 35 genes: PFR_JS17–1_676-PFR_JS17–1_710 and PFR_JS17–1_711-PFR_JS17–1_745, located between genes coding for an Uma4 type transposase and an aspartate ammonia lyase. The duplication region included genes coding for, among others, thiamine biosynthetic proteins, transporters and glycerol metabolism.

### Comparative genomics

**Fig. 2:**
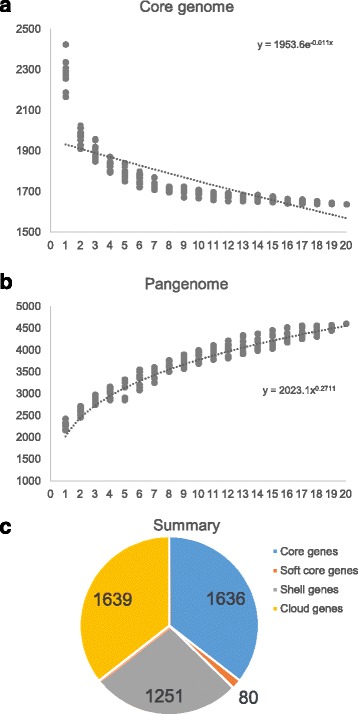
Core genome and pan genome of the *P. freudenreichii* species. The core genome (**a**) and the pan genome (**b**) are represented as a variation of the gene pools after sequential addition of 20 *P. freudenreichii* genomes. The summary of ortholog group distribution between the strains is presented in a pie chart (**c**). Core genes- present in all of the strains; Soft core genes- present in 19 of the strains; Shell genes- present in 3-18 of the strains; Cloud genes- present in one or two strains only

The pangenome of the 20 *P. freudenreichii* strains was analysed with Roary [32] revealing 4606 ortholog groups. The core genome, defined as ortholog groups found in all of the isolates, consisted of 1636 orthologs. The soft core, ortholog groups found in 19 out of 20 isolates, consisted of 80 additional orthologs, while the 1251 ortholog qroups found in three to 18 strains made up the shell genome. The remaining 1639 ortholog groups were assigned to the cloud genome consisting of the ortholog groups which were found in either one or two strains only (Fig. 2).

**Fig. 3:**
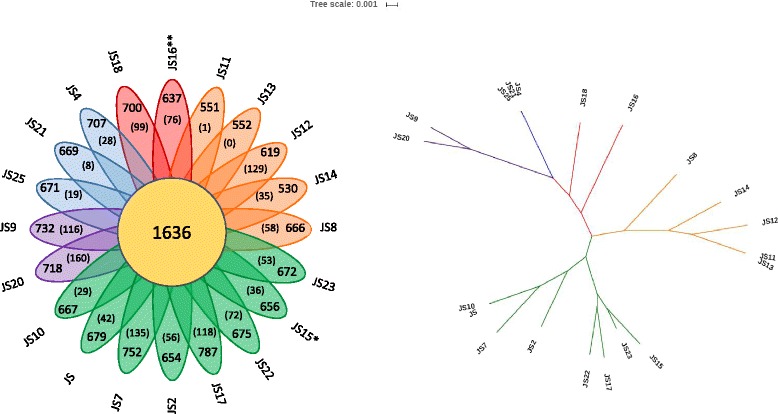
Flower plot representing comparative analysis of the genome. The orthologous groups shared between the strains are indicated in the center. The number of accessory genes for each strain are indicated on each petal. In the brackets are the genes unique to that strain. The petals are colored based on the degree of relatedness of the strains. The unrooted phylogenetic tree was created based on the core genome alignments. *Type strain *P. freudenreichii* DSM 4902; **Type strain *P. freudenreichii* DSM 20271 (CP010341)

**Fig. 4:**
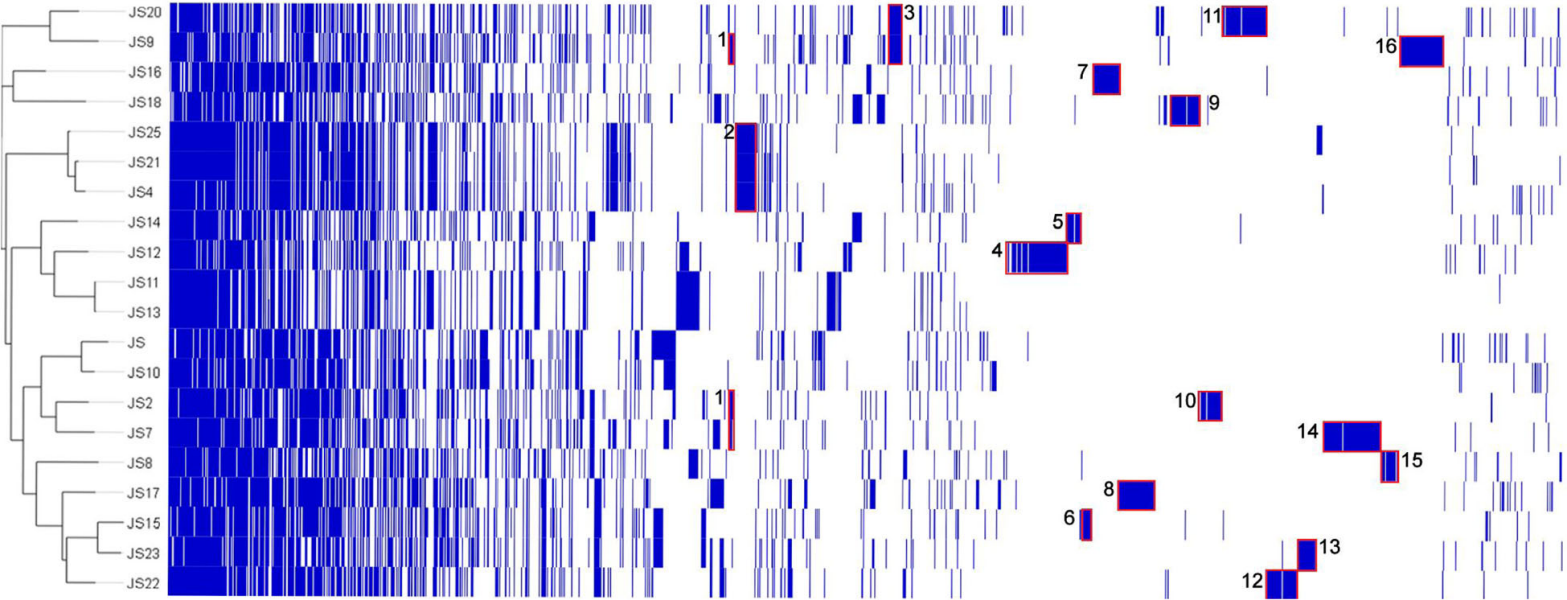
Map of accessory genome alignments generated by Roary. Gene clusters unique to individual strains are marked in red and numbered. 1) Genomic island with genes coding for the CASCADE-like CRISPR-Cas systems in strains JS2, JS7 and JS9; 2) Genomic island unique to strains JS4, JS21 and JS25. Genes located on this island include transposase genes with 96–98% sequence identity to those from *Corynebacterium urealyticum* DSM 7111 and a gene coding for an additional Cobyrinic acid A,C-diamide synthase; 3) Heat shock island unique to strains JS9 and JS20. Genes on the island include an 18-kDa heat shock protein, DnaK, GrpE, CbpM, ClpB and others; Features 4–16 are gene clusters unique to their respective strains. These include complete prophages (8, 12, 13, and 14), phage remnants (6, 8, 9 and 12), predicted genomic islands with genes encoding various functions: resistance to heavy metals (7), possible antibiotic resistance (15), genetic loci with genes coding for restriction and modification systems (7, 11, 12 and 14), and the pilus locus (9). Unique gene clusters 4, 9, 11, 14 and 16, despite sequence differences share structural similarities, including the presence of genes coding for Single-stranded DNA-binding protein, TraM recognition site of TraD and TraG, AAA-like domain protein (VirB4-like), Multifunctional conjugation protein TraI (TrwC or TraA relaxase), Type IV secretory system Conjugative DNA transfer (TraG-like), ParB-like nuclease domain protein, Bifunctional DNA primase/polymerase and Murein DD-endopeptidase MepM. The presence of TraA, TraG and VIrB4 are indicative of Integrative and Conjugative Elements (ICEs) type T4SS. The majority of the unique gene clusters have regions with a high degree of sequence identity to other Actinobacteria, including *Propionibacterium acidipropionici, Corynebacterium falsenii, Cutibacterium avidum* and *Microbacterium sp*. Details can be viewed in the Additional tables of the respective strains in the column “Note”

The numbers of accessory genes in individual strains and numbers of unique genes varied between genomes (Fig. 3). To better visualise the differences between genomes a presence-absence matrix was created from the orthologs assigned to accessory genome (Fig. 4). The strains are organised into a phylogenetic tree based on the accessory genome alignments. The unique gene clusters accounting for the most obvious differences between genomes are highlighted (more detailed results in Additional file 1). The core genome size needs to be addressed with caution, as out of 1636 genes 457 differed in predicted size among the strains, 200 of which differed by a minimum of 90 nucleotides (see Additional file 1). The frequent co-localisation of such genes with the genes coding for short hypothetical proteins may be indicative of evolutionary events, which resulted in splitting of the coding sequence, mis-annotation or sequencing errors.

**Fig. 5:**
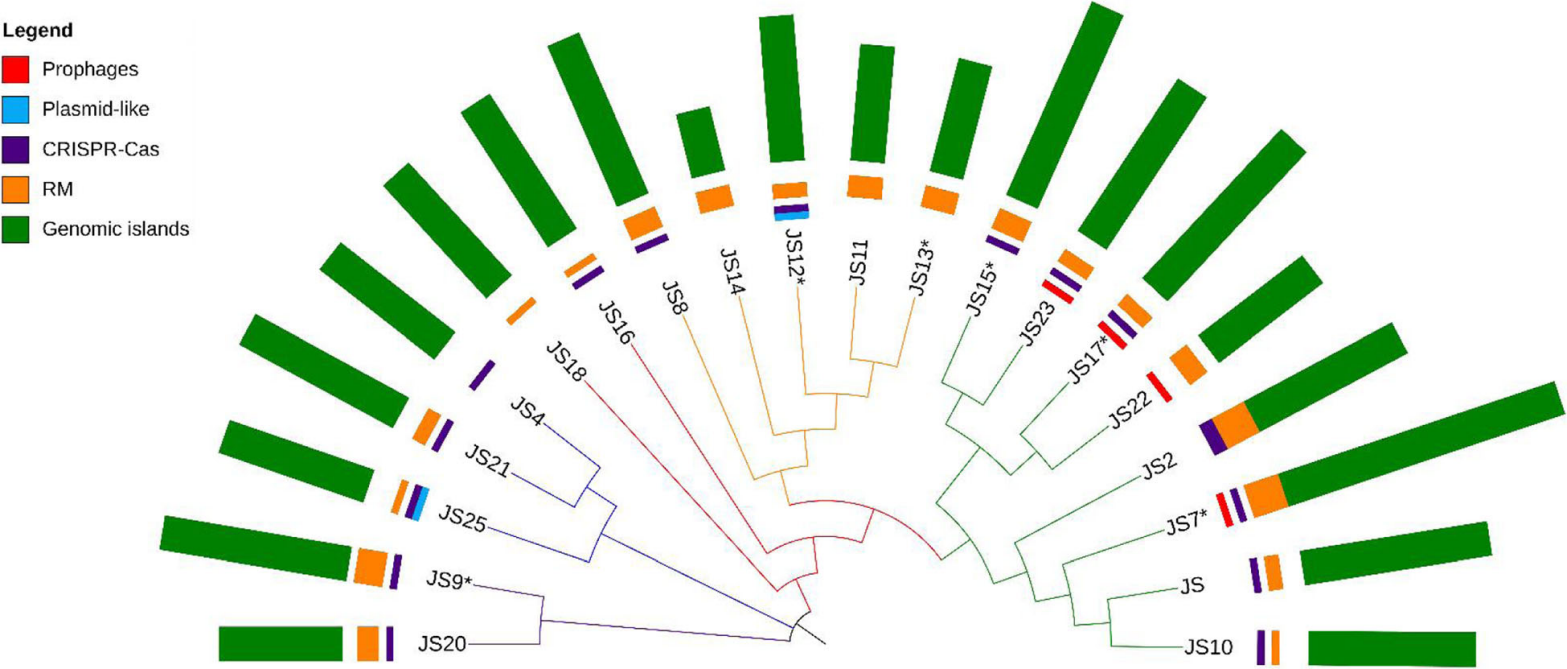
Summary of the genomic features. Core genome alignment phylogenetic tree with the genomic features displayed on a multibar chart, including detected prophages, plasmid-like elements, complete CRISPR-Cas systems, RM systems for which methylases were unambiguously matched with recognition sequences and the genomic islands predicted by at least one method. The strains for which more than one version of the genome was detected are marked with an asterisk

To characterise the individual genomes further, bioinformatics analyses were performed, including searches for prophages, genomic islands, CRISPR-Cas systems and restriction- modification (RM) systems. The cumulative results are summarised in Fig. 5 and the details can be viewed in Additional files 2, 3, 4, 5, 6, 7, 8, 9, 10, 11, 12, 13, 14, 15, 16, 17, 18, 19, 20 and 21.

### Mobile elements

#### Bacteriophages

**Fig. 6:**
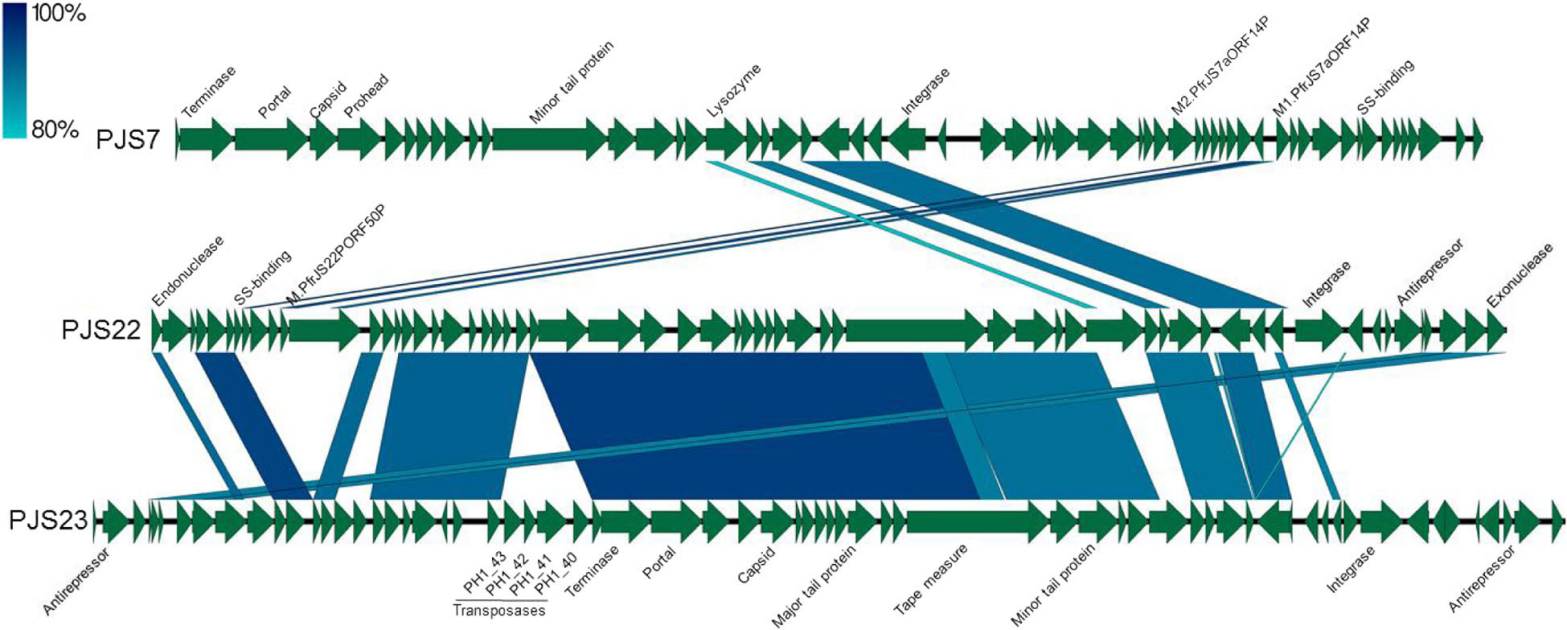
Bacteriophages identified in this study. PJS7 is 99% identical with the recently published genome of Propionibacterium phage PFR1 (NC_031076.1), but different from the other phages identified in this study. Phages PJS22 and PJS23 are similar to each other and to closely related Propionibacterium phages B22 (KX620750.1) and Doucette (KX620751.1). Part of the annotations was derived from the most closely related phages and can be viewed in Additional Tables JS7, JS22 and JS23

In this study, three bacteriophages were discovered as a circular DNA within the strains JS7, JS22 and JS23 (Fig. 6). The phage found in the strain JS7 (LT618778) has a total genome size of 37,936 bp and 59 predicted open reading frames. When integrated into the chromosome as a prophage, it was located between the sequence coding for the transcriptional regulator KmtR, immediately downstream of a tRNA-Ala (agc) and a tRNA-Lys(ttt), immediately upstream of a transcriptional regulator MtrR. A BLAST search against the known Propionibacteria phages showed that PJS7 is 99% identical with the 38,071 bp-long PFR1 phage (NC_031076.1). The difference can be found in the gene coding for the minor tail protein, where PFR-JS7_47 is 135 nucleotides shorter than BI042_gp13. The strain JS7 differed from the other phage-carrying strains, since sequencing revealed the coexistence of three types of genomes in its DNA-sample: a bacterial genome carrying a prophage (LT618776), a circular phage genome (LT618778), and unlike in samples of JS22 or JS23, also a bacterial genome cleared of prophage (LT618777). Replication of the circular phage genome in JS7 was followed by PCR after sub-culturing, which revealed successive integration of phage genomes after five passages in the PPA medium (Additional file 5: Phage integration). The other two strains, JS22 and JS23 carried the prophage on all the copies of the chromosome as well as the circular phages. The phages PJS22 and PJS23 are 97% identical over 68% of their sequences. PJS22 shows 99% identity over 81% sequence to previously sequenced phage B22 (KX620750.1), PJS23 is the most similar to the phage Doucette (KX620751.1) with 97% identity over 64% of the sequence. The PJS22 phage is inserted between sequence coding for tRNA-Gly (ccc) and a DNA protection during starvation protein 2 (PFR_JS22–1_1997) while the PJS23 prophage is inserted between tRNA-Pro(tgg) and a Quaternary ammonium compound-resistance protein SugE (PFR_JS23_1469).

All the genomes were checked for additional prophage sequences with two dedicated programs: Phaster and Prophinder. From the candidate prophages, only the prophage from the strain JS17 appeared complete. The prophage JS17 is located between the tRNA-Ser(tga) gene and a transposase gene (PFR_JS17–1_2095). A BLAST search revealed 96% identity over 61% and 64% of the sequence to Propionibacterium phages Doucette and G4, respectively. Similarly, a BLAST analysis against phages PJS22 and PJS23 showed 97% identity over 62% and 65% of the sequence, respectively.

In the sequences of the prophage of the strain JS17 and the bacteriophage PJS23 a number of transposase genes were found. The PFR_JS17–1_2038 was identical to eight (PFR_JS17–1_341, PFR_JS17–1_394, PFR_JS17–1_676, PFR_JS17–1_711, PFR_JS17–1_2205, PFR_JS17–1_2341, PFR_JS17–1_2347 and PFR_JS17–1_2416), while PFR_JS17–1_2067 was identical to six (PFR_JS17–1_13, PFR_JS17–1_46, PFR_JS17–1_72, PFR_JS17–1_657, PFR_JS17–1_658 and PFR_JS17–1_1466) transposase genes found in other locations in the same strain. In addition, the PFR_JS17–1_657 and PFR_JS17–1_658 were the ones observed as duplicated in only part of the genome sequences of the strain JS17. These transposase genes were identical to the ones found in only part of the genome sequences of the strains JS12 and JS15 (see Table 3). Within prophage PJS23 sequence, there were four transposon-like elements, PFR_JS23_1432-PFR_JS23_1435 (PH1_40-PH1_43 on the phage genome). The PFR_JS23_1432 and PFR_JS23_1435 were both unique to the phage region of the genome, while PFR_JS23_1433 (integrase) and PFR_JS23_1434 (transposase) were each found in two additional, co-located copies on the bacterial chromosome (PFR_JS23_368 and PFR_JS23_369; PFR_JS23_2185 and PFR_JS23_2184).

#### Plasmid-like elements

Two plasmid-like elements PFRJS12–3 (LT604882) and PFRJS25–1 (LT618784) were detected from strains JS12 and JS25, respectively. PFRJS12–3 and PFRJS25–1 are of 24.9 kbp and 35.6 kbp in size and include 32 and 46 predicted open reading frames, respectively (Additional file 10: LT604882 (plasmid) and Additional file 21: LT618784 (plasmid)). According to homology searches PFRJS12–3 and PFRJS25–1 sequences share no significant similarity with reported *P. freudenreichii* plasmids. In addition, no similarity to plasmids pIMPLE-HL096PA1 [33] or PA_15_1_R1 from the closely related species, *Cutibacterium acnes,* was found. A BLASTn search of the PFRJS12–3 revealed that the gene PFR_JS12–3_15 encoding a transposase is 93–95% identical with the transposase genes of *P. freudenreichii*, *Acidipropionibacterium acidipropionici*, *Micrococcus luteus* and *Corynebacterium variabile* at positions 8594–9669. The transposase gene PFR_JS12–3_12 in PFRJS12–3 is 90% identical to *A. acidipropionici* and *Micrococcus luteus* sequences at position 5799–7130, and the gene PFR_JS12–3_22is 92% identical to a resolvase gene from *A. acidipropionici* at positions 12,361–12,930. BLASTn search of PFRJS25–1 revealed a stretch of 88% identity to the *Propionibacterium* phage PFR1 over the stretch of its genes PFR1_23, PFR1_24 and PFR1_25, all encoding hypothetical proteins. Additionally, the 5′ end of this sequence showed 98% identity with a 47 nt stretch in the non-coding region in *Burkholderia pyrrocinia* plasmid p2327 and *Burkholderia cenocepacia* plasmid pBCJ2315. A BLASTp search using the predicted proteins of PFRJS25–1 against those from p2327 and pBCJ2315 revealed negligible sequence similarity.

**Fig. 7:**
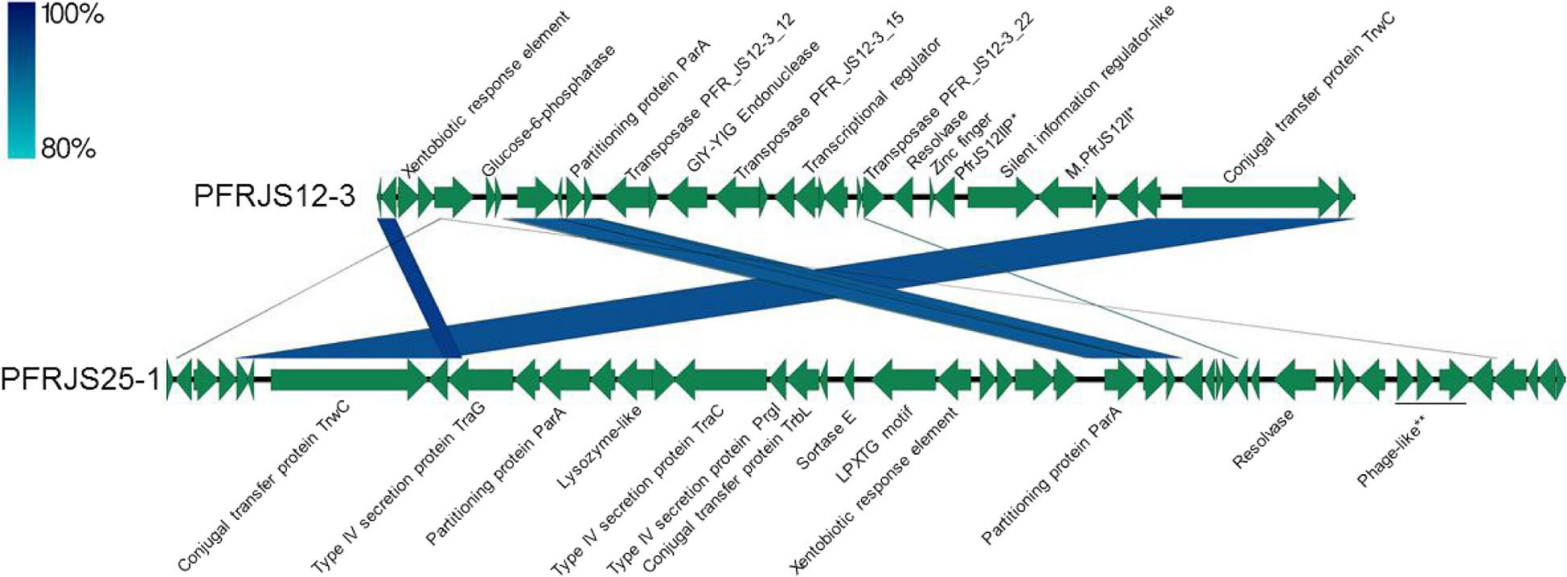
Putative conjugative plasmids identified in this study. *Type II restriction-modification system with the recognition motif CTCGAG. **DNA stretch with 88% nucleotide identity to Propionibacterium phages PFR1 (NC_031076.1), PFR2 (KU984980.1) and G4 (KX620754.1)

Further analysis showed that the PFRJS25–1 was 99% identical over 31% of the sequence of PFRJS12–3 (Fig. 7). The analysis comparing sequences against the Conserved Domains Database (CDD) [34] revealed multiple regions of similarity to conjugative plasmids from both elements. These regions of similarity included those with conserved domains of conjugal transfer proteins TrwC, TraC, TraG and TrbL as well as plasmid partition protein ParA. As no characteristic replication origin loci were found it remains to be elucidated whether the circular elements found in strains JS12 and JS25 are plasmids.

#### Genomic islands

The genomes were assessed for the presence of genomic islands by the integrative online tool IslandViewer 3 [35], which performs the analysis with three independent genomic island prediction methods: IslandPick, IslandPath-DIMOB, and SIGI-HMM.

The ability to utilise lactose, a historically important trait in *P. freudenreichii*, has been previously tied to a genomic island on which genes coding for UDP-glucose 4-epimerase (*galE1*), Sodium:galactoside symporter (*galP*) and Beta-galactosidase (*lacZ*) are located [21]. In our study, besides the type strain JS15, the same island was found in nine other strains: JS, JS2, JS7, JS8, JS10, JS17, JS18, JS22 and JS23, with JS23 possessing two copies of the region (PFR_JS23_160-PFR_JS23_162 and PFR_JS23_2069 -PFR_JS23_2071) (Additional files 2, 3, 4, 5, 6, 7, 8, 9, 10, 11, 12, 13, 14, 15, 16, 17, 18, 19, 20 and 21). The presence of the island correlates with the ability to utilise lactose by these strains in vivo (see Additional file 22).

Another feature potentially giving a competitive advantage in the dairy environment is the ability to degrade D-lactate. Eight strains, including JS2, JS7, JS8, JS10, JS15, JS17, JS20 and JS23 were found to be equipped with a D-lactate dehydrogenase encoding gene located on a genomic island, while the gene encoding D-lactate dehydrogenase in strain JS18 is located just downstream of a predicted genomic island. For other traits important in food production see Additional file 23.

In strain JS4, a genomic island with an alternative pathway for the biosynthesis of rhamnose consisting of genes for dTDP-4-dehydrorhamnose reductase (*rmlD*), a putative dTDP-4-dehydrorhamnose 3, 5-epimerase (*rfbC*) and dTDP-glucose 4, 6-dehydratase (*rmlB*) was found. Finally, an island on which genes coding for pilus components was found in strain JS18, including Sortase SrtC1 (PFR_J18_2247), Type-2 fimbrial major subunit (PFR_J18_2248) and a Surface-anchored fimbrial subunit (PFR_J18_2249) (Additional file 16).

*P. freudenreichii* were previously reported to possess anti-inflammatory properties [29, 36]. Those properties were associated with a range of S-layer proteins: SlpE [29], SlpA and SlpB [36]. Genes coding for SlpA (RM25_1747 in the reference strain) and another Slp protein (RM25_1746) were found in all of the strains, in seven of the strains (JS, JS2, JS4, JS10, JS17, JS18 and JS23) identified as a part of a genomic island. The complete genes coding for the SlpE protein (Hypothetical protein) were found in 12 of the strains included in this study (PFR_JS2_13, PFR_JS4_13, PFR_JS8_26, PFR_JS9–1_12, PFR_JS10_12, PFR_JS12–1_12, PFR_JS14_12, PFR_JS15–1_12, PFR_JS17–1_12, PFR_JS22–1_12, PFR_JS23_12, PFR_JS25–1_2272); SlpB was found in two strains (PFR_JS14_229 and PFR_JS17–1_279). In addition, a 220 aa long S-layer protein precursor (encoded by the *ctc* gene) was found in 13 of the strains (PFREUDJS001_001526, PFR_JS2_711, PFR_JS8_732, PFR_JS10_644, PFR_JS11_672, PFR_JS12–1_664, PFR_JS13–1_672, PFR_JS14_687, RM25_1523, PFR_JS17–1_772, PFR_JS18_1933, PFR_JS22–1_727, PFR_JS23_662).

We surveyed the genomes for known antibiotic resistance genes. In nearly all of the *P. freudenreichii* strains these genes are not located on a predicted genomic island, however, strain JS8 appears to be diverging from the group. The genomic island unique to strain JS8 (see Fig. 4, feature 15) includes three genes coding for putative antibiotic resistance-related proteins: mitomycin radical oxidase, tetracyclin repressor domain-containing protein and a puromycin resistance protein Pur8. Furthermore, the edges of the island are flanked by the genes coding for hypothetical proteins which have 98 and 99% sequence identity to genes from *Brevibacterium linens* strain SMQ-1335 coding for mobile element proteins (see Additional file 6).

### Immunity

#### CRISPR-Cas systems

**Fig. 8:**
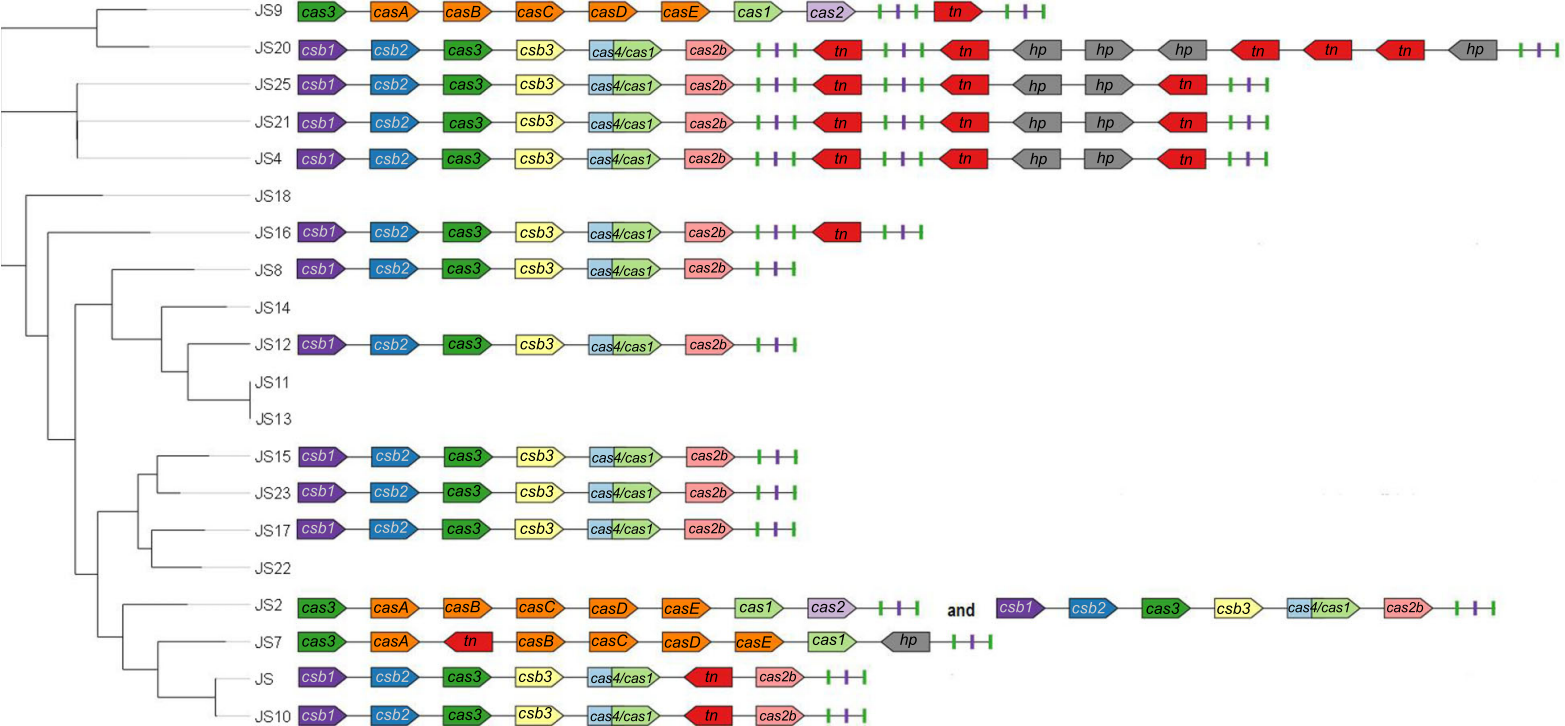
CRISPR-Cas systems detected in the sequenced strains. Strains JS9, JS2 and JS7 possess CRISPR-Cas sytem type IE (CASCADE), while all the other strains possess system type IU. Only strain JS2 possessess both types of the CRISPR-Cas systems. Green-purple-green boxes indicate presence of repeats and spacers. tn- transposase; hp.- hypothetical proteins

The clustered regularly interspaced short palindromic repeats (CRISPR) together with CRISPR associated proteins (Cas) form the adaptive immunity systems protecting their hosts against invasion by foreign DNA. The function of the adaptive immunity system can be divided into two phenomena: CRISPR adaptation and CRISPR interference. The CRISPR adaptation results from spacer acquisition upon exposure to invading DNA, while the CRISPR interference involves recognition of the specific spacers on foreign DNA, which in turn allows for introduction of breaks in of the invading DNA and its resulting destruction reviewed by Savitskaya [37]. Currently, the CRISPR-Cas systems are subdivided into two classes, five types and 16 subtypes. Following this classification, we identified two systems in *P. freudenreichii* which, based on the presence of the Cas3 protein, we classified as belonging to the class1, type I CRISPR systems [37] (Fig. 8). The first of the systems, with the direct repeat consensus GGATCACCCCCGCGTATGCGGGGAGAAC, may be classified as the subtype IE based on sequence homology and gene organisation corresponding to the CASCADE system, which is well-characterised in *E. coli* [38, 39]. The second system, with the direct repeat consensus ATTGCCCCTCCTTCTGGAGGGGCCCTTCATTGAGGC, bears similarity to the subtype IU (previously IC) [38], which is strengthened by the presence of the fusion protein Cas4/Cas1 found in several variants of the subtype IU [40, 41]. However, the atypical gene organisation suggests that it is a new variant of the subtype IU.

The CRISPR system IE was found in strains JS2, JS9 and JS7 and carried 96, 65 and 105 spacers, respectively (Table 4). These systems were located on genomic islands in all strains, which suggested relatively recent acquisition; however, the lack of sequence identity between the spacers suggested independent acquisition of immunity in each strain. The CRISPR system of strain JS7 had a transposase gene inserted between the *cse1* and *cse2* genes and only a fragment of the *cas2* gene comprising a part of a larger hypothetical protein. In strain JS9 the first 9 CRISPR spacers were separated from the following 96 spacers by an integrase. BLAST search of the spacers indicated immunity to all of the previously sequenced phages infecting *P. freudenreichii*, apart from the filamentous phage phiB5, for which immunity was found only in strain JS9. In addition, strain JS2 carried immunity against all three phages found in this study, and to the plasmid pJS25. The strain JS7 carried immunity to the plasmid pJS12, the phage PJS22 and to the phage by which it is infected (PJS7), suggesting that the presence of either the transposase gene or the incomplete *cas2* gene may have resulted in inactivity of the CRISPR-Cas system in this strain. JS9 carried markers of immunity against all the three phages found in this study and to the plasmid-like element PFRJS25–1.

**Table 4:** CRISPR-Cas systems identified in the studied strains by CRISPR-finder

Strain	Predicted CRISPR	Start	End	Size	DR (nt)	No. spacers	Type	Immunity to identified phages	Immunity to mobile elements identified in this study	Locus ID	Note
JS	Crispr_2	2,424,099	2,427,021	2922	36	40	IU	Anatole, B3, B22, E1, E6, G4, PFR1, PFR2	PJS22, PJS23, PJS7, pJS12	PFREUDJS001_002141-PFREUDJS001_002147	–
JS2	Crispr_3	2,082,550	2,088,376	5826	28	95	IE	B22, B3, E1,E6, G4, Anatole, Doucette, PFR1, PFR2	PJS22, PJS23, PJS7, pJS25	PFR_JS2_1825-PFR_JS2_1832	–
	Crispr_4	2,120,759	2,122,610	1851	36	25	IU	PFR1, PFR2	PJS7	PFR_JS2_1843-PFR_JS2_1849	–
JS4	Crispr_3	1,825,170	1,827,746	2576	36	35	IU	Doucette, E6, G4, PFR1, PFR2	PJS22, PJS7, pJS25	PFR_JS4_1650-PFR_JS4_1655	One system, negative strand, spacers separated by a transposase gene and then by a four-gene integron.
	Crispr_2	1,823,162	1,823,697	535	36	7		–	–		
	Crispr_1	1,818,598	1,819,215	617	36	8		–	–		
JS7	Crispr_2	2,203,337	2,207,335	3998	28	65	IE	B22, B3, E1,E6, G4, Anatole, Doucette, PFR1, PFR2	PJS22, PJS7, pJS12	PFR_JS7–1_1964-PFR_JS7–1_1971	No *cas2*
JS8	Crispr_3	2,192,257	2,196,491	4234	36	58	IU	Anatole, Doucette, B3, B22, E1, E6, G4, PFR1, PFR2, phiB5	PJS22, PJS23, PJS7, pJS25	PFR_JS8_1930-PFR_JS8_1935	–
JS9	Crispr_3	2,260,079	2,260,655	576	28	9	IE	–	–	PFR_JS9–1_2018-PFR_JS9–1_2025	One system, negative strand, spacers separated by an integrase
	Crispr_2	2,253,046	2,258,937	5891	28	96		B22, B3, E1,E6, G4, Anatole, Doucette, PFR1, PFR2, phiB5	–		
	Crispr_4	2,288,755	2,292,746	3991	29	65	N/A	Doucette, B3, B22, G4, PFR1, PFR2	PJS23, PJS7		Remnant of a system, no Cas. Spacers separated by an integrase.
	Crispr_5	2,293,887	2,295,016	1129	29	18		PFR1, PFR2	PJS7		
JS10	Crispr_2	2,029,533	2,032,236	2703	36	37	IU	Anatole, B3, B22, E1, E6, G4, PFR1, PFR2	PJS22, PJS23, PJS7, pJS12	PFR_JS10_1797-PFR_JS10_1803	
JS12	Crispr_1	2,181,462	2,185,411	3949	36	54	IU	Doucette, B3, B22, E6, G4, PFR1, PFR2, phiB5	PJS22, PJS23, PJS7	PFR_JS12–1_1906-PFR_JS12–1_1911	
JS14	PossibleCrispr_1	1,595,184	1,595,290	106	36	1	N/A	Anatole, E1		PFR_JS14_1397	
JS15_1	Crispr_1	441,841	444,266	2425	36	33	IU	Anatole, B3, B22, E1, E6, G4, phiB5	PJS23, pJS12	PFR_JS15–1_371-PFR_JS15–1_376	
JS16	Crispr_2	417,820	419,522	1702	36	23	IU	B22, E6, G4, PFR1, PFR2	PJS22, PJS23, PJS7	RM25_0342-RM25_0347	One system, negative strand, spacers separated by a transposase gene. Both CRISPR stretches carry immunity to known phages.
	Crispr_1	413,342	416,347	3005	36	41		Anatole, B3, B22, E1, E6	PJS22		
JS17_1	Crispr_2	2,220,735	2,225,402	4667	36	64	IU	Anatole, Doucette, B3, B22, E1, PFR1, PFR2	PJS22, PJS23, pJS12, pJS25	PFR_JS17–1_1953-PFR_JS17–1_1958	–
JS20_1	Crispr_3	2,034,463	2,037,183	2720	36	37	IU	Doucette, B22, E6, G4, PFR1, PFR2	PJS23, PJS7	PFR_JS20–1_1815-PFR_JS20–1_1820	One system, spacers separated by a transposase gene and then by an eight-gene integron.
	Crispr_4	2,038,656	2,039,048	392	36	5		–	–		
	Crispr_5	2,044,247	2,044,936	689	36	9		–	–		
JS21_1	Crispr_5	2,189,758	2,192,624	2866	36	39	IU	Doucette, E6, G4, PFR1, PFR2	PJS22, PJS7, pJS25	PFR_JS21–1_1963-PFR_JS21–1_1968	One system, negative strand, the spacers separated by a transposase gene and then by a four-gene integron.
	Crispr_4	2,187,750	2,188,285	535	36	7		–	–		
	Crispr_3	2,183,185	2,183,803	618	36	8		–	–		
JS22	Crispr_2	2,150,779	2,151,318	539	36	7	IU	–	–		Remnant of the same IU system, but no Cas. No immunity to known phages found.
JS23	Crispr_2	2,124,520	2,126,512	1992	36	27	IU	Anatole, B3, B22, E1, E6, G4, phiB5	PJS23	PFR_JS23_1889-PFR_JS23_1894	–
JS25	Crispr_2	2,042,081	2,045,018	2937	36	40	IU	Doucette, E6, PFR1, PFR2	PJS22,PJS7,pJS25	PFR_JS25–1_1807-PFR_JS25–1_1812	One system, spacers separated by a transposase gene and then by a four-gene integron.
	Crispr_3	2,046,491	2,046,954	463	36	6		–	–		
	Crispr_4	2,050,901	2,051,519	618	36	8		–	–		

The CRISPR-Cas system IU is more widespread in *P. freudenreichii* and can be found in 13 of the sequenced strains, including only one strain of cereal origin-JS12. In strains JS4, JS16, JS20, JS21 and JS25 the systems are located on genomic islands predicted by Island Viewer. The number of spacers in IU CRISPR-Cas systems ranged from 25 in the strain JS2 to 64 in strain JS17 (Additional file 24). The spacers of the strains JS and JS10 and of the strains JS4, JS20, JS21 and JS25 are for the most part identical, which is in line with their phylogenetic relatedness. In other strains, only a few spacers are identical suggesting early diversification. Only strain JS2 carries both types of CRISPR systems, although strain JS9 possesses an additional stretch of 83 spacers separated by a distinct repeat sequence (GGGCTCACCCCCGCATATGCGGGGAGCAC), indicating they would belong to a separate CRISPR-Cas system. Nevertheless, the lack of Cas genes in the vicinity and location of the CRISPR on a genomic island may mean that the system was acquired through incomplete horizontal gene transfer.

In strains JS11, JS13, JS14, JS18 and JS22 no complete CRISPR-Cas systems were found, although strain JS22 had a short stretch of CRISPRs. No immunity to known phages was found on that stretch. For each of the strains, 2–4 additional “Possible CRISPRs” were identified, most of which mapped within sequences coding for DNA topoisomerase 1, a hypothetical protein or fell between coding sequences. None of them showed homology to known Cas genes. Still, the “Possible CRISPR1” from strain JS14 carries a spacer with 100% identity to a fragment of the gene coding for a tape measure protein in phages Anatole and E1.

Interestingly, strain JS23 appears to have an intact CRISPR-Cas system and one spacer with 100% identity to the sequence of the prophage the strain carries. This could mean that the system is not functional, that one spacer is not sufficient to destroy the phage DNA, or that the phage possesses a mechanism of countering the strategies employed by the host. We explored the possibility that the mobile elements might carry anti-CRISPR genes [42]. To this end we performed a conserved domain search [34] of the mobile elements for which self-immunity was found, namely phages PJS7, PJS23, prophage of the strain JS17 and the plasmid pJS25, to identify candidate proteins with a typical helix-turn-helix domain, or the ability to bind DNA. The candidate proteins were then compared by the BLASTp algorithm with the previously identified anti-CRISPR genes acting on systems type IE and IF (Bondy-Denomy et al., 2013; Pawluk et al., 2015) (Additional file 25), however no similarities were found. Determining the activity of these putative anti-CRISPR genes requires further experiments, which are out of the scope of this study.

#### Restriction-modification systems

To gain some insights into the possible restriction-modification (RM) systems present in the 20 strains studied here, we first analyzed the genome sequences for the presence of genes that could be identified as components of RM systems. This was accomplished using SEQWARE and the REBASE database as outlined previously [43]. In this way, 216 different RM system genes could be identified associated with 127 different systems. For many of them, putative recognition sequences could be assigned based on similarity to well-characterized RM systems in other organisms. Next, we took advantage of the fact that PacBio sequencing can detect the methylated bases, m6A and m4C, and the motifs in which they occur can be assigned [44]. Most strains contained more than one motif, although one strain, JS4, was devoid of apparent methylase activity and one strain, JS10, had an unusual motif characteristic of a Type I RM system, but with only one of the two sub-motifs methylated. The reason for this, as well as its significance, are unknown. Among the remaining eighteen strains, forty-nine motifs were found.

To assign the methylase genes responsible for each of the motifs we used a combination of direct assignment when a gene had very high similarity, usually greater than 90%, to a known gene or by noting when there was only candidate for a gene of a given Type of RM system. For instance, Type I RM systems have recognition sequences which are split into two sub-motifs containing 2–5 specific bases separated by a spacer of four to nine non-specific bases. Finally, once the easily identified motifs were matched to the genes encoding the methylases responsible a process of elimination was used to assign a few of the remaining matches. In this way, all but four of the motifs could be matched unambiguously to the genes encoding the responsible methylases (Table 5, Additional file 26). Among the strains, JS2 and JS7 had three Type I systems, while six strains had two such systems and nine strains had a single system. In all of these strains, except JS10, the R gene responsible for restriction was intact and the level of methylation was close to complete. This suggests that the systems were active as RM systems. It should be noted that many of the specificities were unique or newly found in this genus.

**Table 5:**
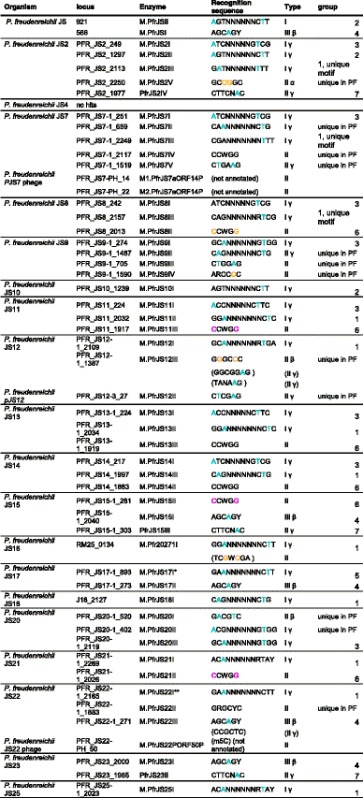
Methylation motifs and the responsible methylases identified in *P. freudenreichii*

Recognition sequences in parentheses indicates the methylase responsible cannot be assigned unambiguously. Modified bases are marked in colour; “not annotated” indicates there is no locus tag as the gene is not annotated in the GenBank file. Color coding for the methylation types Blue: 6 mA; Orange: 4mC; Purple: 5mC

*It is possible that it is really M.PfrJS17ORF2252P that is the active methylase in this organism. M.PfrJS17ORF2252P shares 99% aa sequence identity with M.PfrJS22I, but its corresponding S protein is truncated

**It is possible that it is really M.PfrJS22ORF850P that is the active methylase in this organism. M.PfrJS22ORF850P is 100% identical to M.PfrJS17I, as are their corresponding S proteins while the R proteins differ by one amino acid (475 Ala:Gly)

BLASTp analysis of the RM genes revealed that one variant of the Type I (group 1 in Table 5) system is widespread among the *P. freudenreichii* strains tested, located in 18 of the sequenced strains, with the exception of the strains JS9 and JS20. However, in six strains: JS (M.PfrJSII), JS4 (PFR_JS4_139), JS10 (M.PfrJS10ORF2151AP), JS15 (M.PfrJS15ORF135P), JS17 (M.PfrJS17ORF2252P) and JS23 (M.PfrJS23ORF2177P) the systems were inactive, most likely due to transposon-mediated inactivation of methylases (JS and JS10) or genes coding for specificity proteins (JS15, JS17 and JS23) or due to other, undetermined reasons (JS4 and JS23). Interestingly, even though the methylases from this group of RM systems were 97–100% identical on the amino acid level, the differences in specificity proteins resulted in differing recognition sequences. Strikingly, the amino acid sequences of the specificity proteins PFR_JS18_2128 and PFR_JS18_2129 aligned 100% to regions of the specificity protein PFR_JS8_2158. DNA sequence alignment of the coding regions showed that an insertion of cytosine at position 2,404,971 in JS18 DNA caused a frameshift, splitting the specificity gene into two otherwise 100% identical genes. It is worth noting that the recognition sequences associated with the corresponding RM systems in strains JS18 and JS8 are CAGNNNNNNCTG and CAGNNNNNNRTCG, respectively. The other variants of Type I systems (group 2, 3 and “unique in PF” in Table 5) differed from each other on the sequence level, but were highly conserved within the groups, with identical recognition sequences within groups.

In addition to the Type I systems, there were also examples of Type II methylases including examples of the same specificity in several strains, for instance seven strains contained a methylase recognizing CCWGG, identical in 6 of the strains (group 6 Table 5) and distinct in strain JS7 (M.PfrJS7IV), which is commonly found in many different genera. The RM system located on the putative plasmid JS12 (PfrJS12II) was active and unique among the *P. freudenreichii* strains included in this study. However, a BLASTn search revealed that the contig NZ_CDAG01000006.1 from the draft genome of the strain CIRM-BIA 456 showed 99% identity over 21% of the JS12 putative plasmid sequence (13756–18,773), spanning 48% of the contig sequence (150–5167) and encompassing the restriction-modification system PfrJS12II.

While some of the methylase motifs identified here were identical with previously known ones, the Type IIG enzymes were all new and unique. One Type III RM system, with the unique recognition sequence AGCAGY, was found in five of the strains.

### Vitamin B12 biosynthetic pathway

The vitamin B12 biosynthetic pathway in *P. freudenreichii* has been resolved previously [21, 45–47] and the organisation of the genes has been described earlier [21]. All the strains included in this study demonstrated an ability to produce active vitamin B12 [48] and we confirmed that all the strains possess the previously identified genes, in similar organisation, and highly conserved (protein alignments can be found in Additional file 27). Strain JS4 is an exception since *hemL* and *cbiD* genes are shorter, and the gene *cbiX* appeared to be missing. However, it was determined that in this strain a one-nucleotide-shorter spacer region resulted in a frameshift, which in turn led to the formation of a fusion gene of *cbiX* with the preceding gene *cbiH*. This result was confirmed by visual assessment of ten consensus sequence reads, arising from separate sequenced molecules from the PacBio assembly, that were aligned to the region. Nine out of these ten reads supported the observed deletion of a guanine base in the region, which causes the frameshift. In addition, *cbiX* and *cbiH* have 18 and 15 nucleotides variation in predicted sizes between strains, respectively, pointing to the variable character of the spacer region.

The B12 biosynthetic pathway is known to be regulated at the translational level by the cobalamin riboswitches [49]. In *P. freudenreichii* three of those riboswitches have been found upstream of genes *cbiL*, *cbiB* and *mutA* [50]. The B12-riboswitches in *P. freudenreichii* are not well characterized and the actual span of the element is not known but all the elements are expected to possess the conserved B12 binding region termed the B12-box, which is characterized by a consensus sequence rAGYCMGgAgaCCkGCcd [50]. Based on the previous reports [49, 50], we retrieved the predicted sequences for the three putative riboswitches and compared them among strains. All the *P. freudenreichii* strains possess the expected three riboswitches, which are highly conserved between the strains, with the B12-box consensus for the species: SAGYCMSAMRMBCYGCCD (Additional file 28). The actual effect of the riboswitches on the expression of the downstream genes is yet to be addressed. What may be of interest is that the riboswitches of the genes *cbiL* and *mutA* are located very close to each other, in opposite orientations, and can therefore interact.

### Pili and mucus binding

**Fig. 9:**
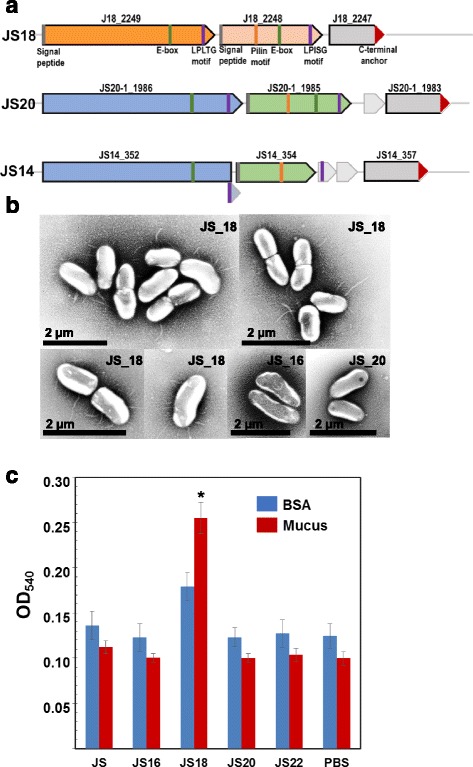
Pilus and mucus binding of *P. freudenreichii*. **a** Pilus operons predicted by LOCP. **b** Transmission electron microscopy (TEM) images of the strains with intact operons (JS18 and JS20) and control (JS16). **c** Adhesion assay of specific binding to porcine mucus and non-specific binding to BSA with cell-free PBS as control. The difference in specific binding of strain JS18 to mucus compared to non-specific binding to BSA and background PBS was statistically significant (*p* < 0.05) and is marked with asterisk

The search for pilus gene clusters using the LOCP tool [51] identified putative pilus operons in the genomes of JS18, JS20 and JS14 consisting of three, four and five ORFs, respectively (Fig. 9a). The first genes of each operon (PFR_J18_2249, PFR_JS20–1_1986, PFR_JS14_352) are predicted to encode surface-anchored fimbrial subunits, whereas class C sortases are the predicted function of the last genes (PFR_J18_2247, PFR_JS20–1_1983, PFR_JS14_357) in these clusters. PFR_J18_2248, PFR_JS20–1_1985 and PFR_JS14_354 located in the middle of the operons are predicted to encode type-2 fimbrial major subunits, in JS18, JS20 and JS14, respectively. The putative pilus operons in JS14 and JS20 are similarly organized and the predicted pilus proteins share 99–94% amino acid identity. ORF prediction and location of functional domains in predicted proteins suggest that in the JS14 genome the genes coding for the putative surface-anchored fimbrial subunit and the Type-2 fimbrial major subunit have been split, possibly due to frameshifts causing mutations. A BLAST search revealed the presence of highly conserved gene clusters in the pilus operons of JS14 and JS20, with similar structural organisation, in all the *P. freudenreichii* genomes studied here. The exception is strain JS9, where only partial genes coding for the surface-anchored fimbrial subunit (PFR_JS9–1_404) and the sortase (PFR_JS9–1_414) are found with a genomic island (Unique gene cluster 1) inserted between them (see Additional file 7). In contrast, homology searches revealed that the PFR_J18_2249-PFR_J18_2248-PFR_J18_2247 operon located in a genomic island that is unique to the JS18 strain, since no counterpart of the intact operon was identified in the other genomes. Based on BLAST searches, the genome of JS9 carries genes encoding the putative surface-anchored fimbrial subunit (PFR_JS9–1_546) and the Type-2 fimbrial major subunit (PFR_JS9–1_547) with 100% identity to the gene products of JS18 genome, but the third gene encoding the putative sortase is not present. The predicted pilus proteins encoded by JS18 operon share 32–54% identity with their counterparts encoded by the JS14/JS20 operon and BLASTp search against the NCBI non-redundant protein database revealed the highest amino acid identity (39–55%) with proteins of *Haematomicrobium sanguinis*.

Since in silico searches suggested that the genomes of JS18 and JS20 carry intact pilus operons, these strains were chosen for electron microscopic (EM) analyses together with the *P. freudenreichii* type strain JS16. Transmission electron microscopic images of negatively stained cells showed that the surfaces of JS18 cells contain pilus-like appendages, which were not observed in JS20 and JS16 cells (Fig. 9b).

Since EM revealed pili-like structures on the surface of the JS18 cells and pili contributes to probiotic properties by mucin binding in other bacteria [52], we next tested the adherence of the JS18 strain to mucus and to bovine serum albumin. For comparison also strains JS, used previously in mucus adhesion assays [19, 20], JS16 (type strain), JS20 with intact pilus operon and JS22 showing variable adherence in biofilm assay were included in the experiment. The results revealed adherence ability in strain JS18, which unlike other *P. freudenreichii* strains shows more efficient binding to mucus than to bovine serum albumin (Fig. 9c).

## Discussion

In this study we determined whole genome sequences of 17 new *P. freudenreichii* strains and re-sequenced whole genome of the strain DSM 4902. Using a comparative genomics approach, we identified several thus far unknown features of this species.

The comparative analyses revealed that despite genome-wide collinearity, large regions of inversions and other types of re-organisations are present even among the most closely related *P. freudenreichii* strains. This finding is of interest, as it was recently reported that in the closely related *Cutibacterium acnes* (previously *Propionibacterium acnes*) the gene synteny is highly conserved between strains [53]. These rearrangements could serve as an explanation for the observed adaptability and hardiness of *P. freudenreichii*, as the resulting genome instability was suggested as a driving force of adaptation and evolution in bacteria [54]. These genome reorganisations in *P. freudenreichii* appear to be transposon-mediated as transposase genes are found on the edges of many of the locally collinear blocks. The fact that in eight of the strains population diversity due to the translocation of transposase genes was observed suggests that transposons play an important role in genome plasticity in *P. freudenreichii* and explain this organism’s ability to adapt to various environments.

The genomes of three active temperate bacteriophages were discovered as circular molecules from the *P. freudenreichii* strains studied here. Relatively little is known about phages infecting Propionibacteria in general and *P. freudenreichii* in particular. At the time of writing of this study there are ten complete bacteriophage genome sequences available: nine tailed phages belonging to the order Siphoviridae and one Inoviridae (filamentous) phage. The filamentous phage B5 [55, 56] and the tailed phages: B3, B22, E1, E6 and G4 [57, 58], isolated in France from Swiss-type cheeses as well as previously uncharacterised Doucette and Anatole were sequenced in 2015 at the UCLA Genotyping and Sequencing Core Facility [59, 60]. The tailed PFR1 and PFR2 were induced directly from a Christian Hansen strain FD DVS PS-1 and a Swiss-type cheese purchased in Australia, respectively [61]. The phages identified in this study, namely PJS7, PJS22 and PJS23 are each the most similar to the previously reported PFR1, B22 and Doucette, respectively, but it is the first study reporting the presence of *P. freudenreichii* bacteriophage sequence present both as a prophage and also in free, circular form. In addition, an apparently complete prophage was identified from the strain JS17, but its circular form was not observed. In the previous study, the bacteriophage PFR1 was shown to infect *C. acnes* strain as well, while the PFR2 differing from PFR1 only by the presence of a transposable element could not [61]. The transposable element found in the PFR2 genome shares 100% DNA sequence identity to the PFR_JS12–1_615, PFR_JS15–1_2045 and PFR_JS17–1_657 found in strains JS12, JS15 and JS17, respectively and also to the prophage-encoded PFR_JS17–1_2067 of the strain JS17. This suggests that the insertion of the transposable elements intrinsic to the strain into the prophage-coding region could serve as a strategy for better control of the prophages.

At present, there are only a few published reports available concerning plasmids of *P. freudenreichii* [62–67]. Currently, four *P. freudenreichii* plasmid sequences are accessible at NCBI: p545 (AF291751.1), pRGO1 (NC_002611.1), pLME106 (NC_005705.1) and pLME108 (NC_010065.1). Here, we report sequences of two additional plasmid-like elements PFRJS12–3 and PFRJS25–1. It is noteworthy that the circular element PFRJS25–1 appears to be widespread among *P. freudenreichii* strains. We compared the recently published draft genomes [21, 23, 24] and detected sequences with 99% identity over 90% of the sequence of the putative plasmid PFRJS25–1 to the contigs in *P. freudenreichii* strains ITG P20 (CIRM-BIA 129) (NZ_HG975461 and CCBE010000014), ITG P1 (CCYV01000031), CIRM-BIA 135 (CCYZ01000006) and CIRM-BIA 1025 (NZ_CCYV01000031). Because of the limited size of the contigs, it is impossible to determine whether in these strains the elements were circular or existed as a part of the chromosome. We explored the possibility that the circular elements are a type of Integrative and Conjugative Elements (ICEs), which are widely distributed mobile genetic elements existing normally within the host’s chromosome. Under certain conditions those elements can become activated, excise from the chromosome and transfer to a new recipient [68]. Although no such elements have been described in Propionibacteria so far, they are widespread among other Actinobacteria in two types [69]. The FtsK/SpoIIIE-type relies on a single FtsK/SpoIIIE-like protein for DNA translocation, while the T4SS-type requires an assembly of a complex type IV translocation system for mobility [69]. The previously mentioned Unique gene clusters 4,9,11,14 and 16 (see Fig. 4), found in strains JS12, JS18, JS20, JS7 and JS9, respectively, share structural similarities indicative of ICEs type T4SS that are also present in PFRJS12–3 and PFRJS25–1. Thus, it is possible that the extrachromosomal circular DNA elements in JS12 and JS25 represent mobilised ICEs instead of plasmids. Nevertheless, further studies are necessary to determine the true nature of these new, *P. freudenreichii*-specific elements.

Complete CRISPR-Cas systems were identified in 15 out of 20 sequenced strains and for the first time they were classified for *P. freudenreichii* as type IE, found in three strains and a novel type IU, found in 13 strains. The activity of CRISPR-Cas systems in *P. freudenreichii* should be addressed in further studies.

Very little is known about restriction-modification (RM) systems in *P. freudenreichii*. It has been shown that such systems are present based on the observed interference with transformation efficiencies [66] and the host range dependent on the compatibility of the RM systems between the sources and intended hosts of the plasmids [65]. In this study, the most striking feature of the RM systems distributed among the *P. freudenreichii* strains was the variability of the systems and the genomic locations found from one strain to another. This contrasts with the more usual situation where, at the very least, there are one or more common methylases found in all strains of a particular species (see REBASE [70]).

Pili or fimbriae are surface adhesive components and well-documented virulence factors for many harmful opportunistic pathogens [71]. On the other hand, the role of these non-flagellar proteinaceous hair-like fiber appendages in probiotics and/or commensal bacteria and as niche-adaptation factors has only recently been recognized. Among Actinobacteria, pili have been reported to be crucial for establishing both host-microbe and microbe-microbe interactions in probiotic Bifidobacteria [72, 73], while in *Propionibacterium* pili have not been described. Here, we identified a unique pilus operon from a genomic island in a *P. freudenreichii* strain and show that cells of this strain are decorated by pili-resembling structures. Furthermore, the strain with pili-like appendages was capable of specific mucus binding, while *P. freudenreichii* strains generally bind similarly to mucus and bovine serum albumin through non-specific interactions [19, 20].

## Conclusions

The whole genome alignments showed that, despite genome-wide collinearity, large regions of transposon-mediated inversions and other types of re-organisation are present in *P. freudenreichii* genomes. The fact that in eight of the strains we observed population diversity due to the translocation of transposase genes suggests that transposable elements play an important role in genome plasticity in *P. freudenreichii* and explain this organism’s ability to adapt to various environments, while the additional role of the transposons in control of the prophages and CRISPR-Cas systems needs to be explored further.

The utilization of long read technology enabled us to correctly assemble the genomic elements, such as phages, that were found both within and outside the genomes. These separate sub-populations could have been missed with short read data, even when using mate-pair or other long fragment-based methods. The long reads additionally enabled detailed analysis of the CRISPR arrays and allowed characterisation and classification of the CRISPR-Cas systems. The use of the PacBio sequencing platform, which detects methylation patterns, also permitted the detection of potentially active restriction-modification systems through matching of the recognition motifs with the methylases responsible. Many of the recognition sequences of these RM systems identified in this study are being reported for the first time. Finally, we report the first evidence of a *P. freudenreichii* strain being decorated by pili appendages and showing specific mucus binding. Taken together, the whole genome sequencing from long reads proved to be a useful method for improving the characterisation of *P. freudenreichii* by allowing the discovery of previously uncharted territories for the species. The amassed data provides a firm foundation for further, more in-depth studies of the species.

## Methods

### Bacterial growth and extraction of DNA

The strains were grown in propionic medium (PPA) [19] or the whey-based liquid medium (WBM) [48]. The PPA composition was: 5.0 g. tryptone (Sigma-Aldrich), 10.0 g. yeast extract (Becton, Dickinson), 14.0 ml 60% *w*/w DL-sodium lactate (Sigma-Aldrich) per liter, with pH adjusted to 6.7 prior to autoclaving. The industrial-type medium, WBM, was composed of 60.0 g of filtered whey powder (Valio Ltd., Finland), 10.0 g of yeast extract (MERCK, KGaA), 0.1 g Tween 80 (MERCK, KGaA), 0.2 g magnesium sulphate (MERCK, KGaA), 0.05 g manganese (II) sulphate (MERCK, KGaA), 100 mM potassium phosphate buffer (MERCK, KGaA) and was prepared as previously described [48].

For the phenotypic tests the strains were grown in YEL [74] medium composed of 10 g of tryptone (Sigma-Aldrich), 10 g yeast extract (Becton Dickinson), 16.7 g of 60% w/w DL-sodium lactate (Sigma-Aldrich), 2.5 g K2HPO4, 0.005 g MnSO4.

The cultures were prepared from 15% glycerol stocks stored at −80 °C by streaking on a PPA agar plate and incubation at 30 °C in anaerobic jars (Anaerocult, Merck, Germany) for 4 days, unless stated otherwise. For the preparation of liquid cultures, colonies from the plate were picked and transferred to 15 mL Falcon tubes containing 10 mL of the liquid medium.

For the DNA extraction, the cells were harvested from liquid cultures incubated for 72 h by centrifugation for 5 min at 21000 g at 4 °C and washed with 0.1 M TRIS pH 8.0. The DNA extraction was performed with ILLUSTRA™ bacteria genomicPrep Mini Spin Kit (GE Healthcare) with 10 mg of lysozyme and the incubation time 30 min.

### Genome sequencing and assembly

Nineteen *Propionibacterium freudenreichii* samples were sequenced with the Pacific Biosciences RS II Instrument using either P4/C2 or P5/C3 chemistries (listed in Table 2). Two SMRT cells were used for each sample. Movie times varied from 120 to 240 min. The total number of obtained bases and subreads and their mean and N50 lengths are listed in Table 2. The Hierarchical Genome Assembly Process (HGAP) V3 implemented in the SMRT Analysis package (v.2.3.0) was used to generate de novo genome assemblies with default parameters, excluding the genome size estimate parameter which was set to 3,000,000 bp. Obtained circular sequences were polished using SMRT Analysis RS Resequencing protocol and the Quiver consensus algorithm. Chromosomal genome sequences were set to begin from the chromosomal replication initiator protein (dnaA). The sequences were then annotated with Prodigal v. 2.6.2. All sequences were deposited in the European Nucleotide Archive (ENA). Complete genome, phage and plasmid sequence sizes, sequencing coverages, GC percentages, number of predicted genes and ENA accession numbers are listed in Table 1. Base modifications and motifs were detected using RS Modification and Motif analysis protocol (SMRT Analysis package v.2.3.0).

### Bioinformatics analyses

Average nucleotide identities (ANI) were calculated using JSpecies V1.2.1 [75]. Genome organizations were visualized with the Mauve alignment tool using the Progressive Mauve algorithm [76] with GenBank input files generated by conversion of the EMBL files obtained after the submission of genomes to the European Nucleotide Archive (ENA) with the use of Seqret, a part of the EMBOSS package. Like many other packages used in this study EMBOSS was a part of the BioLinux 8 worksation [77].

The core and pan-genome was estimated with Roary [32]) at standard settings with GFF3 annotation files generated by PROKKA [78] used as input files. The genomic islands were detected with the help of IslandViewer 3 software [35]. For the prediction of prophages, Prophinder [79] and Phaster [80] online software were used. Predicted bacteriophages were then reviewed visually for structural completeness.

Prediction of CRISPR loci was aided by the CRISPRFinder [81]. The obtained results were then reviewed manually for co-localisation with Cas genes. The immunity to known bacteriophages was tested by searching the spacer sequences against NCBI tailed bacteriophages and also whole nucleotide collection excluding Propionibacteria (txid1743) with the aid of the BLASTn suite. Restriction-Modification systems were identified using SEQWARE [43] and the REBASE database [70] followed by manual matching of methylase genes with predicted recognition sequences against the methylation profiles generated by PacBio sequencing. In some cases, matches could be unambiguously inferred when only a single methylase gene and a single motif were present or left unmatched. Automatic pilus cluster search was performed using LOCP v. 1.0.0 [51]. The results were visualised with iTOL [82], Phandango 0.8.5 [83], EasyFig [84] and PigeonCad [85].

### PCR reactions

All of the PCR reactions were performed with the Phusion (ThermoFisher Scientific) mastermix with 0.3% DMSO and the primers prepared by Oligomer Oy (Helsinki, Finland). The results were visualised by 0.8% agarose (BioRad) gel electrophoresis with ethidium bromide (0.5 μg/ml) (Sigma-Aldrich) staining.

#### Phage integration analysis

The phage detected in the strain JS7 was found both in a free, circular form as well as integrated into the chromosome as was the case for strains JS22 and JS23. However, in strain JS7 a phage-free bacterial genome was detected as well, which allowed us to study the dynamics of phage integration and release from the chromosome. For that purpose, PCRs were designed to amplify the regions of both the region of phage integration in the bacterial chromosome as well as the attachment sites on the bacteriophage genome. The primers used were: PB5 CGCATACGCAGATATTAAG complementary to the 5′ end of the gene coding for the KmtR transcriptional regulator, PB6 GAGGTGCTGGCGGATAC complementary to the 3′ end of the transcriptional regulator located just downstream of the CmtR regulator- coding gene in the phage-free chromosome, PB7 CTTCCCGCAGTGTCTTG and PB8 GAAGCAGGGCGTTTATG both complementary to the phage-encoded Integrase. The reaction mix PB5 and PB6 would therefore detect a phage-free bacterial chromosome with the 815 nt long product; PB6 and PB8 would detect a chromosome with the phage integrated with the 691 nt product; PB7 and PB8 would detect a circular phage with the 850 nt product. The reactions were performed on bacterial cells picked from separate colonies grown on PPA agar for 4 days in three separate experiments. For one set of colonies PCRs were repeated on after 7 days. The same colonies were then picked and propagated every 7 days for 10 generations. The PCR was repeated after 5 and after 10 generations.

#### Duplication in the strain JS17

In order to eliminate the possibility that the duplication observed in the strain JS17 is a result of a sequencing error we analysed the edges of the region by PCR. The primers used were: PD6 fwd CTGGTTGCGTCATCTCTAAGCCT, PD7 rev CGCTCTTTTAGGGAATCGCTCAT and PD8 fwd TCTTCTTCTGTACGCGTGGACAT. The PCRs were performed with PD6 and PD7, PD8 and PD6 and also single PD6 and PD8 as negative controls. Because of the high melting temperatures of the primers, the PCRs were performed with a 2-step protocol (annealing and extension combined at 72 °C for 1:30 min). The reactions were deemed positive when the products of sizes 1888 nt for the PD6-PD7 and 1738 nt for PD7-PD8 were seen on the 0.8% agarose gel.

#### Electron microscopy for detection of pili

The strains were grown on YEL agar at 30 °C for 7 days under near-anaerobic atmosphere (Anaerocult, Merck) Single colonies were picked and suspended in 0.1 M PIPES buffer, pH 6.8. An aliquot of 3 μl was added to Pioloform-coated 200-mesh copper grids previously glow discharged (Emitech K100X, Emitech Ltd., UK) to ensure even adhesion of the bacterial cells to the grids. After 1 min incubation, the excess suspension fluid was removed by soaking with a filter paper. The grids were negatively stained with 1% neutral uranyl acetate for 15 s and air-dried. Images were acquired at 120 kV with a Jeol JEM-1400 microscope (Jeol Ltd., Tokyo, Japan) using an Orius SC 1000B CCD-camera (Gatan Inc., USA).

#### Mucus adhesion assay

Adhesion of the *P. freudenreichii* strains (JS, JS16, JS18, JS20 and JS22) on immobilized porcine mucin (Sigma-Aldrich) in 96-well Polysorp microplates (Nunc Immuno plates, Nunc, Denmark) was conducted according to Lecesse Terraf [86] with the following modifications. Shortly, plates were covered with 300 μl of 0.2 mg ml^−1^ mucin in phosphate buffered saline (PBS, pH 7.5) (Thermo Fischer Scientific) and incubated for 30 min at 37 °C (250 rpm, PST-60HL Thermo-Shaker (Biosan) and then overnight at 4 °C. Wells were washed twice with PBS and blocked with 1% bovine serum albumin (BSA) in PBS, at room temperature. Replica microplates treated with PBS without mucin and then 1% BSA as above were also prepared to exclude BSA-specific binding. After 2 h incubation, mucin- and BSA-coated wells were washed twice with PBS and allowed to dry. For adhesion, 200 μl of cells suspended in PBS (OD_600_ = 2.0) from each strain was added into the mucus- and BSA-coated wells, and plates were incubated at 37 °C for 2 h (250 rpm). PBS alone was included in all experiments to subtract mucin binding by PBS. Non-adherent cells were removed and wells were washed with PBS. Then, adherent cells were stained with 200 μL of the crystal violet solution (0.1%, *w*/*v*) (Sigma–Aldrich, Munich, Germany) for 30 min at room temperature. Excess stain was washed off with deionized H_2_O and the stained cells were suspended in 30% acetic acid by shaking (400 rpm) at room temperature, and recorded at 540 nm using an ELISA reader (Labsystems Multiskan EX). Up to four independent experiments were performed, each with at least sixteen technical replicates.

Significant differences between sample means of independent experiments and the mean of PBS as test value were determined by one-sample t-test. *P* values <0.05 were considered statistically significant. The calculations were performed with statistical package program (IBM SPSS Statistics v24 for Windows, IBM, USA).

## Additional files

Additional file 1: Gene-presence-absence table generated by ROARY analysis. (XLSX 893 kb)

Additional file 2: Annotation file of the strain JS with the cumulative results of bioinformatics analyses. (XLSX 331 kb)

Additional file 3: Annotation file of the strain JS2 with the cumulative results of bioinformatics analyses. (XLSX 346 kb)

Additional file 4: Annotation file of the strain JS4 with the cumulative results of bioinformatics analyses. (XLSX 401 kb)

Additional file 5: Annotation file of the strain JS7 (LT618776) and of the phage PJS7 (LT618778) with the cumulative results of bioinformatics analyses and the results of the phage integration study. (XLSX 360 kb)

Additional file 6: Annotation file of the strain JS8 with the cumulative results of bioinformatics analyses. (XLSX 329 kb)

Additional file 7: Annotation file of the strain JS9 with the cumulative results of bioinformatics analyses. (XLSX 406 kb)

Additional file 8: Annotation file of the strain JS10 with the cumulative results of bioinformatics analyses. (XLSX 315 kb)

Additional file 9: Annotation file of the strain JS11 with the cumulative results of bioinformatics analyses. (XLSX 298 kb)

Additional file 10: Annotation file of the strain JS12 (LT604998) and of the putative conjugative plasmid PFRJS12–3 (LT604882) with the cumulative results of bioinformatics analyses. (XLSX 386 kb)

Additional file 11: Annotation file of the strain JS13 with the cumulative results of bioinformatics analyses. (XLSX 307 kb)

Additional file 12: Annotation file of the strain JS14 with the cumulative results of bioinformatics analyses. (XLSX 304 kb)

Additional file 13: Annotation file of the strain JS15 (DSM 4049) with the cumulative results of bioinformatics analyses. (XLSX 323 kb)

Additional file 14: Annotation file of the strain JS16 (DSM 20271) with the cumulative results of bioinformatics analyses. (XLSX 357 kb)

Additional file 15: Annotation file of the strain JS17 with the cumulative results of bioinformatics analyses. (XLSX 391 kb)

Additional file 16: Annotation file of the strain JS18 with the cumulative results of bioinformatics analyses. (XLSX 379 kb)

Additional file 17: Annotation file of the strain JS20 with the cumulative results of bioinformatics analyses. (XLSX 413 kb)

Additional file 18: Annotation file of the strain JS21 with the cumulative results of bioinformatics analyses. (XLSX 335 kb)

Additional file 19: Annotation file of the strain JS22 (LT599498) and of the phage PJS22 (LT615138) with the cumulative results of bioinformatics analyses. (XLSX 347 kb)

Additional file 20: Annotation file of the strain JS23 (LT618794) and of the phage PJS23 (LT618793) with the cumulative results of bioinformatics analyses. (XLSX 327 kb)

Additional file 21: Annotation file of the strain JS25 (LT618783) and of the putative conjugative plasmid PFR_JS25–1 (LT618784) with the cumulative results of bioinformatics analyses. (XLSX 336 kb)

Additional file 22: Phenotypic characterization. The characteristics of the studied strains were assessed and included: carbohydrate utilization patterns, nitroreductase activity as well as growth and biofilm formation in various growth conditions. (DOCX 612 kb)

Additional file 23: Traits important in food production. Genes coding for traits found as important in food production are explored. (PDF 8 kb)

Additional file 24: The CRISPR spacers within CRISPR arrays identified in *P. freudenreichii.* The collection of all CRISPR spacers predicted in the *P. freudenreichii* strains together with their predicted immunity. (PDF 75 kb)

Additional file 25: Self-immunity: the table of spacers conferring self-immunity; Candidate proteins: the summary of the candidate proteins for Anti-CRISPR elements on the invading DNA elements. (XLSX 13 kb)

Additional file 26: Summary schematics of the RM systems in *P. freudenreichii* strains. (PDF 1724 kb)

Additional file 27: Protein sequence alignments of the proteins involved in biosynthesis of vitamin B12. (XLSX 131 kb)

Additional file 28: The alignment of the three predicted B12 riboswitches with the conserved B12-box highlighted. (PDF 28 kb)

## Abbreviations

ANI: Average nucleotide identity
BSA: Bovine serum albumin
CAS: CRISPR associated proteins
CDD: Conserved Domains Database
CRISPR: Clustered regularly interspaced short palindromic repeats
ENA: European Nucleotide Archive
GRAS: Generally Recognized as Safe
HGAP3: Hierarchical Genome Assembly Process
ICEs: Integrative and Conjugative Elements
OD_600_: Optical density measured at 600 nm
PBS: Phosphate buffered saline
PPA: Propionic medium
RM: Restriction-modification
SCFAs: Short-chain fatty acids
