# Supplementary material for: De novo assembly of genomes from long sequence reads reveals uncharted territories of Propionibacterium freudenreichii
Source: BMC Genomics. 2017 Oct 16;18:790. doi: 10.1186/s12864-017-4165-9 (PMC5644110; doi:10.1186/s12864-017-4165-9)
Supplement: Supplementary file 22 — Phenotypic characterization. The characteristics of the studied strains were assessed and included: carbohydrate utilization patterns, nitroreductase activity as well as growth and biofilm formation in various growth conditions. (DOCX 612 kb) [file 12864_2017_4165_MOESM22_ESM.docx]

#### Phenotypic characterisation

*P. freudenreichii* is traditionally used in the production of Swiss-type cheeses and therefore the phenotypic characterisation of the strains usually involves assessment of traits important in cheesemaking, including the ability to grow at low pH, at high salt concentrations and at low temperatures [1]. Here, we tested the ability of the strains to grow at pH 4.0, 4.5, 5.0 and 9.0, salt (NaCl) concentrations 0.3 M, 0.5 M and 1.1 M (corresponding to 1.5, 3 and 6.5%, respectively) and at temperatures of 12°C and 42°C expressed as values of optical density (OD) measured at 595 nm. In addition, we tested the ability to form biofilms at the same growth conditions using crystal violet staining of the biomass and the measurement of absorbance at 540 nm. To complete the phenotypic characterisation, we tested the historically important trait of nitroreductase activity in *P. freudenreichii* and also explored the carbohydrate utilisation patterns (Figure S1).

All the strains grew in the presence of 0.3 M and 0.5 M NaCl, while most (15/20) of the strains did not grow in media with 1.1 M NaCl, and only one strain (JS25) reached an OD_595_ value above 0.5 under this high osmolarity condition. All the strains could grow at pH 5.0 and pH 9.0 although to varying extents. Growth at pH 5.0 is consistent with a previous study reporting *P. freudenreichii* capable of initiating growth at the lowest pH of 5.0 [2]. Under the conditions used here, some of the strains were capable of initiating growth at pH 4.5, with the strains JS15 and JS22 reaching OD_595_ values slightly above 0.5 under this condition. None of the strains could grow at pH 4.0. Growth at 12°C could have important implications in cheesemaking, because similarly to high aspartase activity, it is associated with the split defect in Swiss-type cheeses [3]. Here, at 12°C no growth was observed for five strains (JS4, JS11, JS13, JS20 and JS21), while one strain (JS2) could grow to OD_595_> 0.5 at that temperature. At an incubation temperature of 42°C most of the strains failed to initiate growth while three strains (JS8, JS9 and JS14) showed weak growth (Figure S1).

To the best of our knowledge, biofilm formation has not been previously reported in *P. freudenreichii*. The closely related opportunistic pathogen *C. acnes* is a known biofilm former associated with development of acne vulgaris [4] and prosthetic joint infections [5] when growing in biofilm form. *C. acnes* biofilms were also recently implicated in strengthening biofilms of *Staphylococcus aureus* [6]. On the other hand, biofilm-forming probiotic strains of Lactobacilli effectively reduced the capability to form biofilms by food-borne pathogenic bacteria *Salmonella typhimurium* and *Listeria monocytogenes* [7]. In the current work, a microtiter plate-based model was used to test the biofilm formation capacity of the *P. freudenreichii* strains on an inert surface. Under the conditions used here, biofilm formation was observed for all the strains except for JS11 and JS16. Under control conditions (unmodified YEL [8], in anaerobic atmosphere at 30°C), a strong biofilm formation (OD_540_> 0.5) was observed only by three strains (JS, JS14 and JS15). On the other hand, eight strains (JS, JS4, JS9, JS14, JS17, JS20, JS22 and JS25) showed strong biofilm formation under high osmolarity conditions (1.1 M NaCl). While the strains JS9 and JS22 were unable to form a biofilm under control conditions, they formed biofilms under most of the other conditions tested (Figure S1). The implications of biofilm-forming capabilities of *P. freudenreichii* require further study.

All the strains could utilise glycerol, erythritol, glucose and inositol as carbon sources for growth. In addition, all the strains were able to utilise adonitol and potassium gluconate, although this ability was inconsistent in strains JS23 and JS9, respectively. Only strain JS21 was unable to use mannose, while in strain JS20 growth using mannose was observed only after 7 days’ incubation. Melibiose fermentation was observed only after 7 days by strains JS, JS7, JS8 and JS17. Xylitol was utilised by the strains JS2, JS7, JS8 and JS14 and this feature is possibly tied to the presence of genes coding for the type II PTS transport: PFR_JS2_252 (subunit IIB), PFR_JS2_253 (subunit IIC) and PFR_JS2_255 (subunit IIA), which were exclusively present in the four strains utilising xylitol. While the current prediction suggests function in galactitol transport for this system, none of the *P. freudenreichii* strains tested was able to utilise galactitol (dulcitol) indicating that other carbohydrates possibly including xylitol are substrates for this PTS system. Ability to utilise melibiose by the strains JS, JS7, JS8 and JS17 could be tied to the previously reported genes *rbsR*, *aga*, *msmE*, *amyD* and *amyC* [9] which in strain JS7 are encoded by PFR_JS7-1_172, PFR_JS7-1_171, PFR_JS7-1_170, PFR_JS7-1_169 and PFR_JS7-1_168, respectively. The strain JS10 also possesses the genes necessary for melibiose utilisation (PFR_JS10_179- PFR_JS10_175) which share 99-100% DNA sequence identity with the genes of the strain JS7, however the strain JS10 was not able to utilise melibiose under the conditions used here.


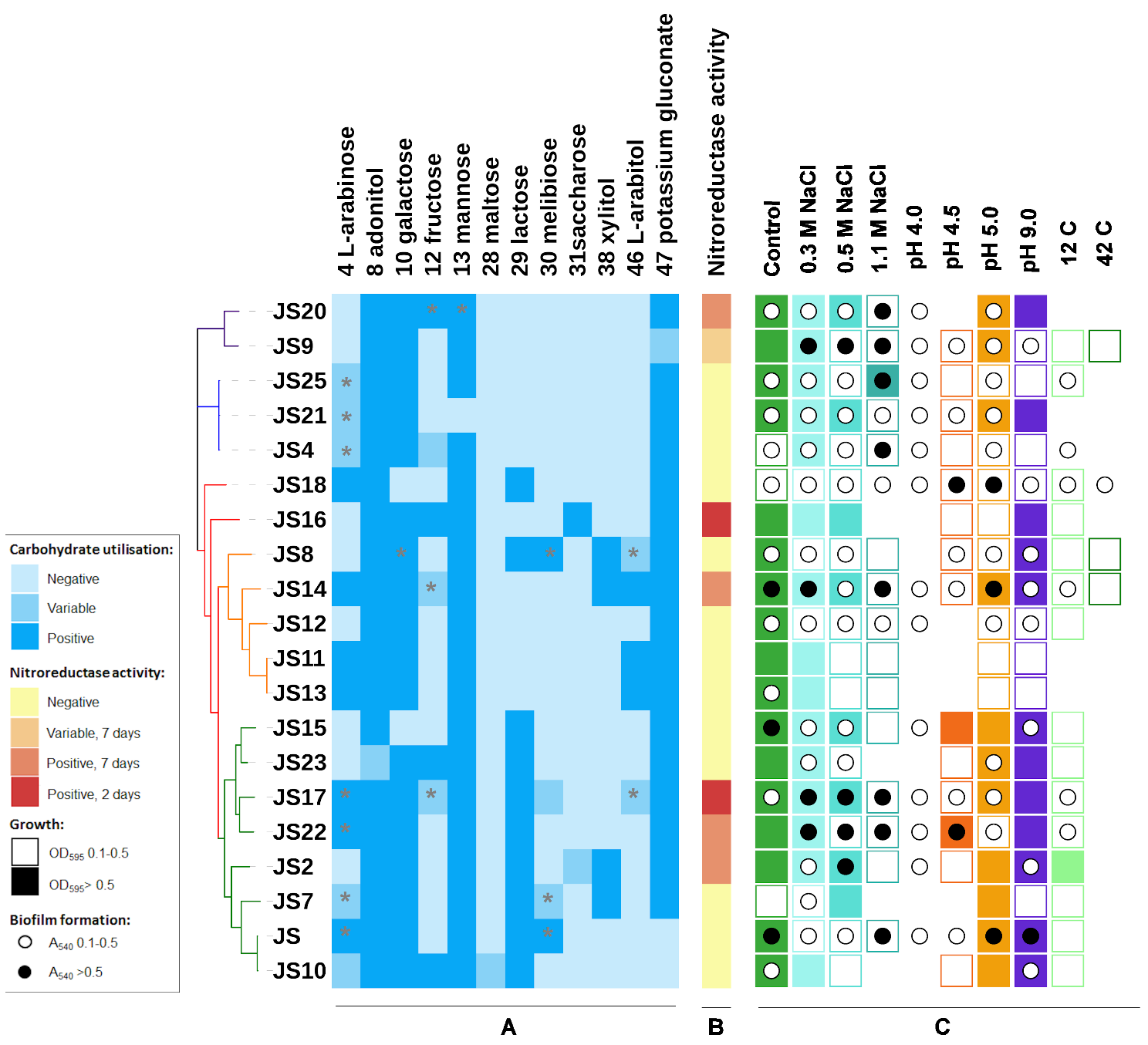


Figure S1 Phenotypic characterisation of the strains. Panel A) Carbohydrate fermentation profiles assessed with an API test. The results were marked positive if growth was observed each time the experiment was repeated (2-4 repetitions). The results were marked variable if growth was observed in at least half of the repeated experiments and otherwise negative. The asterisks denote growth observed only after 7 days incubation. Panel B) Nitroreductase activity measured from cultures grown for 2 and 7 days. Results expressed on four levels: positive after 2 days of growth, positive after 7 days of growth, variable when results were positive in half of the cultures after 7 days of growth, and no activity. Panel C). The ability to grow and to form a biofilm on microtiter plates in modified YEL medium under anaerobic atmosphere. Growth is expressed as OD_595_. Colored-in squares indicate OD_595_> 0.5 while empty squares correspond to OD_595_< 0.5. No growth was reported when OD_595_ was below 0.1 and no square was assigned. The ability to form a biofilm was measured by crystal violet staining and is expressed as absorbance at 540 nm (A_540_). Black circles indicate high level of biofilm formation (values of A_540_ >0.5). White circles correspond to low biofilm formation (A_540_ <0.5). No symbol indicates inability to form biofilm under tested condition (A_540_ <0.1). The figure was created with iTOL with the core genome alignment phylogenetic tree.

Lactose was used by ten of the strains, but none of them were of cereal origin. The ability was tied to the presence of genes PFR_JS15-1_238, PFR_JS15-1_239 and PFR_JS15-1_240 (in the type strain JS15) coding for UDP-glucose 4-epimerase (*galE1*), Sodium:galactoside symporter (*galP*) and Beta-galactosidase (*lacZ*) and located on a genomic island [10]. The unique feature of the cereal strains was the ability to ferment L-arabitol by the strains JS11, JS13 and JS14. The ability was also observed in strains JS8 and JS17, but in both cases only after 7 days of incubation, and in variable manner.

In previous reports, a frameshift in the gene *narG* was associated with the lack of nitroreductase activity, while the presence of the nar locus consisting of genes *modB, modA, mog, moaA, narK, narG narH,*

*narJ narI* and *moaE* was a good predictor of the nitroreductase activity [9]. In our study, 5/20 strains (JS, JS10, JS15, JS18 and JS23) appear to lack at least part of the locus, while the rest of the strains (15) carry the complete locus. Nitroreductase activity could be confirmed for seven strains only, suggesting strain variation in the expression of the nar locus under the conditions used. Following the standing criteria of classification into subspecies, namely the inability to utilise lactose and ability to reduce reductase for subspecies *freudenreichii* and vice versa for subspecies *shermanii*, it was impossible to classify half of the strains included in the study, because for strains JS2, JS17 and JS22 both decisive traits were positive, while for strains JS4, JS11, JS12, JS13, JS18, JS21 and JS25 both traits were negative. This supports the notion that the division of *P. freudenreichii* into subspecies is not warranted [11].

### **Materials and methods**

**Carbohydrate utilisation**

For the carbohydrate utilisation patterns API CHL50 tests (BioMerieux, France) were performed according to the manufacturer’s instructions, with some modifications. Shortly, the strains were grown on PPA or YEL plates for 4 days, then the cells were suspended in PBS and the optical density (OD) was measured at 600 nm. These suspensions were then used to inoculate the CHL50 media at levels corresponding to OD_600_ of 0.5. The tests were incubated for 7 days at 30°C and read at three and seven days.

**Nitroreductase activity**

For the nitroreductase activity test the strains grown in YEL medium for 3 days were inoculated at a level of 1% into two individual Eppendorf tubes containing 1.5 mL of nitrate medium [12] composed of 1.5 g potassium nitrate, 10.0 g tryptone (Sigma-Aldrich), 5.0 g yeast extract (Becton, Dickinson) and 1.0 g glucose (Sigma-Aldrich) per liter. The cultures were tested for nitroreductase activity after 2 and 7 days using Griess reagent (Biotium) according to the manufacturer’s instructions. Briefly, the cultures in Eppendorf tubes were centrifuged for 2 minutes at 4000 g at room temperature, then 150 µl of the supernatant was mixed with 130 µL of MilliQ water and 20 µL of Griess reagent in duplicate and incubated in the dark for 30 minutes on 96 wells polystyrene tissue-culture microtiter plates (353077, Falcon, Corning incorporated). After that time the plates were read with a Multiskan EX plate reader (Labsystems) and recorded.

**Growth and biofilm formation in various conditions**

For each strain, colonies from YEL plates were used to inoculate 10 ml of liquid YEL medium in three biological replicates followed by incubation under microaerobic atmosphere at 30°C for 3 days. The OD_600_ was measured for all of the cultures and used for inoculation of 190 µl of the test medium to an OD_600_ value of 0.05 in 96-wells polystyrene tissue-culture microtiter plates (353077, Falcon, Corning incorporated).

For the low/high pH challenge the YEL medium pH adjusted to 4.0, 4.5, 5.0 or 9.0 and filter-sterilized with 0.2 µm Nalgene Rapid-Flow filter units (Thermo Fisher Scientific). Effect of increased osmolarities to growth was tested in media containing 256.7 mM, 513.3 mM and 1.1 M NaCl. The cultures were incubated for 7 days at 30°C in an anaerobic atmosphere (Anaerokult, Merck). The ability to grow at 12°C and 42°C was tested in YEL using the inoculum standardized to OD_600_ of 0.1 and with additional four technical replicates for each culture. In all experiments, the growth was assessed by measurement of OD with a Multiskan EX plate reader (Labsystems) at 595 nm at 4 and 7 days. After 7 days, all of the cultures were checked for biofilm formation. Briefly: non-adherent cells were first removed from the plates by rinsing twice under cool tap water by immersing and shaking out. Subsequently, the wells were filled with 100 µL of 1% crystal violet, incubated for 10 minutes, and rinsed repeatedly by immersion in cool tap water until the water would remain clear. The plates were then left to dry at room temperature and the biofilms were dissolved in 200 µL of 30% acetic acid with shaking (300 rpm) for 30 minutes. The results were recorded with a Multiskan EX plate reader (Labsystems) at 540 nm.

### **References:**

1. Vorobjeva L. Biosynthetic processes and physiologically active compounds. In: Propionibacteria. Springer Netherlands; 1999. p. 149–51.
2. Jan G, Rouault A, Maubois J. Acid stress susceptibility and acid adaptation of *Propionibacterium freudenreichii* subsp. *shermanii*. Lait . 2000;80:325-36.
3. Daly DFM, McSweeney PLH, Sheehan JJ. Split defect and secondary fermentation in Swiss-type cheeses – A review. Dairy Sci. Technol. 2010;90:3–26.
4. Coenye T, Peeters E, Nelis HJ. Biofilm formation by *Propionibacterium acnes* is associated with increased resistance to antimicrobial agents and increased production of putative virulence factors. Res. Microbiol. 2007;158:386–92.
5. Achermann Y, Goldstein EJC, Coenye T, Shirtliffa ME. *Propionibacterium acnes*: From Commensal to opportunistic biofilm-associated implant pathogen. Clin. Microbiol. Rev. American Society for Microbiology; 2014;27:419–40.
6. Tyner H, Patel R. *Propionibacterium acnes* biofilm–A sanctuary for *Staphylococcus aureus*? Anaerobe. 2016;40:63-7.
7. Woo J, Ahn J. Probiotic-mediated competition, exclusion and displacement in biofilm formation by food-borne pathogens. Lett. Appl. Microbiol. 2013;56:307–13.
8. Malik AC, Reinbold GW, Vedamuthu ER. An evaluation of the taxonomy of Propionibacterium. Can. J. Microbiol. 1968;14:1185–91.
9. Loux V, Mariadassou M, Almeida S, Chiapello H, Hammani A, Buratti J, *et al.* Mutations and genomic islands can explain the strain dependency of sugar utilization in 21 strains of *Propionibacterium freudenreichii*. BMC Genomics . 2015;16:296.
10. Falentin H, Deutsch SM, Jan G, Loux V, Thierry A, Parayre S, et al. The complete genome of *Propionibacterium freudenreichii* CIRM-BIA1T, a hardy actinobacterium with food and probiotic applications. PLoS One. 2010;5.
11. Scholz CFP, Kilian M. The natural history of cutaneous propionibacteria, and reclassification of selected species within the genus *Propionibacterium* to the proposed novel genera *Acidipropionibacterium gen. nov.*, *Cutibacterium gen. nov*. and *Pseudopropionibacterium gen. nov*. Int. J. Syst. Evol. Microbiol. 2016;4422–32.
12. Thierry A, Deutsch S-M, Falentin H, Dalmasso M, Cousin FJ, Jan G. New insights into physiology and metabolism of *Propionibacterium freudenreichii*. Int. J. Food Microbiol. 2011;149:19–27.
