# Supplementary material for: De novo assembly of genomes from long sequence reads reveals uncharted territories of Propionibacterium freudenreichii
Source: BMC Genomics. 2017 Oct 16;18:790. doi: 10.1186/s12864-017-4165-9 (PMC5644110; doi:10.1186/s12864-017-4165-9)
Supplement: Supplementary file 23 — Traits important in food production. Genes coding for traits found as important in food production are explored. (PDF 8 kb) [file 12864_2017_4165_MOESM23_ESM.pdf]

Traits important in food production

P. freudenreichii plays an important role in cheesemaking, where it is used as a ripening culture. The primarily role of *P. freudenreichii* is to remove lactic acid produced by the starter strains. The metabolism of lactate results in production of short-chain fatty acids: propionate and acetate as well as production of CO_2, which allows for the formation of typical eyes in the cheese. The main contribution of *P. freudenreichii* to flavour development is through the hydrolysis of lipids, release of amino acids, most notably proline, and volatile compounds [1].

P. freudenreichii preferentially uses lactic acid as a carbon source and of the two isomers, it is better suited for using L-lactate than the D-lactate form. The phenomenon has been attributed to accumulation of pyruvate during growth, which in turn has an inhibitory effect on the activity of D-lactate dehydrogenase [2]. In addition to that, our analysis revealed that while all the strains possess two homologues of L-lactate dehydrogenases (ldh and ldh2), only nine of the sequenced strains carry genes coding for D-lactate dehydrogenase (JS2, JS7, JS8, JS10, JS15, JS17, JS18, JS20, JS23). D-lactate dehydrogenases were located on genomic islands, suggesting that the ability to utilise D-lactate is strain dependent and acquired through horizontal gene transfer.

Aspartase activity is associated with higher ratios of acetic acid and CO_2 to propionate produced during the metabolism of lactate, and especially after exhaustion of lactate, which leads to higher accumulation of gas [3]. The majority of the *P. freudenreichii* strains possess two aspartase genes coding for aspartate ammonia lyases, which share about 87% amino acid sequence identity. The exceptions are strains JS4, JS9 and JS25 where only one gene is found, and strains JS8 and JS18 where the second aspartase gene is disrupted by a transposase. Additionally, in strains JS, JS11 and JS13, a transposase is inserted between the aspartase genes, but the genes appear intact. As a result of a 35-gene duplication, strain JS17 possesses three complete aspartase genes: PFR_JS17-1_710, its duplication PFR_JS17-1_745 and the third one, found in all of the tested strains, PFR_JS17-1_746. The presence of three aspartases could contribute to increased gas production by this strain, which is associated with split defect in the cheeses [4].

The lipolytic activity of *P. freudenreichii* in cheese, associated with the development of the typical flavour, has been tied to two esterases: one secreted into the environment and one surface anchored [5]. In the experiments with mutants lacking either of the esterases it was established that the activity of the secreted esterase accounts for 75% of lipolytic activity in the tested strain CIRM-BIA1 (equivalent to JS15 in this study) [5]. We performed a BLASTp search of the 20 *P. freudenreichii* proteomes and found that both esterases are present in all 20 strains, and that they are also very highly conserved. The only differences observed were that the 413 aa long surface-anchored esterase is 10 aa shorter in strain JS (PFREUDJS001_002032) and 13 aa shorter in strain JS18 (PFR_J18_822). The 444 aa-long secreted esterase is 47 aa shorter in strains JS11 (PFR_JS11_1809) and JS13 (PFR_JS13-1_1811), but it is also 43 aa longer in the strain JS9 (PFR_JS9-1_1970).

References:

1. Vorobjeva L. Economic and medical applications. In: Propionibacteria. Springer Netherlands; 1999. p. 209–43.
2. Crow VL. Utilization of lactate isomers by *Propionibacterium freudenreichii* subsp. *shermanii*: regulatory role for intracellular pyruvate. Appl. Environ. Microbiol . 1986;52:352–8.
3. Crow VL. Metabolism of aspartate by *Propionibacterium freudenreichii* subsp. shermanii: Effect on lactate fermentation. Appl. Environ. Microbiol. 1986. p. 359–65.
4. Daly DFM, McSweeney PLH, Sheehan JJ. Split defect and secondary fermentation in Swiss-type cheeses – A review. Dairy Sci. Technol. 2010;90:3–26.
5. Abeijon Mukdsi MC, Falentin H, Maillard MB, Chuat V, Beatriz Medina R, Parayre S, *et al.* The Secreted Esterase of *Propionibacterium freudenreichii* Has a Major Role in Cheese Lipolysis. Appl. Environ. Microbiol. 2014; 2:751-6.
