# Supplementary material for: De novo assembly of genomes from long sequence reads reveals uncharted territories of Propionibacterium freudenreichii
Source: BMC Genomics. 2017 Oct 16;18:790. doi: 10.1186/s12864-017-4165-9 (PMC5644110; doi:10.1186/s12864-017-4165-9)
Supplement: Supplementary file 24 — The CRISPR spacers within CRISPR arrays identified in P. freudenreichii. The collection of all CRISPR spacers predicted in the P. freudenreichii strains together with their predicted immunity. (PDF 75 kb) [file 12864_2017_4165_MOESM24_ESM.pdf]

The CRISPR spacers within CRISPR arrays identified in *P. freudenreichii*

Spacer sequence	Spacer ID	Immunity
CTGGGTAGTAGTATAATCTTACTTGTTGATGAAGGGG	JS_Crispr_2_1	-
CCGACGCTTGCGTGGAGCAGCTCGGCGACGTCCTTCTG	JS_Crispr_2_2	-
TGTTGGATGCCTGGCTGGTGGGGATCGTGCGCCACAGG	JS_Crispr_2_3	-
CCCCGAACTGGCGGTCCCGGCCGCCTCTTCTTCCGT	JS_Crispr_2_4	-
GCGAAGGGGATCAGTTTCCAGCCGGTCGAGGTCCTGACG	JS_Crispr_2_5	-
TGTAGCAACAGTGCTCCAGCATGTCAAGTGTGGGG	JS_Crispr_2_6	-
ACGCCCGGACGTGACATCCATCAGTTCTTCGACCCCG	JS_Crispr_2_7	-
CCTGTCATTCGCCCATTGGGCCGCTTGAGAAGCTTGAT	JS_Crispr_2_8	E1
GCTCGGGGATGCTGCCCTGGCGATGCTGCCGACCGC	JS_Crispr_2_9	-
GACAGCGCGGTCCGCTCGATCATCGGCGACCAGCCCT	JS_Crispr_2_10	G4,PFR1, PFR2
GACGATACAAGACTGACCTAACGAATAAGGAAACG	JS_Crispr_2_11	-
GCCTCCAGCGGTGGAAAGAGCGGCAGCGCTACGGACG	JS_Crispr_2_12	-
CCGACCGCCAAGATGATCAATGAGCGGAAGCACAC	JS_Crispr_2_13	PFR1, PFR2
TTGAAGCAGGCGCCCGCAGCGCCCCATCGCCCGG	JS_Crispr_2_14	-
GACCGGGTGAAGCGCGCCGGGGGCCTGAGCATCAAAC	JS_Crispr_2_15	-
GGATCGTAGCGGGGCTGTTGGCAGCAAGTCTCGCTG	JS_Crispr_2_16	-
GACTACAGGTGTTCGATCTGGTCTGTGGCCGTCGA	JS_Crispr_2_17	-
GTAGGTGGAGGGGCGCAGACATTAAAATCTACGCCC	JS_Crispr_2_18	-
CAGAACAACGCTCGAGGAAAGGGTTCCAATCTCATG	JS_Crispr_2_19	-
GGTGTTGAGTTACCGCCATATTCGGCCAGAGTCTTGAA	JS_Crispr_2_20	-
CCGTTTCGGCTGTCGCCACCAACTGCTCCGGGATGGGC	JS_Crispr_2_21	-
ACACCCGCCGCGATCGAGGCCGACCTGAAACGCTG	JS_Crispr_2_22	-
GACGGCGGGCCAGTTGCACAACACGGCCACCCGCACCAA	JS_Crispr_2_23	-
TTCGCGGTGCGGTCGGCAATGCTGGACGCGTGCCCGA	JS_Crispr_2_24	-
CTCACGCCCGGAACCCATGCCGGAAGATCAGTCGCC	JS_Crispr_2_25	-
GCGGCGCCTCAGATGGACCTGTCCAGCGGTGAGC	JS_Crispr_2_26	-
ACGGCCTCGCCGTCGCGGCTGATGCTCGCATGCATC	JS_Crispr_2_27	PFR1, PFR2
GCTCGGCGACGCCGCCCTGGCGATGCTGCCGACCG	JS_Crispr_2_28	-
GTGGTTTCGACGCCCTTGTCGGGGCCGTGCCTGCC	JS_Crispr_2_29	-
GCCGGTGCTCCCGTCACGCCGGGGCGTTCTTCGG	JS_Crispr_2_30	-
ACCTGGGCGGCCTTCGGATTGTCCAGGTCAAAGAAG	JS_Crispr_2_31	-
GGCGAGTTGGCTCGGACCTCTGGACAATTTTTGGA	JS_Crispr_2_32	-
GCAGCTGCTGGTCGCTTTGGTGCAGGGCATCCTCTC	JS_Crispr_2_33	-
TATTCACAGGATGCATGGGTTCGCTCACATATGGACG	JS_Crispr_2_34	-
CCGAGATCACAGATGCCGAGCTCATCACCATTCA	JS_Crispr_2_35	-
AGACGTGTCAGGCGACAGCGGAACCAAGACAGCCG	JS_Crispr_2_36	-
TTCGCCGTGAAGACCCGCGAGGCGGAGGTCGCCCGT	JS_Crispr_2_37	-
GGCGGGTCCGCGAGATTCGTCGGCGTCGCACGGCGG	JS_Crispr_2_38	Anatole, B22, B3, E1, E6

GTGAGCTCGGGCTGAGCGTGAGCCAGACAGCGGCAAAG	JS_Crispr_2_39	-
GCGAAAGAGCGATCATTCCGGGTATGGATCAAACC	JS_Crispr_2_40	-
GGGGTCTGCAACGGGGTCAGCCCAACAAGCTGACCCACTGGATCCACCACA	JS_PossibleCrispr_1_1	-
CTGGAGCTTGCCCTTGGCGGCCACTGATCCGAAGA	JS_PossibleCrispr_3_1	-
ATCCCCGACATTCGCGCCCACCTGAATCAAGCG	JS2_Crispr_3_1	-
GCCCTGATGAGCTCCAGATCATCAAGGTGTTCG	JS2_Crispr_3_2	-
ATCACGAGCGGCGTCACCATTGCCATCTGGATG	JS2_Crispr_3_3	-
TCGTGCCACAGGTAGGCGTCGCGGAGCTCGCCG	JS2_Crispr_3_4	-
CGCAGCACCGGCCACGACGCCTCAAGACCCACG	JS2_Crispr_3_5	-
GCCCTGATGAGCTCCAGATCATCAAGGTGTTCG	JS2_Crispr_3_6	-
CAAACTGTGGACACTTGCCCGCGACACTGACAG	JS2_Crispr_3_7	-
CCCTCGGCATTCTCGGAGGACTCGCGGGAGGCA	JS2_Crispr_3_8	-
AATCTCTATATTATTCGAATGAGTTTCATTATG	JS2_Crispr_3_9	-
CCAGCACTGTGATCCAGGTCTGGAAGTCATTCA	JS2_Crispr_3_10	B22, Doucette, E6, PFR1, PFR2
CTGGCGCTGAGGGACACCGACGCCCAATTGTTC	JS2_Crispr_3_11	-
TCGTTGCCCAGATGGACGGCCACCTCGATGCCG	JS2_Crispr_3_12	-
CTGATCGTCGCAGTCTCCGCACTGGTCGGATGG	JS2_Crispr_3_13	-
GACCATATAATGATTGATAATCTTATCATTAAG	JS2_Crispr_3_14	-
TGCCCGTTGCATTTGCGGTGTGCGTGGCGGAAG	JS2_Crispr_3_15	B3
CGGCGGGTTCGGTGTGGTCCCGCTCTTTTACGC	JS2_Crispr_3_16	-
GGTGGCTCGTGGAGCATCTCCGCCCAGGGGAGG	JS2_Crispr_3_17	-
CCACTTCGGGGTGACTTCCAGGCGACGCTCGAA	JS2_Crispr_3_18	-
GAACAAGCGTTTTCGGGACGGAAGAGAATAGCC	JS2_Crispr_3_19	-
GGTGCTGCGTGATCCGCGCCGGGACTCACTCGA	JS2_Crispr_3_20	-
GGCCTCGGGGCTCAGCGAGGCGTCCAGATCGGA	JS2_Crispr_3_21	-
CATGGGAACCTCCGCGTCACCATGGGTTGAACG	JS2_Crispr_3_22	-
CTTGGGTTCGCCGGTCTGCAGGTCGGTTTGTTG	JS2_Crispr_3_23	-
TTTATCATTACCGTCAAGCCGCTTAATCTCAGC	JS2_Crispr_3_24	-
GGAATAATATGACACGCACATCGTTGTACAGCA	JS2_Crispr_3_25	-
TGAGCCGAAGAAGCCGCTGCAAGATTAGAGGCC	JS2_Crispr_3_26	-
ACCCCAAAAAGGTCCTGCGATTTGTACCCCTGA	JS2_Crispr_3_27	-
TCGTCGGCGATGGCGTCGATGTCGAAGGCGGTG	JS2_Crispr_3_28	-
TCGTCGGCGATGGCGTCGATGTCGAAGGCGGTG	JS2_Crispr_3_29	-
CCCTTCTGTGGGAGATGCCCGGCGCCGACCAGA	JS2_Crispr_3_30	PFR1, PFR2
AAGTCACCCGGCGCCAGTTTGTTGATTGCATCG	JS2_Crispr_3_31	-
TGATGAATGCCATCACTTCAGCGCTCGGCCCGG	JS2_Crispr_3_32	-
AGCGTCCCGCGAGCCCCGTGAAGGGGCACATGC	JS2_Crispr_3_33	-
TGGCGCACATCCCTTCCTGTGTCGGTGTTGGTG	JS2_Crispr_3_34	-
CACAGGTCCCACTCCAGCGGCCGGCAGCCCGGG	JS2_Crispr_3_35	-
TGGCGCTTTCAATGTGCCGTATGCTGATTCGGG	JS2_Crispr_3_36	-

TTGGGCGGTCCGCTGGGGATCGCGGCGGCGGCG	JS2_Crispr_3_37	-
GGCGTGGCCGTCTGGAAGCTCGGGGCGAAGATG	JS2_Crispr_3_38	-
TCATTGCCCAGGTGGACGGCCACCTCGACGCCG	JS2_Crispr_3_39	-
CCAGAAAAGTTCCGCCGCGCTCAAGACTGGCAA	JS2_Crispr_3_40	-
CCCTTTCCGGGTCCACCGTGGCCGATTGTTTGG	JS2_Crispr_3_41	-
GCATCCGGGCAGACGCAGTTCAGTGTGGAGGTG	JS2_Crispr_3_42	-
GAGCGCGTGGGCCTGCCAGATGTCTGCACGGAA	JS2_Crispr_3_43	-
GGGCGCTGCTTATGTCGCTGTTCAGGACGCTGC	JS2_Crispr_3_44	-
TATTGTCGTGGCATTGAAGAATAAGGACGGACTG	JS2_Crispr_3_45	-
CCAGACATGACCAGCGAAGAGATCGCCGCCGAG	JS2_Crispr_3_46	-
TCTTCGGTGACGCGCCACCTGATCGAGGCCCGC	JS2_Crispr_3_47	Doucette
GCCTCAGCCATCGCATCCACCTGGGCGGCATCC	JS2_Crispr_3_48	Anatole, Doucette, E1, G4
CGGGGTGTGTGGCATGTGGCGCCGCCGGCGGCA	JS2_Crispr_3_49	PFR1, PFR2
CCGTGGCAGAAGGGGCCGTGCACGCCGAGGGGC	JS2_Crispr_3_50	-
TTGCCGCAGCTGATCTCTGCCGGCATTCAGCTG	JS2_Crispr_3_51	-
CCGTTCCGAAAACTGTCGGTGCCATGCCCTACA	JS2_Crispr_3_52	-
TGATGACGGCAGCATGATCGACGGCCAGCATCG	JS2_Crispr_3_53	PFR1, PFR2
CGGTGGGGCTTGATGTCATGCATGGGGGCCTCC	JS2_Crispr_3_54	-
TCGCCGAGCCGCTAGAGCTGTATTTGGTGGACG	JS2_Crispr_3_55	-
GGGGTGAGGATGTCGTCCCAGCTGGCCGTCGCG	JS2_Crispr_3_56	-
CTACGGAAATGCAGGCCCCGCGAATGTCTACAA	JS2_Crispr_3_57	-
CGGCCGATGTGGGCCAGCGTGGTGGTCAGCGAC	JS2_Crispr_3_58	PFR1, PFR2
TTTCGGGCCTGACAAATTTAGCGTTCCGACCGG	JS2_Crispr_3_59	-
GTGCGGATCTGGCTGCGGAATCCGACGGTGACC	JS2_Crispr_3_60	-
TGGTCCAGATGAAGAACGAGATCGCCGCGGCCC	JS2_Crispr_3_61	B3
TCCCATGTCGCCATCACAAAATCCTTTGATAGG	JS2_Crispr_3_62	-
TTTCGACGCTGCCGATTTGGCGGCCGGCCTGTA	JS2_Crispr_3_63	-
TTTCCCGGCCACTTCGGCATCCCACACGCGCAG	JS2_Crispr_3_64	-
TTGATCCGGCGAAGAGGGCGGGCGATGTGGGGA	JS2_Crispr_3_65	PFR1, PFR2
CTGGACGCCCCAGGGCAGTGGACGTAGCCAGCCG	JS2_Crispr_3_66	-
GGTCCTTTCAGGGTCTGGGCTCCATCGGGCTGC	JS2_Crispr_3_67	-
ATGATCCCGTTTATACGGCGATGGGGATATTCA	JS2_Crispr_3_68	-
TAATGTCTGCGCGCCTCCACCTACATGAGGAGA	JS2_Crispr_3_69	-
TCGCCGGAGGCATTGCGGGCGGTCCGGCGGCCA	JS2_Crispr_3_70	-
TTCGTCAAGGAGCTCAAGGCGTCGGGCCGGTCG	JS2_Crispr_3_71	-
CTCGGGCCGCCGTTCGGGACGCCGCCCGGGGCG	JS2_Crispr_3_72	-
TTCGCCAGATTGGCGATCTTCACCACGGGATCA	JS2_Crispr_3_73	PFR1, PFR2
CAGCGATGCCCGGTCGCTTCATAGTGATCCGGG	JS2_Crispr_3_74	-
TGGTGCAGCTTGTTCCGGTTGTCGCGGGAATCC	JS2_Crispr_3_75	G4, E6
TGTTTTCAGCGCTTCTACTCCTTCCACCTGAAA	JS2_Crispr_3_76	-
AATCGCGAAGTCGGCTCTGCTGTGGGCGGGGCC	JS2_Crispr_3_77	-

AGCTTCACGGCGGAGGATGCCATGGAGCAGGGG	JS2_Crispr_3_78	-
CTCAACGCTCTGGTCGACGAGTCGGGGGCAATC	JS2_Crispr_3_79	-
CTGGCCCGATCCTCGTCGGTGGCATCGGGATCG	JS2_Crispr_3_80	Doucette, B22
AGGGCGGTGCCCTTCCCCGAGGCGAGGGCGTCG	JS2_Crispr_3_81	-
GCGGACCTGTTGCGCCAGGGGTGTGTCGGCCAA	JS2_Crispr_3_82	-
GGATTCGCCACAGCACCAGCTGGTCGGTGTCAG	JS2_Crispr_3_83	-
CAACATCATGCGCGGCATGTCGCAGCTTGAACC	JS2_Crispr_3_84	-
TCGACCGCAGCATCCACAGTCTTGAGCACGATA	JS2_Crispr_3_85	PFR1, PFR2
TGGAGTGATGGCCTGGTTCAAGGTTGACGACCAA	JS2_Crispr_3_86	E6
TCCGGATTGGCGGGGGTGTTCACCACACCCGCA	JS2_Crispr_3_87	PFR1, PFR2
AACGCTGACGTGACCAAGCTGATGAACGTGCAC	JS2_Crispr_3_88	-
CGGCACCCAATATCGCGACTATTACGGCACTGA	JS2_Crispr_3_89	-
TTGGCATCGTGAACTCGCTGATCCAGGGGATCG	JS2_Crispr_3_90	B3
CGAGCCGGTCGAGGGCGGTGCGGCATGAGCACC	JS2_Crispr_3_91	Anatole, B22, B3, E1, G4
GTGCTCGGGGGTGCAATAGAAGCCGCTGCGGAA	JS2_Crispr_3_92	-
CACCCGGTCAAGGCGGTCTGCAGCGCCACTGCG	JS2_Crispr_3_93	-
GGACCCTTCAAATAGGGCGCGATGAAGATGAGC	JS2_Crispr_3_94	-
CGTATCTGGCACGGCGCACAATTTTCGCGGAGTC	JS2_Crispr_3_95	-
TGGTCGGCGCCGAGCCATTCCCGAAGGCCGTCCTG	JS2_Crispr_4_1	-
CGCGGCCTGCAGCACTGTAACTCCGTGTGGGCTTGCC	JS2_Crispr_4_2	-
TCCAAGAGCTTCAGATACTTCTCGTTCGTGACGTTT	JS2_Crispr_4_3	-
CGACATTCGTGCTATGGTGTCATTCGTCGGGCGCGC	JS2_Crispr_4_4	PFR1, PFR2
GACTGAATGGCGTCGAAATCCTCGAACATCTCGT	JS2_Crispr_4_5	PFR1, PFR2
CACACCGTTAATCGTCAGAATACGTACAAGATGACG	JS2_Crispr_4_6	-
GCCGAGCGCGTGGCAGCATATCAGCTGATCTCGGATGCCAA	JS2_Crispr_4_7	-
GCCTTTTTGGGACAGTTTGGTGTTAGATTATGAAG	JS2_Crispr_4_8	-
TGTCTTGAATCTCGAGCTCGGAGCCGATATCAAGCCCA	JS2_Crispr_4_9	-
GCTCGGTGACGCCGCCCTGGCGATGCTGCCGACCG	JS2_Crispr_4_10	-
GGGGCTGACGTTCTCCGTTGACGGCCAGCACAAA	JS2_Crispr_4_11	-
GTGTTTGAACCGGTCAGACGGGTCATCACCTTGAC	JS2_Crispr_4_12	-
AAGAATGGCGCGACCGTACGCCGCAACGGGCTGGG	JS2_Crispr_4_13	-
ATTTATGTTTTTTCTCTGGTGATTTCTGCCGCCGCGA	JS2_Crispr_4_14	-
GAGAGTGATTAGGACCCCAGCTGTGTAGCGGGGCCGCTC	JS2_Crispr_4_15	-
CGTTTCAAGTCTGAGACTTGACCCACGGTGTGGGTCGA	JS2_Crispr_4_16	-
CTGAAGGGGAGTACAGACATTGGTAAGCTCCTTATG	JS2_Crispr_4_17	-
GCAGGAACAGACTCGCGCTTGGTATCCAATCGACCA	JS2_Crispr_4_18	-
GCTTCCGTTGGTGAACACCATTTTGGTTTGATTCCACTC	JS2_Crispr_4_19	-
TCGTCGTGGGCGCCGGGCACGTCCATCTATCTGGCAAA	JS2_Crispr_4_20	-
TCGGCATGATGCCAGCGCCGATTATCGTGAGCGTATT	JS2_Crispr_4_21	-
AAGATGAGCGGGCCGAGGGGTTCGACCCGGGACGCGG	JS2_Crispr_4_22	-

GTGAGCTCGGGCTGAGCGTGAGCCAGACAGCGGCAAAG	JS2_Crispr_4_23	-
GCATTTGCTTGCCCATTTGCTTCCTCCAATCTGCGT	JS2_Crispr_4_24	-
CGCACCAAGGCCCCGGCTCGGCGAGGGACGGTTGCCGT	JS2_Crispr_4_25	-
CGCACCACCCGCAAGGCGACG	JS2_PossibleCrispr_1_1	-
AAGCCGGCAGCGAAGAAGCCAGCA	JS2_PossibleCrispr_1_2	-
GGGGTCTGCAACGGGGTCAGCCCAACAAGCTGACCCACTGGATCCACCACA	JS2_PossibleCrispr_2_1	-
CTGGAGCTTGCCCTTGGCGGCCACTGATCCGAAGA	JS2_PossibleCrispr_5_1	-
GGTTTGATCCATACCCGGAATGATCGCTCTTTCGC	JS4_Crispr_1_1	-
AATACGCTCACGATAATCGGCGCTGGCATCATGCCGA	JS4_Crispr_1_2	-
CACCCGCAGAGGCAGCACCAGGCCGAAGCGCGCCGACAT	JS4_Crispr_1_3	-
TTTTGCCAGATAGATGGACGTGCCGGCGTCCACGACGA	JS4_Crispr_1_4	-
GAGTGGAATCAAACCAAAATGGTGTTCACCAACGGAAGC	JS4_Crispr_1_5	-
CTGTCGAAATGGCGTGAGGTGAGCGGCGGTGCAGG	JS4_Crispr_1_6	-
TGGCACCTGCTCGATAAATAAGCTCGATCAGGCCCTG	JS4_Crispr_1_7	-
CCGATCCTGTGGGCCGAGGCCGGACCTGAAGCCT	JS4_Crispr_1_8	-
TGCGTAGGGTGCGGTCCTCTGCGATCTGATCGGGCT	JS4_Crispr_2_9	-
TCGTCGGTGATCAGGTGCAGCGAATCGGTGTCGCA	JS4_Crispr_2_10	-
ACCGAGAACGTTCAGCAACGGCCAGCCGATGATGAC	JS4_Crispr_2_11	-
TGCCGTGTGGGGCGGTGGTGGTGCTGCCTCCCCAAT	JS4_Crispr_2_12	-
AACCACAGCTACGTCTTCCGGTGTCGGCGCGGATA	JS4_Crispr_2_13	-
TCCATGTGCCAATGTCAATGTGAGCGACATCGCG	JS4_Crispr_2_14	-
CAGAACAACGCTGGAGGAAAGTGTCCCAATCTCATG	JS4_Crispr_2_15	-
GCGAATGTCAGGGATCGGGTCTTGAACGCCACCGAT	JS4_Crispr_3_1	-
CAGCCCGCAACCGCTCGACCTCACTCGACGCCAT	JS4_Crispr_3_2	-
ACGAGCCAGCGAATACCACTGGCCGCGATTGGGGTTGAT	JS4_Crispr_3_3	-
GGCCTGCGCCTGGGGGTCGCCCCCGCGCATGAAGTG	JS4_Crispr_3_4	-
ACCTGGACAGTGCGGGTGATCTTGCCACCCAGCTT	JS4_Crispr_3_5	-
ATGCGCGCATTGGCGCAGAACTTGAAATGCTTGGACGT	JS4_Crispr_3_6	PFR1, PFR2
AGGCGCCTGTGGTGACACCGCGATCACGAGCCAGCGA	JS4_Crispr_3_7	-
CGACCTCACTCGACGCCATGCACACACCCCACAG	JS4_Crispr_3_8	-
AACGGCAAGGAGCAGATCCTCGCCAAGAACTTGGCGCA	JS4_Crispr_3_9	-
TCGAACGCTTACTCCTGGTTTCCACGGGAACCTCGCG	JS4_Crispr_3_10	-
TTTGCTTACTCAGGGCTTGTCTCTTTTGGTCGTTTTGC	JS4_Crispr_3_11	-
TCCCCGCACGATCGCCGGAATGGTCACCGCTTCGGATGC	JS4_Crispr_3_12	PFR1, PFR2
TTATGGCGCTGCTTGGGACGCCGCTCAGATCGCCGCT	JS4_Crispr_3_13	-
CGTTCTCGTGCTGAGCTACGGCATACGGGGTGTC	JS4_Crispr_3_14	-
AGTCGGGTGTAATCATTGACCTCCGCACCCGGCAAGCA	JS4_Crispr_3_15	-
CGTCCATGTGTGAGCGAACCCACGCATCCTGACT	JS4_Crispr_3_16	-
ACTTCGAGGATGCCGGCGAGGAAGCCGGTAGGAGT	JS4_Crispr_3_17	-
GCGATCTCGGCGCTGGTGGGGGCGTTCTCGATTGC	JS4_Crispr_3_18	Doucette, G4
CAGATGAGCGAACTGGATTTTTACCAATTGCTAGATG	JS4_Crispr_3_19	-

GCGAGCCAATCGACATCGCCCGGAAGGTTAGCGAGAAA	JS4_Crispr_3_20	Doucette, E6
CGGCATCATGTGATCACCCGACCCATGACCTGCTCCCACG	JS4_Crispr_3_21	-
CGTTACTCATTGTGTTTCTCCTAGAACTTTAACGT	JS4_Crispr_3_22	-
GGTGAGAATCCCGTCGCCCGTACCGGGCGCGAGCGTGA	JS4_Crispr_3_23	PFR1, PFR2
GGTCATAGGTCGGATTATATCCGCCCCATGTGATTAAA	JS4_Crispr_3_24	-
CTTGATATTGGGTCTGAGCTTGAGATTCAAGATATCAA	JS4_Crispr_3_25	-
CTGTGCAGTCCGTCTCCACCGTGGAAGCCGACAA	JS4_Crispr_3_26	-
AATCCGGACCAGTAATGTCAGGAATCTTGATCTT	JS4_Crispr_3_27	-
CACGCGCTCAGGGTGAGGGCCCGTGGGATGGCTGGGT	JS4_Crispr_3_28	-
CATTACTCATCGTGTTTCTCCCTAGAACTTTAGTGT	JS4_Crispr_3_29	-
CGGCTCGTTCACCGCATCCTTCGGGGGTGTCGGGGCAG	JS4_Crispr_3_30	-
GGCAGCCGCGATGGCTCTGGTTGTCTTGCGCATGCAA	JS4_Crispr_3_31	-
TTCAAGAATTTTGTTGAAACTTTTGTCGATTTCTACA	JS4_Crispr_3_32	-
CCGAATCGACGTAGACAAGCCAGGTCTTCTTTCCGG	JS4_Crispr_3_33	-
ACGCCCCCAGACAAAGCCGAACCTAGCGGAACTTGACC	JS4_Crispr_3_34	-
ATTGCCATTCGCTGCATCCCGCCAAGGGTGTCGGTA	JS4_Crispr_3_35	E6
TGCCGGCTTGGCCGTGGACGCCGT	JS4_PossibleCrispr_4_1	-
TGCCGGCTTCTTGGCCGCCGGCTT	JS4_PossibleCrispr_4_2	-
CGTCGCCTTGCGGGTGGTGCG	JS4_PossibleCrispr_4_3	-
TCAAGGAGCGGCGTGGAGCCCGCCTTCGCTCCAG	JS4_PossibleCrispr_5_1	-
ATCCCCGACATTCGCGCCCACCTGAATCAAGCG	JS7_1_Crispr_2_1	-
GGGGACGAGCGCGGTCGCCTGCGCAACCTTGGC	JS7_1_Crispr_2_2	-
ACCGACGAAAATAATTCGGAAGTTCCTATTATC	JS7_1_Crispr_2_3	-
TGGCTCGTCAAGGACCTGATCTCCGTCGAGGAC	JS7_1_Crispr_2_4	Anatole, B22, B3, E1, E6, G4
CCTACAAGCCGCGCGCCGGTGACTGGGCTGTGC	JS7_1_Crispr_2_5	-
AGATTCAGCGAGTCAATCGCGTCGTGGGCGGCC	JS7_1_Crispr_2_6	Anatole, B22, B3, E1
ATGCGGGGCTGCACCTGCTGGCGTTGCAGGCCC	JS7_1_Crispr_2_7	B22, G4, PFR1, PFR2;PJS7
TGCGATAGGGCTCCAGGCTGAGCGCCGCCGGAG	JS7_1_Crispr_2_8	-
GCTGAATGGTGATTTCATCACTTTGCCTCCAAG	JS7_1_Crispr_2_9	-
TCGGCGTGCCGGGCTCCACGCTCACGGGCACGC	JS7_1_Crispr_2_10	E6
CGCGTAGACGTCGGGCGGCAGGGCCTCCTTGGCC	JS7_1_Crispr_2_11	-
CAGTCAACAGTAATTTCAGTCCCCCAGCGATCG	JS7_1_Crispr_2_12	-
CCACGATGAACACCGAGATCGCCGTCGAGATCG	JS7_1_Crispr_2_13	-
CCGGGTCGGGCGACCCTGCTGGGTCGGGCGGCG	JS7_1_Crispr_2_14	-
CCTGTCCGTCCTGCCACGTGTGTTGTGGCATTG	JS7_1_Crispr_2_15	-
CTATGAATATTAATCCCAAGGATTTGCTACTCA	JS7_1_Crispr_2_16	-
TCGGAAGGTCCGTTCGGGCGATTCGAGCCGCCC	JS7_1_Crispr_2_17	-
TTACTTGAAAGCCACGAACTGAACCCAGAATAA	JS7_1_Crispr_2_18	-

TCGGAGGAAGGCAACAGGCTAAATCCTGATCTT	JS7_1_Crispr_2_19	-
GGGCGGATAGCCGTCGGCGTAATCGCTGGTCCA	JS7_1_Crispr_2_20	Anatole, B3, Doucette, E1
CATGGCGGACGGTGTTGGTGGATGCCGACTGGG	JS7_1_Crispr_2_21	Anatole, B3, E1
CGCCGGTCGGGATGGTTTCGAGGTCGCGGACTG	JS7_1_Crispr_2_22	-
TCTCGGTTCGCCGGTCTGCAGGTCGGTTTGTTG	JS7_1_Crispr_2_23	-
GTCAACTTGTCGCCGCCGGTGATCAGTTTGACC	JS7_1_Crispr_2_24	-
GGCGGGGACCCCAGCGTCGTCCTGGACGAATAC	JS7_1_Crispr_2_25	-
CACCCCCACCGTCGCGGAGCGCCACACGATCGA	JS7_1_Crispr_2_26	-
GCAAGGGCTGCTGGAGACGGTCGCCTTCGCGAA	JS7_1_Crispr_2_27	Anatole, B3, E1
CGGAACATCGTCATCGGCTGGCCGTTGCTGAACG	JS7_1_Crispr_2_28	-
GGGCTCCATCGGGCTGCTTCCCGGTGGGGCCTC	JS7_1_Crispr_2_29	-
ACCCCAATCGCGGCCAGTGGTATTCGCTGGCTC	JS7_1_Crispr_2_30	-
GGTTTTTCATGGCTGGAATCCTTCCGACCCGAC	JS7_1_Crispr_2_31	-
CCGGTCATGATGCCTCCGAACTTGTAGACGTTG	JS7_1_Crispr_2_32	-
CATCCAGAAGCTGTTGCCCATCTTCGCGGACAC	JS7_1_Crispr_2_33	B22
CATCGGGCTGCTTCCCGGTGGGGCCCTTCTTGC	JS7_1_Crispr_2_34	-
CACGCCGACCAGGGTCACGGCCAGCGCCGCCCA	JS7_1_Crispr_2_35	-
CCCTGATGTGATGGTGCCGGCCACGCCCGCTGG	JS7_1_Crispr_2_36	-
CACCCAGTTCGAAACCGAAACGCCGTACAGCAAG	JS7_1_Crispr_2_37	-
CGACTCGTCTGCCAGGATCCGTGCACAGATCGA	JS7_1_Crispr_2_38	-
ACATCCCACCAGTCGGTGTCTGAGGTCGGAACG	JS7_1_Crispr_2_39	-
CTCGACGGCGATCGAGCCGCTTGTCGTCGGCGG	JS7_1_Crispr_2_40	-
TCGTAGGCCTGTCGCAGTTCCGAGGTCTTGACC	JS7_1_Crispr_2_41	-
TGTGGTCAAGTTCGGCAGCGACGCGGTGAATTCC	JS7_1_Crispr_2_42	-
ACAAGACCCTTGGCCAGATCACCAGTGGCGTTG	JS7_1_Crispr_2_43	-
ACCGGACCCCAGCCCGCCAGATTGTGGGGGCGC	JS7_1_Crispr_2_44	-
CGACTGTCGGAAACGTGATCGCCCTGGCGATCC	JS7_1_Crispr_2_45	-
CGACTTCCCGCAAAGGTCAGCCTGTCTCCGAGC	JS7_1_Crispr_2_46	-
TTAGAGTTTGTCGTGACAGCTACGTGGACCCAG	JS7_1_Crispr_2_47	-
CCAAGCCCGAAGGCGGTGCCCTTCACCGCCGCA	JS7_1_Crispr_2_48	-
TCGGGTCAGCGATCGCCGCTCCGTTTTCTGGGA	JS7_1_Crispr_2_49	-
TTTCCCGGCCACTTCGGCATCCCACACGCGCAG	JS7_1_Crispr_2_50	-
CATGGATCCCTGTGCGCGAATTCAGGCTTGGTC	JS7_1_Crispr_2_51	-
CTGAATGCCCCAAGGGGACCTCGATCCCGGTCC	JS7_1_Crispr_2_52	-
CCCTTGATCTCGGTGTCCACATCGTCGAGGGCC	JS7_1_Crispr_2_53	PFR1, PFR2; PJS7
CGCCGATGCCGGAATCACCAACGTCTCGACCCAG	JS7_1_Crispr_2_54	-
AGATCGGCGGCGATCTGGGCGCCCGTGGCGATC	JS7_1_Crispr_2_55	-
CAAGAAGAGCGCGACCGGCTGGCGGCCATTCCG	JS7_1_Crispr_2_56	-
TGCTCCGCATTTGATCTTGTCAGGCTCCACAGA	JS7_1_Crispr_2_57	-

GCGGTCTGCGTCTCACCCTTCTTGTAGGCCCAA	JS7_1_Crispr_2_58	-
TCACCGGGCTGCATCCACACCGGCGGCCACCCAG	JS7_1_Crispr_2_59	PFR1,PFR2 ;PJS7
TTGTCCGGTTAGACCAGACGGGAATGTTCAATC	JS7_1_Crispr_2_60	-
ATCGTCCCGACACCGCCGATTTCGGGGTCAAGG	JS7_1_Crispr_2_61	-
CCCCCGGTGGGCATCCAGAAGCTGGGACGAACG	JS7_1_Crispr_2_62	-
TTCCTCCTGCCCCCGCTGGTGTTGCTGTGGCTG	JS7_1_Crispr_2_63	-
CGTGTCATGCAGACCAATGTGGCTGCACGTGCG	JS7_1_Crispr_2_64	-
TTGTCTTTCTCGTCTGACTGTTGTTGCAGTGTC	JS7_1_Crispr_2_65	-
CGCACCACCCGCAAGGCGACG	JS7_1_PossibleCrispr_1_1	-
AAGCCGGCGGCCAAGAAGCCGGCA	JS7_1_PossibleCrispr_1_2	-
ACGGCGTCCACGGCCAAGCCGGCA	JS7_1_PossibleCrispr_1_3	-
CTGGCCAGAAACCTCTACCCCGAGCCCCTTGA	JS7_1_PossibleCrispr_3_1	-
CTGGCCAGAAACCTCTACCCCGAGCCCCTTGA	JS7_1_PossibleCrispr_4_1	-
CCGCGGACATAGACCGGCCCCATTCCGAAGCCCACA	JS8_Crispr_3_1	-
ACAGAAACGGTCTTGGTGCATGTTGCTGTCCTCTC	JS8_Crispr_3_2	-
CTAAAACGGGACTGCTTGGCTGGTGATGATGAAT	JS8_Crispr_3_3	G4, PFR1, PFR2
ATGTCATCTGCCACCGCACCTGCTGGGCAATCGA	JS8_Crispr_3_4	-
AGTGTTACGTGTTGAAGGGGTCCAGCCCTTCACGAA	JS8_Crispr_3_5	-
TTCTCAAAGGCCGCTTTTGAGCTGACTTTTCAGGGGGCG	JS8_Crispr_3_6	-
CCGCCGCCCTTCACACGGGTGGCCGGTGTGTGGTTAAA	JS8_Crispr_3_7	-
TTCCACGTCCAGGCTCTGTGCCGCGAGCATTTCCAGCT	JS8_Crispr_3_8	phiB5
CGCTCGTGCGACCGCTGCGGGAAGCCCATTTTCGACG	JS8_Crispr_3_9	-
TCACCGCGCTCTGACAAGGCCCGATACTTTCGCCCG	JS8_Crispr_3_10	B22, Doucette, E6
GGCGTGCAGCAGTCACTGCGCAAGTGGGGGCGTCGGGA	JS8_Crispr_3_11	Anatole, B3, Doucette, E1, E6
CGCGAGCGAGCCAAGGCCGAGGCCGACCGCTTCACAAA	JS8_Crispr_3_12	-
TGTCGGTCATGATCCTGGTCCTTTCAGGGTCTGGGCT	JS8_Crispr_3_13	-
CTTCTCCACCCGCGGCCAGGCCATCGCCTCCATCAC	JS8_Crispr_3_14	-
TTTGTGTGGCACCGTGTCGGACACCAGCGTCCGGCA	JS8_Crispr_3_15	-
CGAGCTCTCGTCCCTGCAGCTCGACGCGCTGGTCAA	JS8_Crispr_3_16	-
AGGTGCCACATCAGCCAGAAGCCCAGCAGCCGCTGCA	JS8_Crispr_3_17	-
TCGGCGCGGGCATTGGTGACTGCACCTCCGGCCTCGAT	JS8_Crispr_3_18	-
TCCCATGTCATCAGAGGATCCTTTGGTAGGACAGGGT	JS8_Crispr_3_19	-
TTGCCGACCAGCCCGGCGACCACGGTATTCACGA	JS8_Crispr_3_20	PFR1, PFR2
TTCACCTGAAGCCCGAGGCGCTCCCGGATCCGGATC	JS8_Crispr_3_21	-
TCTGAACCGCCGATAAGCACGGCCAGCCACCCGGA	JS8_Crispr_3_22	-
GAGCTGATCGGCAAGGTCCCGACGCCCCCACTTGCG	JS8_Crispr_3_23	Anatole, B3, Doucette, E1, E6

GACGCCGCCGAAGACCGCGCAATCGACATCCTCCAC	JS8_Crispr_3_24	-
ACTCCTTCTCGATAGTTCCTTGTCCGGTCGGCCCTC	JS8_Crispr_3_25	-
TTATCCGTTTCGACTGTCGCCACCGACTGCACCGGGAT	JS8_Crispr_3_26	-
CGCGTCCGGATGCCGGGGTCACCCGTCAAACCAA	JS8_Crispr_3_27	-
TCCCACCACGCTGGCGATCCAGCCTTCGACCATGCA	JS8_Crispr_3_28	-
TAATCTCCTCTGTAGGTGGAGGGACGCAGATCATA	JS8_Crispr_3_29	-
CGGTGAGCTCCCTGACCCGCTAGATTTCTGTGAGC	JS8_Crispr_3_30	-
GTGGGCGACGGGGTCGCGAAGAACCGTGGCTTCAAC	JS8_Crispr_3_31	-
GCTGATCATCCAGCTGGTCGGCGGGATTGTCGGG	JS8_Crispr_3_32	-
CAGGCGATCCGTCTGTCGAAGGACTCGAACGGCCA	JS8_Crispr_3_33	-
GGTGGTAATTCCGATTGGTTTCCACCGTTTGATAA	JS8_Crispr_3_34	-
GACATGGATGGCGTGATCCGTGACTATGTGGCAGC	JS8_Crispr_3_35	-
AATTCTACGGAGAACAGCAAGAATAATGGGTCGCT	JS8_Crispr_3_36	-
GGGAGGGCGAGGCTCGAAGCGATGACAGCACTCGCCT	JS8_Crispr_3_37	-
CCTGTTTTCGGGGCGCTTGAGGGCACGACGACTTTCGA	JS8_Crispr_3_38	-
AGGCGGAAGCGCCACCATGCGACCCCCGTCACCCCG	JS8_Crispr_3_39	-
AGGTCGCAGCTGCCGGTGATCTTGCCGAGGGTCTTGG	JS8_Crispr_3_40	-
GAGCGTTGCCAGGCGGTGAAGGCTTTGTTGTCGCCGT	JS8_Crispr_3_41	-
GTCGAATCAGGGAAGTAGGCGTTCGTTGCCGTTCAT	JS8_Crispr_3_42	-
GTGCCATGTGGACCCCTCACCCCGACAAATTTCCTC	JS8_Crispr_3_43	E1, Anatole, B3
CGGGCCTGCTGGGTGTCGGGGTCGGGGTCGTCCGACGG	JS8_Crispr_3_44	-
GCCAACCAGCCGGGATAGGCCCGAAAAACGACCCAGG	JS8_Crispr_3_45	-
GCCAACCAGCCGGGATAGGCCCGAAAAACGACCCAGG	JS8_Crispr_3_46	-
GACGACTCAAAACTTGCGCACCCACTGGATCACCT	JS8_Crispr_3_47	-
GTCGGAGAGGTGTCCGGTGACCAGGTGACAACCGA	JS8_Crispr_3_48	-
GCTTCCGTTGGTGAACACCATTTTGGTTTGATTCCACTC	JS8_Crispr_3_49	-
TCGTCGTGGGCGCCGGGCACGTCCATCTATCTGGCAAA	JS8_Crispr_3_50	-
AAGATGAGCGGGCCGAGGGGTTCGACCCGGGACGCGG	JS8_Crispr_3_51	-
TGATGCCCCAGGCCGGCGCGCAACTGCTCACCGAGCTG	JS8_Crispr_3_52	-
AGGCGCCAAGGGAATGCGCTTCTACAACCGCGTCACGCTG	JS8_Crispr_3_53	-
GTGAGCTCGGGCTGAGCGTGAGCCAGACAGCGGCAAAG	JS8_Crispr_3_54	-
AACCTGTGGGGCTTCCCGGTTGTCGTCACGACCGC	JS8_Crispr_3_55	-
CGCACCAAGGCCCCGGCTCGGCGAGGGACGGTTGCCGT	JS8_Crispr_3_56	-
GTCATGGTGTGCGCACAGAAACCTAGAGTACGGGCGG	JS8_Crispr_3_57	-
GCGAAAGAGCGATCATTCCGGGTATGGATCAAACC	JS8_Crispr_3_58	-
CTGGGGGTAGCCGGGCTGGCCTTGGGGGTAGGTGCC	JS8_PossibleCrispr_1_1	-
GGTGAGCTACCACGCCAGCCGCCTGGCCTGGGCGCCG	JS8_PossibleCrispr_2_1	-
CTGGCCAGAAACCTCTACCCCGAGCCCCTTGA	JS8_PossibleCrispr_4_1	-
ACTGCACCGTCGGATCAGCCGGATAACCATTCACCGGCCCCGAA	JS8_PossibleCrispr_5_1	-
TCCTCAAAGCGGAGATAAGCATCCTTGTCGAGC	JS9_1_Crispr_2_1	-
CCACCCAGGGGAGGTTGGCAGCTGGGATGCAGC	JS9_1_Crispr_2_2	-
TCCAAATCGGCGCGCTTCTCCACATCGCTCCAG	JS9_1_Crispr_2_3	-

GCAGCGAGTGCCGAGCTGCACCAGCTCGCCGCA	JS9_1_Crispr_2_4	-
GACATCTGCGAGGCGGGTGTTCAGTGCCGCCTG	JS9_1_Crispr_2_5	-
AACCTGTCCGAGCAGCGTCTCAATCTCGCGGCC	JS9_1_Crispr_2_6	-
GCAACGTCCTGATAGGCCTTGCTGGCGAGGTTG	JS9_1_Crispr_2_7	-
CTTGCTGCTGCCTCGATAGCTTCCGCCGCGACC	JS9_1_Crispr_2_8	PFR1, PFR2
CGGCTCCGAGGATCCGCTTCGGCGTGTTGGTGG	JS9_1_Crispr_2_9	-
TTCTCACGATCTTCAGCGGCCTTCTGCTTAGCG	JS9_1_Crispr_2_10	-
TAGACCTGCCCATTGCCGGTGACCTTCGGCGTC	JS9_1_Crispr_2_11	-
ACCATTTCCGCAAGTGCCTCAAGGGTGACGTCC	JS9_1_Crispr_2_12	-
ATTGTAGCCGGCTGGCTGATTTTGAGTTGACGA	JS9_1_Crispr_2_13	-
CTCGTATTGCGCATGACGTGGGTCCTTGAATCG	JS9_1_Crispr_2_14	-
TTGAAGGCCAAGGCATTCCCTTGGAGCGACTTG	JS9_1_Crispr_2_15	-
TTAGTGCATTGCGGGCAGATGTCGCGCTTGGCG	JS9_1_Crispr_2_16	-
TTCGGAGGCGATTCGCTGGCCGTGCTGCGTGAC	JS9_1_Crispr_2_17	PFR1, PFR2, G4
TTCGAAAAAAGATGGCCCCAACACTTGACATG	JS9_1_Crispr_2_18	-
CGAGAACTCGAAGACCTTGAGCTTGCGCAGCTCG	JS9_1_Crispr_2_19	-
CATGCGGGCATGACGGCCATCAAGCCGGTGAGC	JS9_1_Crispr_2_20	-
GGCGAGCAGGCCACGAGAAACCTGTGCACGCGG	JS9_1_Crispr_2_21	B3
AGGCGGCCCAGTGGGTCGGTGAGGCAGATGTCG	JS9_1_Crispr_2_22	B22
GGACGCTGCTCTGGGCGCTGCTCGGGGCGCTGC	JS9_1_Crispr_2_23	-
CATCCGGCTCAATCAGGTGAGCCTCCGACAAAC	JS9_1_Crispr_2_24	-
TTGAGACGGCCAAGAACGAGATCAACACCTCTG	JS9_1_Crispr_2_25	-
GCATCAAGAATATCCGGAAACCGCCCTTTTCAG	JS9_1_Crispr_2_26	-
CGTCGTTGATGCGGGCTATCGGGGTTTGCAGCA	JS9_1_Crispr_2_27	-
TTTGGGGTGGATGGTCGTCTGCCCTGGCTGCGG	JS9_1_Crispr_2_28	phiB5
GTTGTGGGTGCTCTGGTGGTCGTGGGTGTCGGG	JS9_1_Crispr_2_29	phiB5
GTTGAATAGAACTCAAAGAATGTTTGATCCTCA	JS9_1_Crispr_2_30	-
TTGTCCGGCTCGACGAGACGGGAATGTTCAATC	JS9_1_Crispr_2_31	-
GGCGACTTCATGGTGATCTCCTCTCAGCCGTTG	JS9_1_Crispr_2_32	-
CGTTGTGGGTGCTCTGGTGGTCGTGGGTGTCGGGG	JS9_1_Crispr_2_33	phiB5
CTGCCGCAGCTGATCTCCGCCGGCATCCAGCTG	JS9_1_Crispr_2_34	B3
TACCACATTTCTAGGCAGAGGCCGGGGGCGTCA	JS9_1_Crispr_2_35	-
GAGGCTGGTCGTCCGGTGCTTTCAACGGTGCGG	JS9_1_Crispr_2_36	-
TTCGGGGGTGTGCCGTGGATGACATCACCGAGC	JS9_1_Crispr_2_37	-
GAGTTGCGGAAGCTGACTGTCTTTGAGTTCTCG	JS9_1_Crispr_2_38	-
ATTGAGCATGTCCATGATTCGTGTCCTCACTTG	JS9_1_Crispr_2_39	-
CGCTTCAACGGAGTGGCGAGCGTCGCCGGTTCC	JS9_1_Crispr_2_40	-
CTCTGGACACCGCGTTGGACACCGTTTGGGACG	JS9_1_Crispr_2_41	-
TGGCTCGTCAAGGACCTGATCTCCGTCGAGGAC	JS9_1_Crispr_2_42	Anatole, B22, B3, E1, E6, G4
ACAGCCGAAACCGACAGGGAGGTGCGGGCATCG	JS9_1_Crispr_2_43	-

GTCCCAGTCGGTCAACGGCGCTTCGGTGAATTA	JS9_1_Crispr_2_44	-
CTTCCCGTGGACCGTCGACGCGCTATCCATATG	JS9_1_Crispr_2_45	-
TCGTCGGCGTGCTCGATGCCGATCGAGGTCAGG	JS9_1_Crispr_2_46	Anatole, B3, E1
TGCTCGATGCCCTCCACGTGGATCGGTCCGTCC	JS9_1_Crispr_2_47	-
CCCGTAAACCAATCAATGACTGGTTGGATGATG	JS9_1_Crispr_2_48	-
AGGCCACGGAGTTCGCCATGGCCGAGGTGATCG	JS9_1_Crispr_2_49	B22, Doucette, E6, G4
TCCCGGCGGAGACATCACCATCGCGCTGCAGGAC	JS9_1_Crispr_2_50	-
GCCCTCGGCGCGACGCTGATCGGCGTCCCACTG	JS9_1_Crispr_2_51	-
CAGTCGGGCAGCTCGGTGGCGTCGAGCCATTCC	JS9_1_Crispr_2_52	-
CGGCCGATGTGGGCCAGCGTGGTGGTCAGCGAC	JS9_1_Crispr_2_53	PFR1, PFR2
CAGTGGCCACGGACCATGAATCGCACGGTGAGC	JS9_1_Crispr_2_54	-
GTTTGGCAACCGCGCAAGCAACGTCGGGGCCGT	JS9_1_Crispr_2_55	-
CATGAATCGCACGGTGAGCTTGTGCCCGGTGCC	JS9_1_Crispr_2_56	-
TTAGTCAACAAGTACAACGACATTGCCGGGATG	JS9_1_Crispr_2_57	-
GCTCGGTTCTTTGGCCCGCCGTAGAAGACGGCA	JS9_1_Crispr_2_58	-
TGGGTCGCCGCCTTGATAGCTTCCCCCGCAGCC	JS9_1_Crispr_2_59	-
TATGTCTTCCGGTGTCGGCGTGGCTATTACGCG	JS9_1_Crispr_2_60	Anatole, B3, E1
CGGCGATGGCTGAGCTCGAGGCCCGCGACCGTA	JS9_1_Crispr_2_61	-
TCGGAGAAATAGACCGAGTCGGTCTCCGCGTAA	JS9_1_Crispr_2_62	-
CGCACGAAGCACTCCCCAAGCGTGGCGTCATCC	JS9_1_Crispr_2_63	-
CGCAAAGGCGCGGCACCGGGGCAGGTGTTCAGC	JS9_1_Crispr_2_64	-
TAGGGGTAGTGGCTGAGAAGCACCACGACACCA	JS9_1_Crispr_2_65	-
CGTGTGCCTGCCGGTGCGAGCGATTCGTCGAGC	JS9_1_Crispr_2_66	Anatole, B3, E1
ATCACCGAATTTGGCCTTGGCGTCGGCCGCAGC	JS9_1_Crispr_2_67	-
CGCCTCCGCGAGACCGTCGATCTTGCCCGATAG	JS9_1_Crispr_2_68	-
TCAGCCACAAGAGTCCGCTGCAGCTGCTTGTAC	JS9_1_Crispr_2_69	-
GAAGGTTGGCGGTGGTGGGGATTGCGATATCTG	JS9_1_Crispr_2_70	-
GTCTTCCCAGATCGGATAGCGCCTTCCCAGAGG	JS9_1_Crispr_2_71	-
TCTGGCCGAACATCCTCCGGAAGGTCGATCGCC	JS9_1_Crispr_2_72	-
CCGGAAGTCAAGGAATGTACCGCCGTGCTCATA	JS9_1_Crispr_2_73	-
GGCACCGCGATGACGATGCCCTCGATAATCGAC	JS9_1_Crispr_2_74	-
TGAATCGCCCTAACGTGAATCTTCTCAAAGTCG	JS9_1_Crispr_2_75	-
TGAGTGAGTCAATCGCGTCGTGTGCGGCCCGCA	JS9_1_Crispr_2_76	-
ATAACCGTAAACATAAAGGGGCATAGAGTAACG	JS9_1_Crispr_2_77	-
CCAAGGTCCTGGGCGGAAATGATGTCCTTGACA	JS9_1_Crispr_2_78	-
AGAGCGGGCACATAGATCGGGGTGCCGTTGTCG	JS9_1_Crispr_2_79	-
TACGCCCACCGCAGCGGCACCCGCGCGGCGTAC	JS9_1_Crispr_2_80	-
TAGCGGTGCTCGATGACCTTGCAGCCCTGAGAG	JS9_1_Crispr_2_81	Anatole, B22,

		Doucette, E1, G4
ACTGCCCGGCGACACAATGGGGCTGGACGCTCC	JS9_1_Crispr_2_82	-
TTCGTCTTGGCCGACGTGGCTCGGATCCTCGAC	JS9_1_Crispr_2_83	-
GTGTACTTGGCGGAGCCGACGTCGGCGCCGATG	JS9_1_Crispr_2_84	Doucette, G4
TAGTGTCAAGACACCTTGAAGGCGAGGCCCTTCA	JS9_1_Crispr_2_85	Doucette
CGTCGTAGATCGTCGCGGTCGGGATCTTGGCGAC	JS9_1_Crispr_2_86	-
GCGGGCGACGCCCCGGACGCCGCCTGGATCGAA	JS9_1_Crispr_2_87	B22, Doucette, E6, G4
CTCGTGAAGTCGGGGCGGTTGACCCAGCTGTCC	JS9_1_Crispr_2_88	-
CACCGCCCACGCCGGGGGAGGGCGGACGCCCCG	JS9_1_Crispr_2_89	-
GAGCATCGGCTGGCCGTTGCTGAAGACCTTCGG	JS9_1_Crispr_2_90	-
AGCTCCGGCAGCGCCTCCCATGCGCCCGGCGGG	JS9_1_Crispr_2_91	-
TTCATATCGCTCAGAGCGAACAGAACTCCTGGC	JS9_1_Crispr_2_92	-
ATCACCAAACGCATCGCCCATCATCCGCGCTCG	JS9_1_Crispr_2_93	-
TCGCCTCTTTCGGGTCAGCGATCGCCGCTCCGTTT	JS9_1_Crispr_2_94	-
CAGATCGCCGTTCTTCTCGGCGGCCGCGATGAG	JS9_1_Crispr_2_95	B22, Doucette, E6, G4
TATCCGTCCCATGTAATGAGAATAAGGTCCCCG	JS9_1_Crispr_2_96	-
TTAACGATAGACGGTGGAATAAGTGGCATATCC	JS9_1_Crispr_3_1	-
TCTTTATCTTCCTGTCTACGTGTGCGACCTTCA	JS9_1_Crispr_3_2	-
GGATGAAGATTGCGATTAGCGGGTCCTGACATG	JS9_1_Crispr_3_3	-
GCGGGTGTGGCTTCGTGACACACATTGCTGGAC	JS9_1_Crispr_3_4	-
TTTGACTGGTCGCCATAATCGTAATTGCCGGTC	JS9_1_Crispr_3_5	-
TGACTAGTAACGTGCCAGGTGCTCGGCGTGTCG	JS9_1_Crispr_3_6	-
GTCCACTTCTCCATCGACACCGACGAGGACACC	JS9_1_Crispr_3_7	-
CTTCCGGAGGGCGCGGAATCCGGGGGTAGGGCC	JS9_1_Crispr_3_8	-
AATCAGGCTGGATTGTCAACACCTTCACCAATA	JS9_1_Crispr_3_9	-
TTGACGGCCGAGACGGTTTGAGCGATGGCCTT	JS9_1_Crispr_4_1	-
CCCCACTTGGATCCGTCCCACGTCTGGGCAT	JS9_1_Crispr_4_2	-
ATCTCGTCCAGGGCACTGCCGGCTGAGTCAGT	JS9_1_Crispr_4_3	-
ATGGAATCCTCAGTGGACGCATCCCGCGCCGT	JS9_1_Crispr_4_4	-
TTCCGCAAGAAGCACCCTGTTGCCAAGACCGA	JS9_1_Crispr_4_5	PFR1, PFR2
ATCGCTGACCAGACCGCCGACGCCACGGGAAA	JS9_1_Crispr_4_6	-
GCCCTGCCGGCCTTTGGTGAGGCGGTCGACGT	JS9_1_Crispr_4_7	-
ACCAGCGTGTGGCGAAATGTCGTTACCTGCTG	JS9_1_Crispr_4_8	B3
CGACGACCTGTACCGGCATTCGAGGTGGCTGGC	JS9_1_Crispr_4_9	-
TTCAACACACCATCAGTGATTAGAAACGCTTG	JS9_1_Crispr_4_10	-
GGAACCTGTACTAAACCTATCTCCCGAATACT	JS9_1_Crispr_4_11	-
CCGGTCAGCGGGATGCCGACTGACTTCAGGTC	JS9_1_Crispr_4_12	-
GAGTTCATGGCCCACGACGGCGCCGATCCTGC	JS9_1_Crispr_4_13	-

CGGTGCCGCTTTCGCGGTGCACCTGTCTTTCGT	JS9_1_Crispr_4_14	Doucette
CCTGCGATATTTCGGCCGGCGATTATGACGAA	JS9_1_Crispr_4_15	PFR1, PFR2
AAAGGTTCAAGCTGCGACATGCCGCGCATGAT	JS9_1_Crispr_4_16	PFR1, PFR2
TGAGGAGGGCGATGATCTCGGTGGTGTGCATT	JS9_1_Crispr_4_17	-
GTTGGATGTTTTCGGTCGGCCCTCGCCTGGTG	JS9_1_Crispr_4_18	-
CCGTCAGGGCGAACTGCCTTGAAACAGGTGCG	JS9_1_Crispr_4_19	-
GGGATGCCGCTTGGGATGCCGCTTGGGGTGCC	JS9_1_Crispr_4_20	-
GTGGTGGCCCGTCCGTCGATCACCGTCGGGGC	JS9_1_Crispr_4_21	-
GATTTTGAGAGTCTCGCGCAGACGCTGCCCGG	JS9_1_Crispr_4_22	PFR1, PFR2
GCCACCCCTACACAGAATGCTCGGGCCTATGC	JS9_1_Crispr_4_23	-
GGCACCGGCCCCCACGCGTTCGCTGGTCGGGT	JS9_1_Crispr_4_24	-
GTCGTCGTGGCGCGCTGGATTTCGATGGGCTG	JS9_1_Crispr_4_25	-
GCATCAAGAATATCCGGAAACCGCCCTGTTCA	JS9_1_Crispr_4_26	-
TATGACCAGCTCACCGCGGACGCTGGGGAGAA	JS9_1_Crispr_4_27	PFR1, PFR2
CCACGGAGCCAGCAAAACTTCGGCGGCGTCCG	JS9_1_Crispr_4_28	-
TGGAGCAGGTCAAGATGTTCAATTACTTACGG	JS9_1_Crispr_4_29	-
CCATGGAGTCAGCAAAATTTCAGCGACACCGG	JS9_1_Crispr_4_30	-
GGCAGCATCGCCAGGGCTTCGTCGCCGAGCAT	JS9_1_Crispr_4_31	-
TCCGCCAACTTCGTCCACGCCTTCACCTGGTT	JS9_1_Crispr_4_32	PFR1, PFR2
TTCGCGCCGATACGCCCCACCGAGGCAACCAG	JS9_1_Crispr_4_33	-
CTCGCCATGTCGGCGCCCAACCCCGGGGTGGT	JS9_1_Crispr_4_34	-
GTCGCGGCATGAACCGCACCTATTTCAGGGCT	JS9_1_Crispr_4_35	-
TTGATGGTGTTGTGCATCAGTTCTCCTTGAAC	JS9_1_Crispr_4_36	-
CACGAGACAGAACAAGATCATAGCCCCGCTTT	JS9_1_Crispr_4_37	-
TTATCTATGATGAACTCGTCATAGATGATTAGC	JS9_1_Crispr_4_38	-
TTAATGATCCGCATTACTATTCTGATGGGCGC	JS9_1_Crispr_4_39	-
AATTCTGTCCGGAGCTTCTGCATCCGCCTCGA	JS9_1_Crispr_4_40	B3
ACAGCGGAGGTCTGCGCACCAAGCCGGAGACG	JS9_1_Crispr_4_41	-
GGTTGAGCCGGCTGGCCGCGTGGCTCTCCAGG	JS9_1_Crispr_4_42	-
ATCGACGAGGAGGACTCATGAGCACCCCCACA	JS9_1_Crispr_4_43	-
GATCGAGGCCCTCTTGTGGGGTAGCGCCTCAG	JS9_1_Crispr_4_44	-
GTTGTGGTTGCTATCCGCACAAAGACGGGCTT	JS9_1_Crispr_4_45	-
CTGACGGTTCCGTGTGCGGGTATGTCGAGTTT	JS9_1_Crispr_4_46	PFR1, PFR2
ACTTCCGCACCGAGGTGCTGTGTCAATGGGTG	JS9_1_Crispr_4_47	-
GGCCTGATGGCCGAGCCTCCGACCACGGTGGT	JS9_1_Crispr_4_48	PFR1, PFR2
TGACGATGCCCCTAAGGAATGTTATTGACCAC	JS9_1_Crispr_4_49	-
CATTCAGGTGCGTCAGGCAATTGGGTCGACAC	JS9_1_Crispr_4_50	-
AAATGACAAATTGTTGTCCTGAATACATTCAG	JS9_1_Crispr_4_51	-
TGATAAACGTTGCGGTTGCAGAATCACCCTTA	JS9_1_Crispr_4_52	-
TGTGAGGCCGCCCCAGATGCCCGTTTCTGGGC	JS9_1_Crispr_4_53	G4
GGTTTGGGTGAGCAGCTTCGCCCAGTCCTTGT	JS9_1_Crispr_4_54	PFR1, PFR2
AAGCTGGGCGGGGGTGTCAGTCGGTTCCATTG	JS9_1_Crispr_4_55	-
CCCGTCCGCCCCAACACATGCGTCGTGGAATT	JS9_1_Crispr_4_56	-

CAACGGGCGTGGCCGGCACCTTCACATCAGGG	JS9_1_Crispr_4_57	-
GAGACAGAAAAAGCCCCGGCGAGAAGCCACTC	JS9_1_Crispr_4_58	Doucette, B22
GCGCAAAGTTACGCGGAGTCGTATCGGATGGC	JS9_1_Crispr_4_59	-
TCGCCACGGCGGGCGCGCTCGACGCCTTTCGA	JS9_1_Crispr_4_60	-
TTGAAAGCCAAGGCATTACCCTGCAGGGACTT	JS9_1_Crispr_4_61	-
GTTGCATTATCGTTGAATGTAATCGGCGTCGT	JS9_1_Crispr_4_62	-
GCCGGCATTCAGTGCATCATGGTCGTTCCAAA	JS9_1_Crispr_4_63	-
TCGGGCGAAGACCCTGCCAGGTATAACGATGC	JS9_1_Crispr_5_1	-
TTAGGAAGAGTATTTGTCCTTACTTTGAGTGG	JS9_1_Crispr_5_2	-
GGACGCGTTCTTGGCGACTTGGCCGGGGGCGG	JS9_1_Crispr_5_3	-
CGATGACTGACCTGCTGCCCGACATCGCAGCGA	JS9_1_Crispr_5_4	PFR1, PFR2
CGCCCCCGTAGTTGGTGCGTAGGTCGGCATCGA	JS9_1_Crispr_5_5	-
CAACTGTGGGAAGTCGACGGCGACCCGGTGCG	JS9_1_Crispr_5_6	PFR1, PFR2
CCTGGATCGCGTTCGGGTCGCCGCCCGACTCG	JS9_1_Crispr_5_7	-
CGCTTGCGTGCCATGGATTGGCGGACCCGCAAG	JS9_1_Crispr_5_8	PFR1, PFR2
ATCGCGGCGGCGACCCGCTCCCCTGTGTCGGG	JS9_1_Crispr_5_9	-
CCCGAGCCATCAAGGCCGCCCTGGACAGTGGA	JS9_1_Crispr_5_10	-
TCCCGTCGCCTCGTCGATGGTGAACATCGACA	JS9_1_Crispr_5_11	-
TCAGCCAGGTGGTGGGGTGTTTGATAAAGCGT	JS9_1_Crispr_5_12	PFR1, PFR2
GCCCCCGAAATTGCTCCGCAGGTCGGCGTCGA	JS9_1_Crispr_5_13	-
CTAACGAGGAGAGAACCTTTGCGGGAGAGTTG	JS9_1_Crispr_5_14	-
TCGCTCGAACAGACCGTCGGCACACTGTCGGC	JS9_1_Crispr_5_15	-
ATGGTGCGCCGATCCCTGCCGAAGGTTGGGTC	JS9_1_Crispr_5_16	-
ATGGCGCAGCTGCTACCCGACATCGCCGAAAA	JS9_1_Crispr_5_17	-
TTAAAGCCGTGGTGGACTGGTTCAAGAACTCG	JS9_1_Crispr_5_18	-
CGCACCACCCGCAAGGCGACG	JS9_1_PossibleCrispr_1_1	-
AAGCCGGCGGCCAAGAAGCCGGCA	JS9_1_PossibleCrispr_1_2	-
ACGGCGTCCACGGCCAAGCCGGCA	JS9_1_PossibleCrispr_1_3	-
TGCCCACCTGGCCAGAAACCTCTACCCCGAGCCCCTTGA	JS9_1_PossibleCrispr_6_1	-
ATCAGCCGGATAACCATTCACCGGCCCCGAA	JS9_1_PossibleCrispr_7_1	-
CTGGGTAGTAGTATAATCTTACTTGTTGATGAAGGGG	JS10_Crispr_2_1	-
CCGACGCTTGCGTGGAGCAGCTCGGCGACGTCCTTCTG	JS10_Crispr_2_2	-
TGTTGGATGCCTGGCTGGTGGGGATCGTGCGCCACAGG	JS10_Crispr_2_3	-
CCCCGAACTGGCGGTCCCGGCCGCCTCTTCTTCCGT	JS10_Crispr_2_4	-
CCTGTCATTCGCCCATTGGGCCGCTTGAGAAGCTTGAT	JS10_Crispr_2_5	Anatole, B3, E1
GCTCGGGGATGCTGCCCTGGCGATGCTGCCGACCGC	JS10_Crispr_2_6	-
GACAGCGCGGTCCGCTCGATCATCGGCGACCAGCCCT	JS10_Crispr_2_7	PFR1, PFR2, G4
GACGATACAAGACTGACCTAACGAATAAGGAAACG	JS10_Crispr_2_8	-
GCCTCCAGCGGTGGAAAGAGCGGCAGCGCTACGGACG	JS10_Crispr_2_9	-
CCGACCGCCAAGATGATCAATGAGCGGAAGCACAC	JS10_Crispr_2_10	PFR1, PFR2

TTGAAGCAGGCGCCCGCAGCGCCCCATCGCCCGG	JS10_Crispr_2_11	-
GACCGGGTGAAGCGCGCCGGGGGCCTGAGCATCAAAC	JS10_Crispr_2_12	-
GGATCGTAGCGGGGCTGTTGGCAGCAAGTCTCGCTG	JS10_Crispr_2_13	-
GACTACAGGTGTTCGATCTGGTCTGTGGCCGTCGA	JS10_Crispr_2_14	-
GTAGGTGGAGGGGCGCAGACATTAAAATCTACGCCC	JS10_Crispr_2_15	-
CAGAACAACGCTCGAGGAAAGGGTTCCAATCTCATG	JS10_Crispr_2_16	-
GGTGTTGAGTTACCGCCATATTCGGCCAGAGTCTTGAA	JS10_Crispr_2_17	-
CCGTTTCGGCTGTCGCCACCAACTGCTCCGGGATGGGC	JS10_Crispr_2_18	-
ACACCCGCCGCGATCGAGGCCGACCTGAAACGCTG	JS10_Crispr_2_19	-
GACGGCGGGCCAGTTGCACAACACGGCCACCCGCACCAA	JS10_Crispr_2_20	-
TTCGCGGTGCGGTCGGCAATGCTGGACGCGTGCCCGA	JS10_Crispr_2_21	-
CTCACGCCCGGAACCCATGCCGGAAGATCAGTCGCC	JS10_Crispr_2_22	-
GCGGCGCCTCAGATGGACCTGTCCAGCGGTGAGC	JS10_Crispr_2_23	-
ACGGCCTCGCCGTCGCGGCTGATGCTCGCATGCATC	JS10_Crispr_2_24	PFR1, PFR2
GCTCGGCGACGCCGCCCTGGCGATGCTGCCGACCG	JS10_Crispr_2_25	-
GTGGTTTCGACGCCCTTGTCGGGGCCGTGCCTGCC	JS10_Crispr_2_26	-
GCCGGTGCTCCCGTCACGCCGGGGCGTTCTTCGG	JS10_Crispr_2_27	-
ACCTGGGCGGCCTTCGGATTGTCCAGGTCAAAGAAG	JS10_Crispr_2_28	-
GGCGAGTTGGCTCGGACCTCTGGACAATTTTTGGA	JS10_Crispr_2_29	-
GCAGCTGCTGGTCGCTTTGGTGCAGGGCATCCTCTC	JS10_Crispr_2_30	-
TATTCACAGGATGCATGGGTTCGCTCACATATGGACG	JS10_Crispr_2_31	-
CCGAGATCACAGATGCCGAGCTCATCACCATTCA	JS10_Crispr_2_32	-
AGACGTGTCAGGCGACAGCGGAACCAAGACAGCCG	JS10_Crispr_2_33	-
TTCGCCGTGAAGACCCGCGAGGCGGAGGTCGCCCGT	JS10_Crispr_2_34	-
GGCGGGTCCGCGAGATTCGTCGGCGTCGCACGGCGG	JS10_Crispr_2_35	Anatole, B22, B3, E1, E6
GTGAGCTCGGGCTGAGCGTGAGCCAGACAGCGGCAAAG	JS10_Crispr_2_36	-
GCGAAAGAGCGATCATTCCGGGTATGGATCAAACC	JS10_Crispr_2_37	-
GGGGTCTGCAACGGGGTCAGCCCAACAAGCTGACCCACTGGATCCACCACA	JS10_PossibleCrispr_1_1	-
CTGGAGCTTGCCCTTGGCGGCCACTGATCCGAAGA	JS10_PossibleCrispr_3_1	-
CTGGCCAGAAACCTCTACCCCGAGCCCCTTGA	JS11_PossibleCrispr_1_1	-
TCAAGGAGCGGCGTGGAGCCCGCCTTCGCTCCAG	JS11_PossibleCrispr_2_1	-
CCTCGTGAAAATCGCAGCCCTGCTCCACATGCCCCCC	JS12_1_Crispr_1_1	-
GGCGAGGTCGGGCTGCAGTACGGGAACCAGCGCAAGC	JS12_1_Crispr_1_2	B22, Doucette, E6, G4
AGGTCACAGGCGAACCTGCGCACGGTGATTGACTTCCAG	JS12_1_Crispr_1_3	-
ATCTCGACGTGGTCGAAAGTCGGATTGTGGCCTGACC	JS12_1_Crispr_1_4	-
TGTTTCCAGCGGGATTGCAGTGTGCAATTTCATGACCC	JS12_1_Crispr_1_5	-
GAAGCTGGGCGGCACGCTGTCTGGCTGGGGCAGCTCGA	JS12_1_Crispr_1_6	B3
AGCCGGTCGACATTGCAACCAACGTTCTACGAGGCG	JS12_1_Crispr_1_7	-
ACACGATCCCCGGGGCGATGACTCCCATGCCCGCTACG	JS12_1_Crispr_1_8	-

GGCGGCCCGTTCAACATTCCGGGGATTGCCTCGCTG	JS12_1_Crispr_1_9	E6
GCGGGTCGCGACAACGTGGCCGACGATGACCCCAG	JS12_1_Crispr_1_10	-
TGTTCTTACAGAAGTTGGATACACGCTAAAGTTCTAGG	JS12_1_Crispr_1_11	-
TCGAAGTTGGACGAGGACAATCATCGGGCAATTCTGGGAA	JS12_1_Crispr_1_12	-
TTGCACACCTAACATGGTTGTGATGGCGGACAATCAGCCT	JS12_1_Crispr_1_13	-
TCACCCCCGGTTCGCCTTAAGCAGCCGCCACCGCT	JS12_1_Crispr_1_14	phiB5
ACCATGACTTTCTCGGATGCCTGAAGTGGCGCACCTTCGG	JS12_1_Crispr_1_15	-
TGCCGCTGGTCCACCATCACGTCGTGGCCGAGCAT	JS12_1_Crispr_1_16	-
TGGTAATACATTAGGACTGTGGCCTGTAGTTTTACAG	JS12_1_Crispr_1_17	-
AGCACAAGTGCCCGGCCTGCCATTACGACACGCCGG	JS12_1_Crispr_1_18	-
GATCCGATTCCCGCAGCGCCGCTGATCGGTGTCGCCA	JS12_1_Crispr_1_19	-
TTCATTGACAGAGTTCTCCATTGCATCAACATCCTT	JS12_1_Crispr_1_20	-
AGTCAGGATGCATGGGTGCGTAGCCACATGGATGAAA	JS12_1_Crispr_1_21	-
AATTTGTGGGGGTTCCCGGTGATTGTGACTACCGC	JS12_1_Crispr_1_22	-
GAACGGATCGATCGACTTCAACCCGAAATTCTTCAA	JS12_1_Crispr_1_23	-
CACAACCCGCCCTACTGGCGCACCCGGCAGTCAGCT	JS12_1_Crispr_1_24	-
CCTACCAGCCCAATGTATACGGACCGATCGGATGGTCATC	JS12_1_Crispr_1_25	-
TCGTCGGGCGGCATGGTGCGCGGCGACCGCACCCAAA	JS12_1_Crispr_1_26	PFR1, PFR2
ATTAGTAAGGCGCCTACGACCGTGGATGTTTCCGT	JS12_1_Crispr_1_27	-
CCCGAGGCGAGTTCCCTCACGTTACTGAGCGTGG	JS12_1_Crispr_1_28	-
TCATGAGAGTTGAGCTCTAGCGGTACCCGTAACTTCAGAAG	JS12_1_Crispr_1_29	-
CCGGCAGCGGTGGTGATGTCGTCCACATTGGCGGT	JS12_1_Crispr_1_30	-
GTCGAAGGAATCGAGCAGCGCTCGGCATCCGACG	JS12_1_Crispr_1_31	B22, Doucette, E6, G4
AAAAAAAGCTGCATCCCAGCTGCCAACCTCCCCTGGG	JS12_1_Crispr_1_32	-
ATTCCACATACCCGTCAGGCAGCTTGGAGATGCG	JS12_1_Crispr_1_33	-
ACCCGCGTCACCCTGCTCGAAGACCCCGACGAACCGGACC	JS12_1_Crispr_1_34	-
CTGTCGACCTCGACGGTGGAAACCGACTGCACAGG	JS12_1_Crispr_1_35	-
CAGGGCGTGGTGAAGCTGTTCTGGCAGAATCCTGCC	JS12_1_Crispr_1_36	-
TTCGGGAAGCTCACAGAATCATGGCGCACGGTGGA	JS12_1_Crispr_1_37	-
GTCGAATTCAACATTTCTGCCAAGCTCCAGGGTG	JS12_1_Crispr_1_38	-
TTACCGGTCTTACCCTCTAGCGCCGTGGACGACCATACC	JS12_1_Crispr_1_39	-
ACCCACTCGTTGTAAAATCCACCGACCTCGGCGGAG	JS12_1_Crispr_1_40	-
GGCGCCGCTCGGGACGCTGCTCGGG	JS12_1_Crispr_1_41	-
ACTCCTTGTTGTTGGCAGTTCGTTTGGTTGGCCCTCGC	JS12_1_Crispr_1_42	-
AAGTGGCCGGCGGGCAGGGTCCAGGCGGGCAGGTCCA	JS12_1_Crispr_1_43	-
GGCGGCCGTTCCGATTGGTTTCCGCCGTTCGATAAG	JS12_1_Crispr_1_44	-
CCAAAAGGGCAACATACAGTACATCCGCAACGAGGC	JS12_1_Crispr_1_45	-
TCCGTCAGGTGCTTGAGGGTATGGGGCTGACGTTCT	JS12_1_Crispr_1_46	-
GTGCGCATCAGATCACACGCCAGCAGTGCCGAGGGC	JS12_1_Crispr_1_47	-
TTCGCGTCGGCGACCAGCGATCTGGCTGGTCCGG	JS12_1_Crispr_1_48	-
GGATTCACCGAGGTGGCGTTGCAGTCGCGGGGTGCCCCGG	JS12_1_Crispr_1_49	-

CACGATGCGCCGTGGGCGACACGACGCCTTTGGGG	JS12_1_Crispr_1_50	-
CAGGGCGTGGTGAAGCTGTTCTGGCAGAATCCTGCC	JS12_1_Crispr_1_51	-
CGCACCAAGGCCCCGGCTCGGCGAGGGACGGTTGCCGT	JS12_1_Crispr_1_52	-
GTCATGGTGTGCGCACAGAAACCTAGAGTTCGGGCGG	JS12_1_Crispr_1_53	-
GCGAAAGAGCGATCATTCCGGGTATGGATCAAACC	JS12_1_Crispr_1_54	-
CTGGCCAGAAACCTCTACCCCGAGCCCCTTGA	JS12_1_PossibleCrispr_2_1	-
TCAAGGAGCGGCGTGGAGCCCGCCTTCGCTCCAG	JS12_1_PossibleCrispr_3_1	-
CTGGCCAGAAACCTCTACCCCGAGCCCCTTGA	JS13_1_PossibleCrispr_1_1	-
TCAAGGAGCGGCGTGGAGCCCGCCTTCGCTCCAG	JS13_1_PossibleCrispr_2_1	-
GTTGAAGCAGGCCGACACTGCTGGGCAGGTCCGTG	JS14_PossibleCrispr_1_1	E1, Anatole
CTGGCCAGAAACCTCTACCCCGAGCCCCTTGA	JS14_PossibleCrispr_2_1	-
TCAAGGAGCGGCGTGGAGCCCGCCTTCGCTCCAG	JS14_PossibleCrispr_3_1	-
ACGGCAACCGTCCCTCGCCGAGCCGGGGCCTTGGTGCG	JS15_1_Crispr_1_1	-
ACGCAGATTGGAGGAAGCAAATGGGCAAGCAAATGC	JS15_1_Crispr_1_2	-
CTTTGCCGCTGTCTGGCTCACGCTCAGCCCGAGCTCAC	JS15_1_Crispr_1_3	-
GGTCCGGCTCCTGCGCCGCTGGGAAGCCCTCGCCGCCCAC	JS15_1_Crispr_1_4	-
TCGACAACCGTGGTGTTCATGGGCATGCTCCTTTGA	JS15_1_Crispr_1_5	-
TCAAGACCGAACCGGATCACATGCGCACTGTCGCC	JS15_1_Crispr_1_6	-
CTGTCGAGCAAATTCAGCTCACACAGGAGGAAGCCCA	JS15_1_Crispr_1_7	-
CCTACTATTGATGCCACAACGGCATTTCAGGAGAA	JS15_1_Crispr_1_8	-
CGCGTGCGCGGCCCGCTTGCGAGGAAATTTGTCGGG	JS15_1_Crispr_1_9	Anatole, B22, B3, E1, E6, G4
CGGCCCCGTCTGGTCGGACAGCCTTGAAGAGCCGG	JS15_1_Crispr_1_10	-
TTAGCGAGTTCTGCCCAGTCGTCTTCCCACACCTGCCC	JS15_1_Crispr_1_11	-
GGGATGGTTGGCGATGGTCCGCCCGATCACATCGAC	JS15_1_Crispr_1_12	-
TGTCAGCTCAAAATCAGCCAGTCGGCTACAATACAT	JS15_1_Crispr_1_13	-
TCAAAGAAATGGGGCTTGTTGCGCAGCTCGGTTCG	JS15_1_Crispr_1_14	-
CGGTGCAGGGTGCCCGATCCCGCTGTTACCGGACCC	JS15_1_Crispr_1_15	-
CGCTGAAGTGATGGCATTCATCAGAGGAGCGAGCTTCGG	JS15_1_Crispr_1_16	-
AATGTTGTGGTCATACCCGCGCCGGGTGCGGAACGT	JS15_1_Crispr_1_17	-
TGCTAGAGAGGTTCTGGGGAACTATGGATCGTCGT	JS15_1_Crispr_1_18	-
CTTGGGGGCCGGTCCCTGGAGTGGGTCAGTCTTCGT	JS15_1_Crispr_1_19	-
CTTATCGAACGGCGGAAACCAATCGGAACGGCCACC	JS15_1_Crispr_1_20	-
ACCGTTGGTCCTCAAGCCCGAATGTATTCAGGATG	JS15_1_Crispr_1_21	-
CTGTGAGGCTCGTGCCGTCTGTTGTGTAGGGGTGGG	JS15_1_Crispr_1_22	-
CGGGCCGCCGTCGTCCTTGAACAGCCCGACCTTCGC	JS15_1_Crispr_1_23	-
CTGAAGGTGTTGACGTTCCTACGAAGAAGGCTGTATA	JS15_1_Crispr_1_24	-
CGTCTCGTGGCATGTACATTCTGCCACCTGCTCAAC	JS15_1_Crispr_1_25	-
GTGGGTGTTCTGGTGGTCGTGGGTGTCGGGTTCGGGGC	JS15_1_Crispr_1_26	-
CTCAGCGGCAGATGCTGGTCAGTGAGGTCCTCACGAG	JS15_1_Crispr_1_27	phiB5
GATCTTGTGGTCCAGGACCGTGCCTGTGGTCCTGTC	JS15_1_Crispr_1_28	-
ACACCGATCAGCGGACTGTCGGTGGGAATCGGATC	JS15_1_Crispr_1_29	-

CTGTGGCGCACGATCCCCACCAGCCAGGCATCCAACA	JS15_1_Crispr_1_30	-
CATTAATAGGCTATATCTTTTTAATTCCTAAAAATAAAG	JS15_1_Crispr_1_31	-
AATATGCGGCGCACCATGCCTCCATGTCGGGAGGC	JS15_1_Crispr_1_32	-
AAGCGGAGCGACAAGAGCGAACAGGGAGATTTTTTG	JS15_1_Crispr_1_33	-
GCCCCGGGACGACAAGCGCCCTGCTGTGTTCGTTTGCTCTACGGCGA	JS15_1_PossibleCrispr_2_1	-
TGCCGGCTTGGCCGTGGACGCCGT	JS15_1_PossibleCrispr_3_1	-
TGCCGGCTTCTTGGCCGCCGGCTT	JS15_1_PossibleCrispr_3_2	-
CGTCGCCTTGCGGGTGGTGCG	JS15_1_PossibleCrispr_3_3	-
GGTTTGATCCATACCCGGAATGATCGCTCTTTCGC	JS16_Crispr_1_1	-
CCGCCCGAACTCTAGGTTTCTGTGCGCACACCATGAC	JS16_Crispr_1_2	-
ACGGCAACCGTCCCTCGCCGAGCCGGGGCCTTGGTGCG	JS16_Crispr_1_3	-
TGGGCAAGACGACTCTGTCCACGATTGAGGGTGACG	JS16_Crispr_1_4	-
TTGCGATCATCTGGTCCACGTCGAGCCCGGTGCC	JS16_Crispr_1_5	-
AGAACGTCAGCCCCATACCCTCAAGCACCTGACGGA	JS16_Crispr_1_6	-
ATCGTCTGCTTGGCAGTCTCCGGGATACCCCCAAAGAT	JS16_Crispr_1_7	-
CCTACGGTTTGACGATGAGCAAGGGTGAGATCGA	JS16_Crispr_1_8	-
GTGGGGCCCTTCTTGATTTGTCCTTGTGGTACACAGT	JS16_Crispr_1_9	-
CTTATCGAACGGCGGAAACCAATCGGAACGGCCGCC	JS16_Crispr_1_10	-
TGGACCTGCCCGCCTGGACCCTGCCCGCCGGCCACTT	JS16_Crispr_1_11	-
GCGAGGGCCAACCAAACGAACTGCCAACAACAAGGAGT	JS16_Crispr_1_12	-
TTGAGTCGCGAAAAGCCTTTAACGAGAGCCACCCCTGCG	JS16_Crispr_1_13	-
CCCGAGCAGCGTCCCGAGCGGCGTTCAGAGCGGCGCC	JS16_Crispr_1_14	-
CTCCGCCGAGGTCGGTGGATTTTACAACGAGTGGGT	JS16_Crispr_1_15	-
GGTATGGTCGTCCACGGCGCTAGAGGGTAAGACCGGTAA	JS16_Crispr_1_16	-
CCGGACCAGCCAGATCGCTGGTCGCCGACGCGAA	JS16_Crispr_1_17	-
GCCCTCGGCACTGCTGGCGTGTGATCTGATGCGCAC	JS16_Crispr_1_18	-
CACCTTGTAATCAAGTCTGTCAAGTGAGCTGGTAGG	JS16_Crispr_1_19	-
ATCACCCCTTCAGCAGGAAGCCAGTGGACCTTACCG	JS16_Crispr_1_20	-
CCTGTGCAGTCGGTTTCCACCGTCGATGTCGACAG	JS16_Crispr_1_21	-
GGTCCGGTTCGTCGGGGTCTTCGAGCAGGGTGACGCGGGT	JS16_Crispr_1_22	-
GCCACGAAGGGTGAGTCGGTGACGGCGCGAGAATAG	JS16_Crispr_1_23	-
ATTCGCCAGGTGAGCGGGAAGGGTGCAAATAGCTA	JS16_Crispr_1_24	-
TGTGCGACGCCCAGCCGGCGCCAACGATATTGTGGTC	JS16_Crispr_1_25	-
TTCAACGACCGGCCCGACGCCTTGAGCTCCTTGACGAA	JS16_Crispr_1_26	-
GGTGAGCTCTGACTGGATTGTGTTCATGTGGAGACC	JS16_Crispr_1_27	-
AGGAGGCGTCCGCCATCCCTTTTCCAGCCAGGCATCGG	JS16_Crispr_1_28	-
CTGGAGGCGCTGCGAGGTGCCGCCAGACAAGA	JS16_Crispr_1_29	-
TGCTTGCTCAGAGCTTGTCTCTTTTGGTCGTTTTGC	JS16_Crispr_1_30	Anatole, B22, B3, E1, E6
TGCTTGCTCAGAGCTTGTCTCTTTTGGTCGTTTTGC	JS16_Crispr_1_31	Anatole, B22, B3, E1, E6

TTGCTGTGCAATGGCAGTCACTCCCGGTGGTCTTGCCT	JS16_Crispr_1_32	-
TCTCCGCCACCAAGGCCAAGGAGACCCTGCCGCCCGACGT	JS16_Crispr_1_33	-
TTGTTTCTCCCTAAAACTTTAGTGTATATCCGACTTCT	JS16_Crispr_1_34	-
CAGAAGTCTGTGCCGTCCGGGCGAACAGACTTAAA	JS16_Crispr_1_35	-
AATAGGTGCGGTTCATGCCGCGACCGCGACGCTCGG	JS16_Crispr_1_36	-
CAGTCGATCCGTCTGTCGAAGGACTCGAACGGCCA	JS16_Crispr_1_37	-
GGGTTTACGCGAGGGTGAGCATCAATGCCTACCC	JS16_Crispr_1_38	-
GGGATGGTTGGAGATGGTCCGCCCGATCACATCGAC	JS16_Crispr_1_39	-
GATTACGGCTTTTCCGTTCAGGAATTAGCGGACGGT	JS16_Crispr_1_40	-
CTCCTGCTCGTGGAGCCTGTAGGTGCCATGTGGACCCC	JS16_Crispr_1_41	-
TGGTTTCCTGTGAGCGTGTTGTAGGCGCTCTGATAGCC	JS16_Crispr_2_1	-
ACCGAACATGCCCTCAGCGCCATAACGCGACTTCGC	JS16_Crispr_2_2	-
TCTGGCTGTGGCACCTATGCGAAGGATGCCTTGG	JS16_Crispr_2_3	-
ATTTTGCTCACCCCCTTTCATCAAAAAACTGAAT	JS16_Crispr_2_4	-
AATCCTTTCTCCCCTTTCACGCAGTTCGATGAATG	JS16_Crispr_2_5	-
TGGTGGAGCTTCCCCCTTGACATCGCGAATACTATGGCC	JS16_Crispr_2_6	B22
TCGCAAGCTATCCAGGCGAACGCCACCATGACGTCCCG	JS16_Crispr_2_7	G4
ATGAGGACCTTCTCGAAGCTCGGGTTGATCAGCAA	JS16_Crispr_2_8	-
TTGACCTCGTCGGCACCGAACTTCGTCGCCACCCAGTT	JS16_Crispr_2_9	E6, B22
GATGGAACGAACAATGCTCGAGCGAGACCTGGAGTT	JS16_Crispr_2_10	-
GAACCGTGTGGCGACACATCGAGGAGCACGGTGACGG	JS16_Crispr_2_11	-
CGGCCGCGAGGGCTTCGAGTATCAGGCGAGCTTCGGTGA	JS16_Crispr_2_12	-
TCTAGCCAGACGATCACGATCTGTGGCGGCATCGTC	JS16_Crispr_2_13	-
CCCATCACCTCCGACACGTAGGCCGTCCATGACTT	JS16_Crispr_2_14	-
GTCTCAGGGGCGCAGATGATGACTCGTCGTCTTCATT	JS16_Crispr_2_15	-
TGTGGGCCAGCGTGGTGGTCAGCGACTTGACCGACGC	JS16_Crispr_2_16	PFR1, PFR2
AATCTGAAGCCATAGAGAGTCGCATGCCGCCCTCAGA	JS16_Crispr_2_17	-
GATCCACATGGTAGACCGGCGACTCATGATGCCCATCC	JS16_Crispr_2_18	-
CGGGGTGTCGCCTGGGATGCCGCTCGATGTTCCG	JS16_Crispr_2_19	-
CCGCATCTATGACACCCAGTTCGCCACGGAATACAT	JS16_Crispr_2_20	-
CAGTTCGATCATTACATCGAAGGTCTTATCGAAAA	JS16_Crispr_2_21	-
GGTTGCGTTACTCGTGCGTCCTGGCACTTGGCATTG	JS16_Crispr_2_22	-
TTGAGGACCAGAGGCGTTCCCATACTAGTCGTAGCTTCA	JS16_Crispr_2_23	-
GAGGACTTCGGGAGTCAGTTGGGGTGCCGCCGGGGC	JS16_PossibleCrispr_3_1	-
CGGCCATGCACGGGGCGGCCTCGCGCTC	JS16_PossibleCrispr_4_1	-
CGCACCACCCGCAAGGCGACG	JS17_1_PossibleCrispr_1_1	-
AAGCCGGCGGCCAAGAAGCCGGCA	JS17_1_PossibleCrispr_1_2	-
ACGGCGTCCACGGCCAAGCCGGCA	JS17_1_PossibleCrispr_1_3	-
TGGCGTCCGTGTCCACACCATTGCCGCCCTGCACCTG	JS17_1_Crispr_2_1	-
GTGTTGCTCGGATGCATCATCTCGACCACCCGACCGG	JS17_1_Crispr_2_2	Anatole, B3, E1
TTGAAGAGCTTTGCGACGAGCGTCCTGTCCGGGGT	JS17_1_Crispr_2_3	-
GACCGTGACAATGGTGCCAGACCGTTACAACGAATC	JS17_1_Crispr_2_4	-

GCACCACACGTCGGCGGTCGGCAGCATCGCGAGCGC	JS17_1_Crispr_2_5	-
GAGAAGGATGGGGCGCGGTGGCATCTGGTCGGTGCTAAGG	JS17_1_Crispr_2_6	-
ATCGCTTGGGAGGGTCGTCATGGCCATCATTGCGGT	JS17_1_Crispr_2_7	-
GTCGATTCGGACGGTCAAGTAAATGTGGGAACCGATC	JS17_1_Crispr_2_8	-
TGATAGCTGTAATGTTGTATGCCTCTTCGAGCGCCGT	JS17_1_Crispr_2_9	-
GAGGTACTGCGGCCCGCCGACAAGAACTTCCAGGCCGAAC	JS17_1_Crispr_2_10	B22
CAGCAGAATAGAGAACTTGACCTTCGGCGTTCTTCTT	JS17_1_Crispr_2_11	-
CGAAGTCGTTAGAGGGTACGCTAGAGGGCCGGCAG	JS17_1_Crispr_2_12	-
CGACAGCCAGCCTCAGTCCTTCTGGAGGAAGTCCGG	JS17_1_Crispr_2_13	-
AGCGGGTGAATACCTGTGCACCCTGCTGGAATGCGCCCA	JS17_1_Crispr_2_14	-
AGTGGGTGTCACAAGGACAAAGCAAGAGGCCCCACC	JS17_1_Crispr_2_15	PFR1, PFR2
TATCAGTAGTCATATCTGTAAGAACATAGCCTATT	JS17_1_Crispr_2_16	-
AATTTGCGGGCCATGCCCTATGGTGGGAACGATGGGCT	JS17_1_Crispr_2_17	-
CGCAACCAGAACTCGCCCAATGCCTTTCGAGCCGAA	JS17_1_Crispr_2_18	-
CAACCATGGAAGGATCGGGCGGGGTGTACTGGTT	JS17_1_Crispr_2_19	-
GATAAACACAATCATCTCAACGAAATGAAACATACGC	JS17_1_Crispr_2_20	-
GCGTCTCGCGTCTACAAGGCTCGACCATTACACGG	JS17_1_Crispr_2_21	-
GACCTGTCGCTGCGTGAGGAGCTGGCGTTGCTGTC	JS17_1_Crispr_2_22	-
GGGGCAACCACTTGCGTACCATCGTCCCGGGACAAC	JS17_1_Crispr_2_23	-
GAGTCCTGGTGTCGCGATGCAGGAGAGGCGCCCATGTC	JS17_1_Crispr_2_24	-
CAGCGCGATTCGGGCTCATCGAATCACTGGGACCACG	JS17_1_Crispr_2_25	-
TGTTTGGGCTTGGGTTGCGTTGCCTTCTCTGCCGCCT	JS17_1_Crispr_2_26	-
CGAGATGGCCGGATGTGGAATTGGATTACCGTGG	JS17_1_Crispr_2_27	-
GACATCGACGCCATCGCCGACGAGCTGGTCACGCTC	JS17_1_Crispr_2_28	-
CGGCAAGGTCCACTGGCTTCCTGCTGAAGGGGTGAT	JS17_1_Crispr_2_29	-
GGCCAGACGTGTGCACCGACGGCCACCGGGTAGACC	JS17_1_Crispr_2_30	-
GCTCGTAATTTTGAACCATCCCAGGAAAAGCGGACGC	JS17_1_Crispr_2_31	-
CAGAATCGACAATTCGATTAGATACCGGAGCAGCAA	JS17_1_Crispr_2_32	-
AGAGCGGCTGGAAAAGCTTCGGCACAATACCGGGCAG	JS17_1_Crispr_2_33	-
AAGCGGATGCCGTGGCTTCTTTCGGTGCCGATTCC	JS17_1_Crispr_2_34	-
GTGGGCGGTGCCGCATGATGCCGGCCACGCCCGTTGGT	JS17_1_Crispr_2_35	-
TCCTTTCCTGCCGGCCGGGGCACTCAAGACAACCGGGG	JS17_1_Crispr_2_36	Doucette, B22
AGGTCACACGAGCCGGTGATCTTGCCGAGGGTCTT	JS17_1_Crispr_2_37	-
AGCCTTCTTCGTGGGAACGTCAACATCATTGGCGGCG	JS17_1_Crispr_2_38	-
CACCTTGGATCAGCAACATCCATCGACGAGGCAGT	JS17_1_Crispr_2_39	-
TTAATCCCTTCGATAATAGAATTGGGGTTCCAATA	JS17_1_Crispr_2_40	-
TAACCGTTGAAAACCGGGGCGTTCTCGTTGATAA	JS17_1_Crispr_2_41	-
GATAGAACACGAAGGGATTACGAGGACTTTTGTTA	JS17_1_Crispr_2_42	-
AGGTCTGGGTTTTGCGAATCCTCATGATTTCGGCCCC	JS17_1_Crispr_2_43	-
AAGAAGCAATCACCACACGCCTCACCCAACGGGGC	JS17_1_Crispr_2_44	-
CCAGGGGGTTCTTCGCCTCGTCGCCGCCAACGGTGAT	JS17_1_Crispr_2_45	-
CTGAAGGGCCGCCGAGAGATTCCCATGTCGACCTTCCT	JS17_1_Crispr_2_46	-

TTGGGGCGAGGTCGGTGGCCAGATCCTTGCCGGGATCGG	JS17_1_Crispr_2_47	-
GAGAAGGTCGGGCTGTTCAAGGACGACGGCGGCCCG	JS17_1_Crispr_2_48	-
TTCGGCACCGATTTGGCGTTGGTGTCCGACCGGAT	JS17_1_Crispr_2_49	-
TAATAGTATCAGGAATAGTGCGGGACTTGAGTTTAA	JS17_1_Crispr_2_50	-
GCCACCGAAGCGCCGTTGACCGACTGGGATGCCACCACA	JS17_1_Crispr_2_51	-
CAACCGAGGAGGGCGGATGCGGCGATAATGGCGGC	JS17_1_Crispr_2_52	-
TTGAAGGGCCCGGCGGGCGCGCCGCTCCAGGCAG	JS17_1_Crispr_2_53	-
GTCGGGAAGCTCACAGAATCCTGGCGCACGGTGGAC	JS17_1_Crispr_2_54	-
GGCGCCGCTCTGAACGCCGCTCGGGACGCTGCTTGGC	JS17_1_Crispr_2_55	-
ATCGTGGATCCGGGGAATGGCACGCGTACGGAGAT	JS17_1_Crispr_2_56	-
ACAGACACGTCAATGCCACGATCCATGTCGCTTCCTT	JS17_1_Crispr_2_57	-
CAACGCCCCTGCGACCTCGAAACGGCGCTGCGCAGG	JS17_1_Crispr_2_58	-
GGCCAATACTTCACGTTCGTGCCCACCCACCATCTG	JS17_1_Crispr_2_59	-
GTGAGCTCGGGCTGAGCGTGAGCCAGACAGCGGCAAAG	JS17_1_Crispr_2_60	-
GCATTTGCTCGCCCATTTGCTTCCTCCAATCTGCGT	JS17_1_Crispr_2_61	-
CGCACCAAGGCCCCGGCTCGGCGAGGGACGGTTGCCGT	JS17_1_Crispr_2_62	-
GTCATGGTGTGCGCACAGAAACCTAGAGTTCGGGCGG	JS17_1_Crispr_2_63	-
GCGAAAGAGCGATCATTCCGGGTATGGATCAAACC	JS17_1_Crispr_2_64	-
CGCACCACCCGCAAGGCGACG	JS18_PossibleCrispr_1_1	-
CCGGCAGCGAAGAAGCCAGCA	JS18_PossibleCrispr_1_2	-
CTGGAGCTTGCCCTTGGCGGCCACTGATCCGAAGA	JS18_PossibleCrispr_2_1	-
CTGGCCAGAAACCTCTACCCCGAGCCCCTTGA	JS18_PossibleCrispr_3_1	-
ACTGCACCGTCGGATCAGCCGGATAACCATTC	JS18_PossibleCrispr_4_1	-
AGGCAACCGAGGCGCCGTTGACCGACTGAGATGCCA	JS20_1_Crispr_3_1	-
CAGAGACGTAATCAGGCCCATCGTGTCATTAGACCAA	JS20_1_Crispr_3_2	-
CTCGTCAAACGTCAAGCTACAGGGTGCTAATGACGCCC	JS20_1_Crispr_3_3	-
CGGCCCACGTCGTCGGTGAGGACCCTTGACACGGG	JS20_1_Crispr_3_4	-
GAGGGTCCGTTGACACTAAGTGTCACATGGGGAAA	JS20_1_Crispr_3_5	-
GCCGAATCGGTCGATGCGGTGGGGTCGAGCTTCGAC	JS20_1_Crispr_3_6	B22, Doucette, E6, G4
TTACAACATGGATGTTGATGACAACGCAAGTACAT	JS20_1_Crispr_3_7	-
ACCAGCATATAGACCAGCATACGTACCAGCATATCGT	JS20_1_Crispr_3_8	-
CACCACACAGCTCGGAGAAGCAGACCGGTGGGGCCGT	JS20_1_Crispr_3_9	G4
ACCCAGCCATCCCACGGGCCCTCACCCTGAGCGCGTG	JS20_1_Crispr_3_10	-
AAGATCAAGATTCCTGACATTACTGGTCCGGATT	JS20_1_Crispr_3_11	-
CATCTAGCAATTGGTAAAAATCCAGTTCGCTCATCTG	JS20_1_Crispr_3_12	-
TTGATATCTTGAATCTCAAGCTCAGACCCAATATCAAG	JS20_1_Crispr_3_13	-
TTTAATCACATGGGGCGGATATAATCCGACCTATGACC	JS20_1_Crispr_3_14	-
TCACGCTCGCGCCCGGTACGGGCGACGGGATTCTCACC	JS20_1_Crispr_3_15	PFR1, PFR2
ACGTTAAAGTTCTAGGAGAAACACAATGAGTAACG	JS20_1_Crispr_3_16	-
ACTCCTACCGGCTTCCTCGCCGGCATCCTCGAAGT	JS20_1_Crispr_3_17	-
AGTCAGGATGCGTGGGTTCGCTCACACATGGACG	JS20_1_Crispr_3_18	-

TGCTTGCCGGGTGCGGAGGTCAATGATTACACCCGACT	JS20_1_Crispr_3_19	-
GACACCCCGTATGCCGTAGCTCAGCACGAGAACG	JS20_1_Crispr_3_20	-
AGCGGCGATCTGAGCGGCGTCCCAAGCAGCGCCATAA	JS20_1_Crispr_3_21	-
GCATCCGAAGCGGTGACCATTCCGGCGATCGTGCGGGGA	JS20_1_Crispr_3_22	PFR1, PFR2
GCAAAACGACCAAAAGAGACAAGCCCTGAGTAAGCAAA	JS20_1_Crispr_3_23	-
CGCGAGGTTCCCGTGGAAACCAGGAGTAAGCGTTCGA	JS20_1_Crispr_3_24	-
TGCGCCAAGTTCTTGGCGAGGATCTGCTCCTTGCCGTT	JS20_1_Crispr_3_25	-
GCGACCGGGGAAGAGAATTCGAGCGTACTCCAGCATG	JS20_1_Crispr_3_26	-
CTGTGGGGTGTGTGCATGGCGTCGAGTGAGGTCG	JS20_1_Crispr_3_27	-
CGCGAGGTTCCCGTGGTCTCGGGTGAAGTTGTGGTGCGACC	JS20_1_Crispr_3_28	-
TCGCTGGCTCGTGATCGCGGTGTCACCACAGGCGCCT	JS20_1_Crispr_3_29	-
ACGTCCAAGCATTTCAAGTTCTGCGCCAATGCGCGCAT	JS20_1_Crispr_3_30	PFR1, PFR2
AAGCTGGGTGGCAAGATCACCCGCACTGTCCAGGT	JS20_1_Crispr_3_31	-
GCATAGAGGCTGTAGATGCCATCGTCGGGGACGGT	JS20_1_Crispr_3_32	-
CCATCCTGGAAGAACTCGATGTCGTCATCCTTGTCGGAC	JS20_1_Crispr_3_33	-
AGCCGGTCCACGCCGTCGGCGGCCATGCCGGTGGT	JS20_1_Crispr_3_34	-
ATCAACCCCAATCGCGGCCAGTGGTATTCGCTGGCTCGT	JS20_1_Crispr_3_35	-
ATGGCGTCGAGTGAGGTCGAGCGGTTGCGGGCTG	JS20_1_Crispr_3_36	-
ATCGGTGGCGTTCAAGACCCGATCCCTGACATTCGC	JS20_1_Crispr_3_37	-
CATGAGATTGGGACACTTTCCTCCAGCGTTGTTCTG	JS20_1_Crispr_4_1	-
CGCGATGTCGCTCACATTGACATTGGCACATGGA	JS20_1_Crispr_4_2	-
GTCATCATCGGCTGGCCGTTGCTGAACGTTCTCGGT	JS20_1_Crispr_4_3	-
TGCGACACCGATTCGCTGCACCTGATCACCGACGA	JS20_1_Crispr_4_4	-
AGCCCGATCAGATCGCAGAGGACCGCACCCTACGCA	JS20_1_Crispr_4_5	-
AGGCTTCAGGTCCGGCCTCGGCCCACAGGATCGG	JS20_1_Crispr_5_1	-
CAGGGCCTGATCGAGCTTATTTATCGAGCAGGTGCC	JS20_1_Crispr_5_2	-
GCTTCCGTTGGTGAACACCATTTTGGTTTGATTCCACTC	JS20_1_Crispr_5_3	-
CTCGGCATGATGCCAGCGCCGATTATCGTGAGCGTATT	JS20_1_Crispr_5_4	-
AAGATGAGCGGGCCGAGGGGTTCGACCCGGGACGCGG	JS20_1_Crispr_5_5	-
TGATGCCCCAGGCCGGCGCGCAACTGCTCACCGAGCTG	JS20_1_Crispr_5_6	-
GTGAGCTCGGGCTGAGCGTGAGCCAGACAGCGGCAAAG	JS20_1_Crispr_5_7	-
AACCTGTGGGGCTTCCCGGTTGTCGTCACGACCGC	JS20_1_Crispr_5_8	-
GCGAAAGAGCGATCATTCCGGGTATGGATCAAACC	JS20_1_Crispr_5_9	-
CACGGACTTGCCCAGCAGTGTCGGCCTGCTTCAAC	JS20_1_PossibleCrispr_1_1	-
GGGGTCTGCAACGGGGTCAGCCCAACAAGCTGACCCACTGGATCCACCACA	JS20_1_PossibleCrispr_2_1	-
TGCCGGCTTGGCCGTGGACGCCGT	JS20_1_PossibleCrispr_6_1	-
TGCCAGCTTCTTGGCCGCCGGCTT	JS20_1_PossibleCrispr_6_2	-
CGTCGCCTTGCGGGTGGTGCG	JS20_1_PossibleCrispr_6_3	-
TGCCCACCTGGCCAGAAACCTCTACCCCGAGCCCCTTGA	JS21_1_PossibleCrispr_7_1	-
GGTTTGATCCATACCCGGAATGATCGCTCTTTCGC	JS21_1_Crispr_3_1	-
AATACGCTCACGATAATCGGCGCTGGCATCATGCCGA	JS21_1_Crispr_3_2	-
CACCCGCAGAGGCAGCACCAGGCCGAAGCGCGCCGACAT	JS21_1_Crispr_3_3	-
TTTTGCCAGATAGATGGACGTGCCCGGCGTCCACGACGA	JS21_1_Crispr_3_4	-

GAGTGGAATCAAACCAAAATGGTGTTCACCAACGGAAGC	JS21_1_Crispr_3_5	-
CTGTCGAAATGGCGTGAGGTGAGCGGCGGTGCAGG	JS21_1_Crispr_3_6	-
TGGCACCTGCTCGATAAATAAGCTCGATCAGGCCCTG	JS21_1_Crispr_3_7	-
CCGATCCTGTGGGCCGAGGCCGGACCTGAAGCCT	JS21_1_Crispr_3_8	-
TGCGTAGGGTGCGGTCCTCTGCGATCTGATCGGGCT	JS21_1_Crispr_4_1	-
TCGTCGGTGATCAGGTGCAGCGAATCGGTGTCGCA	JS21_1_Crispr_4_2	-
ACCGAGAACGTTCAGCAACGGCCAGCCGATGATGAC	JS21_1_Crispr_4_3	-
TGCCGTGTGGGGCGGTGGTGGTGCTGCCTCCCCAAT	JS21_1_Crispr_4_4	-
AACCACAGCTACGTCTTCCGGTGTCGGCGCGGATA	JS21_1_Crispr_4_5	-
TCCATGTGCCAATGTCAATGTGAGCGACATCGCG	JS21_1_Crispr_4_6	-
CAGAACAACGCTGGAGGAAAGTGTCCCAATCTCATG	JS21_1_Crispr_4_7	-
GCGAATGTCAGGGATCGGGTCTTGAACGCCACCGAT	JS21_1_Crispr_5_1	-
CAGCCCGCAACCGCTCGACCTCACTCGACGCCAT	JS21_1_Crispr_5_2	-
ACGAGCCAGCGAATACCACTGGCCGCGATTGGGGTTGAT	JS21_1_Crispr_5_3	-
GGCCTGCGCCTGGGGGTCGCCCCCGCGCATGAAGTG	JS21_1_Crispr_5_4	-
ACCACCGGCATGGCCGCCGACGGCGTGGACCGGCT	JS21_1_Crispr_5_5	-
GTCCGACAAGGATGACGACATCGAGTTCTTCCAGGATGG	JS21_1_Crispr_5_6	-
ACCGTCCCCGACGATGGCATCTACAGCCTCTATGC	JS21_1_Crispr_5_7	-
ACCTGGACAGTGCGGGTGATCTTGCCACCCAGCTT	JS21_1_Crispr_5_8	-
ATGCGCGCATTGGCGCAGAACTTGAAATGCTTGGACGT	JS21_1_Crispr_5_9	PFR1, PFR2
AGGCGCCTGTGGTGACACCGCGATCACGAGCCAGCGA	JS21_1_Crispr_5_10	-
CGACCTCACTCGACGCCATGCACACACCCCACAG	JS21_1_Crispr_5_11	-
CATGCTGGAGTACGCTCGAATTCTCTTCCCCGGTCGC	JS21_1_Crispr_5_12	-
AACGGCAAGGAGCAGATCCTCGCCAAGAACTTGGCGCA	JS21_1_Crispr_5_13	-
TCGAACGCTTACTCCTGGTTTCCACGGGAACCTCGCG	JS21_1_Crispr_5_14	-
TTTGCTTACTCAGGGCTTGTCTCTTTTGGTCGTTTTGC	JS21_1_Crispr_5_15	-
TCCCCGCACGATCGCCGGAATGGTCACCGCTTCGGATGC	JS21_1_Crispr_5_16	PFR1, PFR2
TTATGGCGCTGCTTGGGACGCCGCTCAGATCGCCGCT	JS21_1_Crispr_5_17	-
CGTTCTCGTGCTGAGCTACGGCATACGGGGTGTC	JS21_1_Crispr_5_18	-
AGTCGGGTGTAATCATTGACCTCCGCACCCGGCAAGCA	JS21_1_Crispr_5_19	-
CGTCCATGTGTGAGCGAACCCACGCATCCTGACT	JS21_1_Crispr_5_20	-
ACTTCGAGGATGCCGGCGAGGAAGCCGGTAGGAGT	JS21_1_Crispr_5_21	-
GCGATCTCGGCGCTGGTGGGGGCGTTCTCGATTGC	JS21_1_Crispr_5_22	Doucette, G4
CAGATGAGCGAACTGGATTTTTACCAATTGCTAGATG	JS21_1_Crispr_5_23	-
GCGAGCCAATCGACATCGCCCGGAAGGTTAGCGAGAAA	JS21_1_Crispr_5_24	Doucette, E6
CGGCATCATGTGATCACCCGACCCATGACCTGCTCCCACG	JS21_1_Crispr_5_25	-
CGTTACTCATTGTGTTTCTCCTAGAACTTTAACGT	JS21_1_Crispr_5_26	-
GGTGAGAATCCCGTCGCCCGTACCGGGCGCGAGCGTGA	JS21_1_Crispr_5_27	PFR1, PFR2
GGTCATAGGTCGGATTATATCCGCCCCATGTGATTAAA	JS21_1_Crispr_5_28	-
CTTGATATTGGGTCTGAGCTTGAGATTCAAGATATCAA	JS21_1_Crispr_5_29	-
CTGTGCAGTCCGTCTCCACCGTGGAAGCCGACAA	JS21_1_Crispr_5_30	-

AATCCGGACCAGTAATGTCAGGAATCTTGATCTT	JS21_1_Crispr_5_31	-
CACGCGCTCAGGGTGAGGGCCCGTGGGATGGCTGGGT	JS21_1_Crispr_5_32	-
CATTACTCATCGTGTTTCTCCCTAGAACTTTAGTGT	JS21_1_Crispr_5_33	-
CGGCTCGTTCACCGCATCCTTCGGGGGTGTCGGGGCAG	JS21_1_Crispr_5_34	-
GGCAGCCGCGATGGCTCTGGTTGTCTTGCGCATGCAA	JS21_1_Crispr_5_35	-
TTCAAGAATTTTGTTGAAACTTTTGTCGATTTCTACA	JS21_1_Crispr_5_36	-
CCGAATCGACGTAGACAAGCCAGGTCTTCTTTCCGG	JS21_1_Crispr_5_37	-
ACGCCCCCAGACAAAGCCGAACCTAGCGGAACTTGACC	JS21_1_Crispr_5_38	-
ATTGCCATTCGCTGCATCCCGCCAAGGGTGTCGGTA	JS21_1_Crispr_5_39	E6
CTGGAGCGAAGGCGGGCTCCACGCCGCTCCTTGA	JS21_1_PossibleCrispr_1_1	-
CGCACCACCCGCAAGGCGACG	JS21_1_PossibleCrispr_2_1	-
AAGCCGGCGGCCAAGAAGCCGGCA	JS21_1_PossibleCrispr_2_2	-
ACGGCGTCCACGGCCAAGCCGGCA	JS21_1_PossibleCrispr_2_3	-
AGCGCATGGGAGAAGGCCCGGCAGGACGCCGTGGAC	JS22_Crispr_2_1	-
GGCACCGGGCTCGACGTGGACCAGATGATCGCAA	JS22_Crispr_2_2	-
CGTCACCCTCAATCGTGGACAGAGTCGTCTTGCCCA	JS22_Crispr_2_3	-
GCATTTGCTTGCCCATTTGCTTCCTCCAATCTGCGT	JS22_Crispr_2_4	-
CGCACCAAGGCCCCGGCTCGGCGAGGGACGGTTGCCGT	JS22_Crispr_2_5	-
GTCATGGTGTGCGCACAGAAACCTAGAGTTCGGGCGG	JS22_Crispr_2_6	-
GCGAAAGAGCGATCATTCCGGGTATGGATCAAACC	JS22_Crispr_2_7	-
CGCACCACCCGCAAGGCGACG	JS22_PossibleCrispr_1_1	-
AAGCCGGCGGCCAAGAAGCCGGCA	JS22_PossibleCrispr_1_2	-
ACGGCGTCCACGGCCAAGCCGGCA	JS22_PossibleCrispr_1_3	-
GCCGCACAACTGCCCACCTGGCCAGAAACCTCTACCCCGAGCCCCTTGA	JS22_PossibleCrispr_3_1	-
TCAGAAACAGGCATACCAGATGTTAATGCAAGGGCTG	JS23_Crispr_2_1	-
GACAGGACCACAGGCACGGTCCTGGACCACAAGATC	JS23_Crispr_2_2	-
CTCGTGAGGACCTCACTGACCAGCATCTGCCGCTGAG	JS23_Crispr_2_3	phiB5
GCCCCGAACCCGACACCCACGACCACCAGAACACCCAC	JS23_Crispr_2_4	-
GTTGAGCAGGTGGCAGAATGTACATGCCACGAGACG	JS23_Crispr_2_5	-
TATACAGCCTTCTTCGTAGGAACGTCAACACCTTCAG	JS23_Crispr_2_6	-
GCGAAGGTCGGGCTGTTCAAGGACGACGGCGGCCCG	JS23_Crispr_2_7	-
CCCACCCCTACACAACAGACGGCACGAGCCTCACAG	JS23_Crispr_2_8	-
CATCCTGAATACATTCGGGCTTGAGGACCAACGGT	JS23_Crispr_2_9	-
GGTGGCCGTTCCGATTGGTTTCCGCCGTTCGATAAG	JS23_Crispr_2_10	-
ACGAAGACTGACCCACTCCAGGGACCGGCCCCCAAG	JS23_Crispr_2_11	-
ACGACGATCCATAGTTCCCCAGAACCTCTCTAGCA	JS23_Crispr_2_12	-
ACGTTCCGCACCCGGCGCGGGTATGACCACAACATT	JS23_Crispr_2_13	-
CCGAAGCTCGCTCCTCTGATGAATGCCATCACTTCAGCG	JS23_Crispr_2_14	-
GGGTCCGGTAACAGCGGGATCGGGCACCCTGCACCG	JS23_Crispr_2_15	-
CGAACCGAGCTGCGCAACAAGCCCCATTTCTTTGA	JS23_Crispr_2_16	-
GGGCAGGTGTGGGAAGACGACTGGGCAGAACTCGCTAA	JS23_Crispr_2_17	-
CCGGCTCTTCAAGGCTGTCCGACCAGACGGGGCCG	JS23_Crispr_2_18	-

CCCGACAAATTTCCTCGCAAGCGGGCCGCGCACGCG	JS23_Crispr_2_19	Anatole, B22, B3, E1, E6, G4; PJS23
TTCTCCTGAAATGCCGTTGTGGCATCAATAGTAGG	JS23_Crispr_2_20	-
TGGGCTTCCTCCTGTGTGAGCTGAATTTGCTCGACAG	JS23_Crispr_2_21	-
GGCGACAGTGCGCATGTGATCCGGTTCGGTCTTGA	JS23_Crispr_2_22	-
TCAAAGGAGCATGCCCATGAACACCACGGTTGTCGA	JS23_Crispr_2_23	-
GTGGGCGGCGAGGGCTTCCCAGCGGCGCAGGAGCCGGACC	JS23_Crispr_2_24	-
GTGAGCTCGGGCTGAGCGTGAGCCAGACAGCGGCAAAG	JS23_Crispr_2_25	-
GCATTTGCTTGCCCATTTGCTTCCTCCAATCTGCGT	JS23_Crispr_2_26	-
CGCACCAAGGCCCCGGCTCGGCGAGGGACGGTTGCCGT	JS23_Crispr_2_27	-
CGCACCACCCGCAAGGCGACG	JS23_PossibleCrispr_1_1	-
ACGGCAGCTGCCAAGAAGCCGGCA	JS23_PossibleCrispr_1_2	-
GCTACGTCCTCGGCGTCC	JS23_PossibleCrispr_1_3	-
ACTGAAACCAACTCCGCAGTATAACGAGCTTCCAT	JS25_Crispr_2_1	-
TACCGACACCCTTGGCGGGATGCAGCGAATGGCAAT	JS25_Crispr_2_2	E6
GGTCAAGTTCCGCTAGGTTCGGCTTTGTCTGGGGGCGT	JS25_Crispr_2_3	-
CCGGAAAGAAGACCTGGCTTGTCTACGTCGATTCGG	JS25_Crispr_2_4	-
TGTAGAAATCGACAAAAGTTTCAACAAAATTCTTGAA	JS25_Crispr_2_5	-
TTGCATGCGCAAGACAACCAGAGCCATCGCGGCTGCC	JS25_Crispr_2_6	-
CTGCCCCGACACCCCCGAAGGATGCGGTGAACGAGCCG	JS25_Crispr_2_7	-
ACACTAAAGTTCTAGGGAGAAACACGATGAGTAATG	JS25_Crispr_2_8	-
ACCCAGCCATCCCACGGGCCCTCACCCTGAGCGCGTG	JS25_Crispr_2_9	-
AAGATCAAGATTCCTGACATTACTGGTCCGGATT	JS25_Crispr_2_10	-
TTGTCGGCTTCCACGGTGGAGACGGACTGCACAG	JS25_Crispr_2_11	-
TTGATATCTTGAATCTCAAGCTCAGACCCAATATCAAG	JS25_Crispr_2_12	-
TTTAATCACATGGGGCGGATATAATCCGACCTATGACC	JS25_Crispr_2_13	-
TCACGCTCGCGCCCGGTACGGGCGACGGGATTCTCACC	JS25_Crispr_2_14	PFR1, PFR2
ACGTTAAAGTTCTAGGAGAAACACAATGAGTAACG	JS25_Crispr_2_15	-
CGTGGGAGCAGGTCATGGGTCGGGTGATCACATGATGCCG	JS25_Crispr_2_16	-
TTTCTCGCTAACCTTCCGGGCGATGTCGATTGGCTCGC	JS25_Crispr_2_17	Doucette, E6
CATCTAGCAATTGGTAAAAATCCAGTTCGCTCATCTG	JS25_Crispr_2_18	-
GCAATCGAGAACGCCCCCACCAGCGCCGAGATCGC	JS25_Crispr_2_19	PFR1, PFR2
ACTCCTACCGGCTTCCTCGCCGGCATCCTCGAAGT	JS25_Crispr_2_20	-
AGTCAGGATGCGTGGGTTCGCTCACACATGGACG	JS25_Crispr_2_21	-
TGCTTGCCGGGTGCGGAGGTCAATGATTACACCCGACT	JS25_Crispr_2_22	-
GACACCCCGTATGCCGTAGCTCAGCACGAGAACG	JS25_Crispr_2_23	-
AGCGGCGATCTGAGCGGCGTCCCAAGCAGCGCCATAA	JS25_Crispr_2_24	-
GCATCCGAAGCGGTGACCATTCCGGCGATCGTGCGGGGA	JS25_Crispr_2_25	PFR1, PFR2
GCAAAACGACCAAAAGAGACAAGCCCTGAGTAAGCAAA	JS25_Crispr_2_26	-
CGCGAGGTTCCCGTGGAAACCAGGAGTAAGCGTTCGA	JS25_Crispr_2_27	-

TGCGCCAAGTTCTTGGCGAGGATCTGCTCCTTGCCGTT	JS25_Crispr_2_28	-
GCGACCGGGGAAGAGAATTCGAGCGTACTCCAGCATG	JS25_Crispr_2_29	-
CTGTGGGGTGTGTGCATGGCGTCGAGTGAGGTCG	JS25_Crispr_2_30	-
TCGCTGGCTCGTGATCGCGGTGTCACCACAGGCGCCT	JS25_Crispr_2_31	-
ACGTCCAAGCATTTCAAGTTCTGCGCCAATGCGCGCAT	JS25_Crispr_2_32	PFR1, PFR2
AAGCTGGGTGGCAAGATCACCCGCACTGTCCAGGT	JS25_Crispr_2_33	-
GCATAGAGGCTGTAGATGCCATCGTCGGGGACGGT	JS25_Crispr_2_34	-
CCATCCTGGAAGAACTCGATGTCGTCATCCTTGTCGGAC	JS25_Crispr_2_35	-
AGCCGGTCCACGCCGTCGGCGGCCATGCCGGTGGT	JS25_Crispr_2_36	-
CACTTCATGCGCGGGGGCGACCCCCAGGCGCAGGCC	JS25_Crispr_2_37	-
ATCAACCCCAATCGCGGCCAGTGGTATTCGCTGGCTCGT	JS25_Crispr_2_38	-
ATGGCGTCGAGTGAGGTCGAGCGGTTGCGGGCTG	JS25_Crispr_2_39	-
ATCGGTGGCGTTCAAGACCCGATCCCTGACATTCGC	JS25_Crispr_2_40	-
CATGAGATTGGGACACTTTCCTCCAGCGTTGTTCTG	JS25_Crispr_3_1	-
CGCGATGTCGCTCACATTGACATTGGCACATGGA	JS25_Crispr_3_2	-
TATCCGCGCCGACACCGGAAGACGTAGCTGTGGTT	JS25_Crispr_3_3	-
ATTGGGGAGGCAGCACCACCACCGCCCCACACGGCA	JS25_Crispr_3_4	-
TGCGACACCGATTCGCTGCACCTGATCACCGACGA	JS25_Crispr_3_5	-
AGCCCGATCAGATCGCAGAGGACCGCACCCTACGCA	JS25_Crispr_3_6	-
AGGCTTCAGGTCCGGCCTCGGCCCACAGGATCGG	JS25_Crispr_4_1	-
CAGGGCCTGATCGAGCTTATTTATCGAGCAGGTGCC	JS25_Crispr_4_2	-
CCCTGCACCGCCGCTCACCTCACGCCATTTCGACAG	JS25_Crispr_4_3	-
GCTTCCGTTGGTGAACACCATTTTGGTTTGATTCCACTC	JS25_Crispr_4_4	-
TCGTCGTGGACGCCGGGCACGTCCATCTATCTGGCAAA	JS25_Crispr_4_5	-
CATGTCGGCGCGCTTCGGCCTGGTGCTGCCTCTGCGGGTG	JS25_Crispr_4_6	-
TCGGCATGATGCCAGCGCCGATTATCGTGAGCGTATT	JS25_Crispr_4_7	-
GCGAAAGAGCGATCATTCCGGGTATGGATCAAACC	JS25_Crispr_4_8	-
CGCACCACCCGCAAGGCGACG	JS25_PossibleCrispr_1_1	-
AAGCCGGCGGCCAAGAAGCCGGCA	JS25_PossibleCrispr_1_2	-
ACGGCGTCCACGGCCAAGCCGGCA	JS25_PossibleCrispr_1_3	-
TCAAGGAGCGGCGTGGAGCCCGCCTTCGCTCCAG	JS25_PossibleCrispr_5_1	-
