# Supplementary material for: De novo assembly of genomes from long sequence reads reveals uncharted territories of Propionibacterium freudenreichii
Source: BMC Genomics. 2017 Oct 16;18:790. doi: 10.1186/s12864-017-4165-9 (PMC5644110; doi:10.1186/s12864-017-4165-9)
Supplement: Supplementary file 28 — The alignment of the three predicted B12 riboswitches with the conserved B12-box highlighted. (PDF 28 kb) [file 12864_2017_4165_MOESM28_ESM.pdf]

The B12 riboswitch located upstream two B12 operons and the B12-dependent Methylmalonyl Co-A mutase (*mutA*). The conserved sequence SAGYCMSAMRMBCYGCCD constituting the B12-box is framed.

```
B12el_cbiL1681393-1681540_JS2_rev      -CCGGATGATCGATGTGGAAGACTGAGCGCGGCAACGAGCGCG--AGGAATGCCGGTGAC
B12el_cbiB_1203248-1203397_JS2_re      ---------------CGGCCAGC---GCGCGTCCGC---------AGCGAAGCCGGT---
B12el_mutA_1681647-1681844_JS2_fw      CTAGTAGTGCTGGTTCGGCTGCC---CCACGGCAGTCGTCGCAAGAGGGAATCCGGT---
                                                      **    *    * ** *            **  *  *****

B12el_cbiL1681393-1681540_JS2_rev      CGGGGGCCATGGCCGCCCGGGAGTCCGCGACGGTCCCGCCACTGTGAGCCGGTGAAGCC-
B12el_cbiB_1203248-1203397_JS2_re      ------------------GGGAATCCGGCACTGTCCCGCAACGGTGATGGGGCCCGGCCC
B12el_mutA_1681647-1681844_JS2_fw      ------------------GTAATTCCGGAACTGTCCCGCAGCGGTCAATGGGAACGACAC
                                                        *  * ****  ** *******  * ** *   **     *

B12el_cbiL1681393-1681540_JS2_rev      ------------------------------------------------------------
B12el_cbiB_1203248-1203397_JS2_re      CGAGTCCGGT------------CGACTG---------------------TGGACGTGTCC
B12el_mutA_1681647-1681844_JS2_fw      AACGTAAGGCACTGGGCGGCAACGCCTGGGAAGTAGTAGTGGAGGAAGTCGGGAGTGATC

B12el_cbiL1681393-1681540_JS2_rev      ------------------GGCGAGTCAGACACTCCGCCGGTGCCGCTGAC
B12el_cbiB_1203248-1203397_JS2_re      CCACATTGCCCCGGCGCAAGGTCAGCCCCACGAGCTGCCTGCGCGTGCACC
B12el_mutA_1681647-1681844_JS2_fw      TCGCAATGCCC---------ATGAGTCCGAAGACCTGCCAGCAGCGACAAC
                                                              ** *  *    * *** *         *
```

The BLASTn alignment table for all of the B12 elements identified from the newly sequenced *P. freudenreichii* strains and the *P. freudenreichii* DSM 20271 (JS16).

qseqid	sseqid	pident	length	mismatch	gapopen	qstart	qend	sstart	send	evalue	bitscore
B12el_CbiL	JS9	100	148	0	0	1	148	1841300	1841447	3.00E-73	274
B12el_CbiL	JS8	100	148	0	0	1	148	1754668	1754815	3.00E-73	274
B12el_CbiL	JS7	100	148	0	0	1	148	1763169	1763316	3.00E-73	274
B12el_CbiL	JS4	100	148	0	0	1	148	1384368	1384515	3.00E-73	274
B12el_CbiL	JS2	100	148	0	0	1	148	1681393	1681540	3.00E-73	274
B12el_CbiL	JS25	100	148	0	0	1	148	1607956	1608103	3.00E-73	274
B12el_CbiL	JS21	100	148	0	0	1	148	1746056	1746203	3.00E-73	274
B12el_CbiL	JS20	100	148	0	0	1	148	971625	971478	3.00E-73	274
B12el_CbiL	JS18	100	148	0	0	1	148	1296043	1295896	3.00E-73	274
B12el_CbiL	JS16	100	148	0	0	1	148	852708	852561	3.00E-73	274
B12el_CbiL	JS14	100	148	0	0	1	148	1666592	1666739	3.00E-73	274
B12el_CbiL	JS13	100	148	0	0	1	148	1705557	1705704	3.00E-73	274
B12el_CbiL	JS12	100	148	0	0	1	148	1751280	1751427	3.00E-73	274
B12el_CbiL	JS11	100	148	0	0	1	148	1705587	1705734	3.00E-73	274
B12el_CbiL	JS10	100	148	0	0	1	148	1602802	1602949	3.00E-73	274
B12el_CbiL	JS23	99.3	143	0	1	1	142	1695548	1695690	3.00E-68	257
B12el_CbiL	JS22	99.3	143	0	1	1	142	1728974	1729116	3.00E-68	257
B12el_CbiL	JS17	99.3	143	0	1	1	142	1797087	1797229	3.00E-68	257
B12el_CbiL	JS15	99.3	143	0	1	1	142	877850	877708	3.00E-68	257
B12el_cbiB	JS8	100	150	0	0	1	150	1233114	1233263	2.00E-74	278

B12el_cbiB	JS7	100	150	0	0	1	150	1246455	1246604	2.00E-74	278
B12el_cbiB	JS2	100	150	0	0	1	150	1203248	1203397	2.00E-74	278
B12el_cbiB	JS25	100	150	0	0	1	150	1091201	1091350	2.00E-74	278
B12el_cbiB	JS23	100	150	0	0	1	150	1160583	1160732	2.00E-74	278
B12el_cbiB	JS22	100	150	0	0	1	150	1235175	1235324	2.00E-74	278
B12el_cbiB	JS21	100	150	0	0	1	150	1237340	1237489	2.00E-74	278
B12el_cbiB	JS18	100	150	0	0	1	150	1786790	1786641	2.00E-74	278
B12el_cbiB	JS17	100	150	0	0	1	150	1280961	1281110	2.00E-74	278
B12el_cbiB	JS16	100	150	0	0	1	150	1124460	1124609	2.00E-74	278
B12el_cbiB	JS15	100	150	0	0	1	150	1371988	1371839	2.00E-74	278
B12el_cbiB	JS14	100	150	0	0	1	150	1189715	1189864	2.00E-74	278
B12el_cbiB	JS13	100	150	0	0	1	150	1195116	1195265	2.00E-74	278
B12el_cbiB	JS12	100	150	0	0	1	150	1191080	1191229	2.00E-74	278
B12el_cbiB	JS11	100	150	0	0	1	150	1195146	1195295	2.00E-74	278
B12el_cbiB	JS10	100	150	0	0	1	150	1112048	1112197	2.00E-74	278
B12el_cbiB	JS4	99.33	150	0	1	1	150	877469	877617	3.00E-72	270
B12el_cbiB	JS9	98.67	150	2	0	1	150	1268797	1268946	4.00E-71	267
B12el_cbiB	JS20	98.67	150	2	0	1	150	1510211	1510062	4.00E-71	267
B12el_cbiB	JS20	98.67	150	2	0	1	150	1506091	1505942	4.00E-71	267
B12el_MutA	JS9	100	198	0	0	1	198	1841554	1841751	6.00E-101	366
B12el_MutA	JS8	100	198	0	0	1	198	1754922	1755119	6.00E-101	366
B12el_MutA	JS7	100	198	0	0	1	198	1763423	1763620	6.00E-101	366
B12el_MutA	JS4	100	198	0	0	1	198	1384622	1384819	6.00E-101	366
B12el_MutA	JS2	100	198	0	0	1	198	1681647	1681844	6.00E-101	366
B12el_MutA	JS25	100	198	0	0	1	198	1608210	1608407	6.00E-101	366
B12el_MutA	JS23	100	198	0	0	1	198	1695799	1695996	6.00E-101	366
B12el_MutA	JS22	100	198	0	0	1	198	1729225	1729422	6.00E-101	366
B12el_MutA	JS21	100	198	0	0	1	198	1746310	1746507	6.00E-101	366
B12el_MutA	JS20	100	198	0	0	1	198	971371	971174	6.00E-101	366
B12el_MutA	JS18	100	198	0	0	1	198	1295789	1295592	6.00E-101	366
B12el_MutA	JS17	100	198	0	0	1	198	1797338	1797535	6.00E-101	366
B12el_MutA	JS16	100	198	0	0	1	198	852454	852257	6.00E-101	366
B12el_MutA	JS15	100	198	0	0	1	198	877599	877402	6.00E-101	366
B12el_MutA	JS14	100	198	0	0	1	198	1666846	1667043	6.00E-101	366
B12el_MutA	JS13	100	198	0	0	1	198	1705811	1706008	6.00E-101	366

B12el_MutA	JS11	100	198	0	0	1	198	1705841	1706038	6.00E-101	366
B12el_MutA	JS10	100	198	0	0	1	198	1603056	1603253	6.00E-101	366
B12el_MutA	JS12	98.99	198	2	0	1	198	1751534	1751731	1.00E-97	355
